# Matrix stiffness promotes colorectal cancer progression via Golgi-dependent cholesterol synthesis

**DOI:** 10.1038/s41419-026-09101-5

**Published:** 2026-07-24

**Authors:** Min-Yue Yin, Xue Li, Jun-Qi Zhang, Bo-Shen Lin, Chang-Hao Zhang, Yu-Hang Liu, Shu-Tian Zhang, Si-An Xie

**Affiliations:** 1 Department of Gastroenterology, Beijing Friendship Hospital, Capital Medical University, State Key Laboratory of Digestive Health, National Clinical Research Center for Digestive Diseases, Beijing Key Laboratory for Precancerous Lesion of Digestive Diseases, Beijing, PR China; 2 Department of Gastroenterology, The Fourth Affiliated Hospital of Soochow University, Suzhou Dushu Lake Hospital, Medical Center of Soochow University, Suzhou, PR China; 3 Department of Gastroenterology and Hepatology, Sichuan Provincial People’s Hospital, School of Medicine, University of Electronic Science and Technology of China, Chengdu, PR China

**Keywords:** Cancer metabolism, Cancer microenvironment, Protein translocation

## Abstract

Extracellular matrix (ECM) stiffening and metabolic reprogramming are hallmarks of colorectal cancer (CRC) progression; however, the mechanistic link between biomechanical cues and cholesterol metabolism remains poorly defined. Here, we delineate a mechano-metabolic pathway in which matrix stiffness activates the mechanosensitive Ca²⁺ channel Piezo1 to drive structural reorganization of the Golgi apparatus, promoting its close apposition with the endoplasmic reticulum (ER). This organelle remodeling facilitates the proteolytic activation and nuclear translocation of SREBP2, thereby transcriptionally upregulating HMGCR, the rate-limiting enzyme for cholesterol synthesis, and elevating intracellular cholesterol. HMGCR upregulation is functionally essential for stiffness-induced epithelial-mesenchymal transition (EMT) and CRC progression. Mechanistically, HMGCR-mediated cholesterol accumulation promotes phosphorylation of β-catenin at Ser552, which disrupts β-catenin/E-cadherin complexes at adherens junctions, leading to β-catenin nuclear accumulation and EMT execution. Therapeutically, combined pharmacological inhibition of matrix crosslinking and cholesterol synthesis synergistically suppresses tumor growth and invasion while restoring epithelial architecture in vivo. Collectively, these findings reveal that Piezo1-dependent Golgi-ER reorganization triggers SREBP2-driven cholesterol synthesis, ultimately fueling β-catenin-mediated junctional disassembly and metastasis, and highlight the therapeutic promise of co-targeting tumor mechanics and metabolism.

## Introduction

Colorectal cancer (CRC) is a leading cause of cancer‑related mortality worldwide [1], with its progression governed by dynamic and reciprocal interactions within the tumor microenvironment (TME). Among the biomechanical alterations in the TME, extracellular matrix (ECM) stiffening has emerged as a critical driver of tumor aggressiveness, therapy resistance, and poor prognosis [2]. Previous studies have demonstrated that tissue stiffness ranges from ~1 to 2 kPa in normal colonic mucosa to over 20 kPa in invasive carcinomas [3]. Increased matrix rigidity does not merely provide passive structural support, it actively transmits mechanosensory signals through integrin‑mediated adhesions and cytoskeletal remodeling, thereby activating downstream oncogenic pathways [4–6]. These pathways collectively promote cancer cell proliferation, survival, invasion, and epithelial‑mesenchymal transition (EMT). Moreover, stiffened ECM acts as a physical barrier that impedes immune cell infiltration and compromises drug delivery, further fostering an immunosuppressive and therapy‑resistant niche [7–9]. Consequently, elevated matrix rigidity has emerged as a key biomechanical feature of therapy-resistant CRC.

Among the key mechano-transducers that respond to matrix stiffening, the mechanosensitive ion channel Piezo1 has garnered significant attention. Piezo1, a pore-forming subunit of mechanically activated cation channels, mediates rapid calcium (Ca²⁺) influx [10]. Intriguingly, matrix stiffness has been identified as a potent activator of Piezo1 and the stiffness-Piezo1-Ca²⁺ axis has subsequently been implicated in the progression of multiple malignancies [11–13]. These findings indicate that Piezo1 serves as a critical molecular sensor through which tumor cells perceive and respond to mechanical stiffening, might be a critical therapeutic target.

Beyond mechanical alterations, tumors undergo metabolic reprogramming to support rapid growth and immune evasion through adaptive changes in nutrient utilization and signaling pathways across various cell types within the TME [14, 15]. In particular, dysregulation of cholesterol homeostasis—orchestrated mainly by the transcription factor SREBP2 and its downstream target HMGCR, the rate-limiting enzyme of the mevalonate pathway—has been implicated in facilitating oncogenic processes such as apoptosis resistance, enhanced DNA repair, and dynamic membrane remodeling [16, 17]. Hyperactivation of SREBP2/HMGCR signaling and subsequent cholesterol overproduction contribute significantly to CRC progression, metastasis, and serrated tumorigenesis [18–22]. Although statins, which inhibit HMGCR, have shown promising antitumor effects beyond cholesterol-lowering efficacy, but their clinical benefits in oncology remain controversial [23, 24]. This is partly due to their context-dependent duality, capable of inducing cancer cell death by cholesterol starvation or, paradoxically, triggering feedback circuits that ultimately drive resistance and progression. Additionally, the mechanisms of mechanical-metabolic crosstalk are inconsistent. For instance, attenuated actomyosin contractility or a soft ECM microenvironment inhibits Lipin-1, resulting in SREBP2 activation across diverse cell types, such as mammary and retinal epithelial cells, breast cancer cells, fibroblasts, and human pluripotent stem cells [25]. However, one study demonstrates that shear stress directly activates SREBP1 in endothelial cells via an integrin/focal adhesion kinase dependent pathway [26]. Consequently, defining the diverse upstream mechanical cues that govern cholesterol metabolic rewiring is pivotal for developing novel therapeutic strategies of CRC treatment.

Aberrant Golgi dynamics, encompassing both morphological and functional alterations, have been proved to enhance the invasive and metastatic potential of cancer by modulating protein trafficking, process, and secretion [27–29]. The fragmented Golgi, characterized by dilated cisternae, loss of linear organization, and reduced transport vesicles, has been observed in CRC tissues and breast cancer cells [30]. Certain gastric cancer cells also exhibit similar Golgi morphography, while others display opposite condensed structures compared to normal gastric cells [31]. However, the two distinct Golgi phenotypes can lead to similar cancer-promoting consequences. For instance, gastric cancer cells with condensed Golgi show increased malignancy and metastasis through enhancing microtubules and vesicular trafficking [31]. Consistent with this study, Golgi fragmentation blocks tumor growth and migration in pancreatic ductal carcinoma [32]. In contrast, alcohol-induced Golgi fragmentation promotes prostate cancer proliferation [28]. Chemotherapy-induced Golgi dispersal also defenses cells survival by augmenting protein trafficking in CRC [33]. Therefore, conflicting evidence exists regarding the role of the Golgi apparatus in tumor progression, which may be attributed to differences in the inducing factors of Golgi disorganization and the specific Golgi-associated functions involved. Research on how mechanical stimuli influence Golgi function are currently limited. Recent evidence indicates matrix stiffness promotes post-Golgi cargo sorting to direct proteins toward the secretory pathway instead of lysosomal degradation [34]. Similarly, mechanical tension also enhances trafficking efficacy from the endoplasmic reticulum (ER) to Golgi [35]. Despite these advances, how ECM stiffening couples to alterations in organelle dynamics and orchestrates cholesterol metabolic reprogramming to facilitate CRC progression remains elusive.

In this study, we investigate this mechano‑metabolic crosstalk in CRC. We demonstrate that matrix stiffening promotes EMT and invasion through upregulation of HMGCR‑dependent cholesterol biosynthesis. Mechanistically, we uncover a novel Golgi‑ER fusion‑dependent mechanism that stiff substrates trigger Golgi-ER fusion, which facilitates the nuclear translocation of SREBP2, and transcriptional induction of HMGCR. Functionally, HMGCR promotes EMT by disrupting E‑cadherin/β‑catenin complexes and promoting E‑cadherin degradation, thereby releasing β‑catenin for nuclear signaling. Importantly, combined targeting of ECM stiffness and cholesterol synthesis synergistically suppresses tumor progression in preclinical models. Our work establishes a Golgi‑ER fusion‑mediated mechano‑metabolic axis that underlies CRC progression and highlights a promising therapeutic strategy for stiffness‑driven malignancies.

## Materials and methods

### Cell lines

Cell lines (HCT116, HCT15, SW480, HT29, MC38) were sourced from the Cell Bank of Type Culture Collection of the Chinese Academy of Sciences. HCT116 and HT29 cells were cultured in McCoy’s 5A medium (Eallbio) while HCT15 and SW480 cells in RPMI 1640 medium (Eallbio), supplemented with 10% fetal bovine serum (FBS) and antibiotics (penicillin/streptomycin, 1000 U/ml). MC38 cells were cultured in DMEM (Eallbio) with 10% FBS, 1X nonessential amino acids and 1X sodium pyruvate. All cell lines were cultured in a humidified incubator at 37 °C with 5% CO_2_.

### Cell transfection

Overexpression was induced using the pcDNA3.1-3xFlag-HMGCR, and pcDNA3.1-HA-SREBF2 plasmids (Unibio, China), which were transfected using MegaTran 2.0 (Origene, USA). Si-HMGCR (Hippo Biotechnology, China) were transfected using siTran 2.0 (Origene, USA). EZ Cap^TM^ ER-mScarlet Probe mRNA (APExBIO, USA) was transfected using Lipo 2000 (Invitrogen, USA).

### Reagent treatment

Brefeldin A (MedChemExpress, USA), Fatostatin (MedChemExpress, USA), Simvastatin (MedChemExpress, USA), Mepanipyrim (Yuanye, China), GsMTx4 (Selleck, USA), and Yoda1 (Selleck, USA) were respectively dissolved in DMSO and added to the medium in indicated experiments. Equal concentration of DMSO was added in the control group.

### Polyacrylamide (PA) gel preparation

PA gels were prepared as previously described [36]. Briefly, 40% w/v acrylamide and 2% w/v bis-acrylamide stock solutions (Bio-Rad) were mixed, supplemented with 10% Ammonium Persulfate. Different stiffness of gels was regulated by different final concentration of bis-acrylamide (0.048% and 0.264%) for mimicking the elastic moduli of 2.61 ± 0.82 and 19.66 ± 1.19 kPa. For gel polymerization, N, N, N9, N9-tetramethylethylenediamine (TEMED, Bio-Rad) solution was added to the mixture. The thickness of the gel has been confirmed through imaging of embedded fluorescent beads (Invitrogen, USA) in the gel with confocal microscope in our earlier work. To cross-link extracellular matrix proteins onto the gel surface, the gel was exposed to the heterobifunctional cross-linker sulfo-SANPAH (Abcam, 0.5 mg/ml) under UV 264 nm light for 15 min. After being cleaned with sterile PBS, each gel was coated with 1 ml of collagen Type I (0.1 mg/mL, from rat tail, Corning) at room temperature for 1 hour. Following washed up with sterile PBS, cells were seeded onto each gel and incubated a humidified atmosphere with 5% CO_2_ at 37 °C.

### RNA isolation and quantitative real-time PCR (RT-qPCR)

Total RNA was extracted using the TRIzol reagent (Ambion, USA) and reverse-transcribed into cDNA using the PrimeScript™ RT Master Mix (Takara, Japan). RT-qPCR was performed using the Fast SYBR Green Master Mix (Invitrogen, USA) and run on the 7500 Fast Real-Time PCR System (Applied Biosystems, USA). The RNA analysis data were normalized by GAPDH and based on 2^−ΔΔCt^ calculations. All the primer details are listed in the Supplementary Table S1.

### Protein extraction and Western blot (WB)

Protein lysates were prepared in RIPA lysis buffer (Beyotime, China) with protease inhibitor cocktail (Roche, Switzerland) and phosphatase inhibitor (CWBIO, China). The BCA Protein Assay Kit (Biosharp, China) was used for protein quantification. Equivalent amounts of protein were run on 7.5% or 10% sodium dodecyl sulfate-polyacrylamide gels electrophoresis (Epizyme, China) and transferred to 0.22 μm polyvinylidene fluoride (PVDF) membranes (Immobilon, USA). Then the blots were blocked with 5% skim milk (Biosharp, China) and incubated with specific primary antibodies at 4 °C overnight. The protein bands were imaged and quantified using the ChemiDoc XRS + system (Bio-Rad, USA). All the antibodies are listed in the Supplementary Table S2. The full and uncropped western blots are presented in the Supplemental Material.

### Co-immunoprecipitation (Co-IP)

After indicated treatment, the cells were homogenized in cell lysis buffer for Western and IP (Beyotime, China), supplemented with protease (Roche, Switzerland) and phosphatase inhibitors (CWBIO, China). The homogenates were centrifuged at 12,000 × rpm for 15 min at 4 °C and the cell lysates were collected. The equivalent specific primary antibodies and normal IgG solution were added to the cell lysates, allowing the protein to interact with the antibodies at 4 °C overnight. The next day, Protein A + G Magnetic Beads (Beyotime, China) were added to the cell lysates after being washed with 1X TBS three times and then incubated for two hours at room temperature. The beads were washed with 1X TBS three times and then supplemented with 1X loading buffer for subsequent western blot.

### Nucleoplasm separation experiments

The Nuclear and Cytoplasmic Protein Extraction Kit (Beyotime, China) was used according to the manufacturer’s instructions. Briefly, after indicated treatment, the cells were homogenized in ice-cold cytoplasmic protein extraction reagent A and cytoplasmic protein extraction reagent B containing protease (Roche, Switzerland) and phosphatase inhibitors (CWBIO, China). Then the homogenates were centrifuged at 12,000–16,000 × *g* for 5 min at 4 °C. The supernatant was cytoplasmic proteins and the sediment was extracted as nuclei proteins with the Nuclear Protein Extraction Reagent.

### Immunofluorescence (IF)

The cells were seeded on 20 mm diameter coverslips and given different treatments for indicated time. Then the cells were fixed in 4% paraformaldehyde for 30 min, permeabilized with 0.3% Triton X-100 for 20–30 min, blocked with 1% BSA (Solarbio, China) for 1 h at room temperature, and stained with primary antibodies overnight at 4 °C. Subsequently, the secondary antibody conjugated to Alexa Fluor® 488 or Alexa Fluor® 594 (Invitrogen, USA) was used for incubation for 1 h at room temperature in the dark, followed by DAPI (Santa Cruz, USA). Observation was made using a fluorescence confocal microscope (Olympus FluoView™ FV1200, Japan).

### H&E staining and immunohistochemistry (IHC)

For H&E staining, the tumor tissues were evicted, immersed in 10% neutral buffered formalin solution, and embedded in paraffin. Subsequently, tissue sections (4 μm) were cut, stained with hematoxylin and eosin, and assessed under the light microscope (Olympus, Japan).

For IHC staining, the slides were deparaffinized in xylene and then rehydrated in alcohol, after incubation at 65 °C for 2 h. Following heat-mediated antigen retrieval with Tris-EDTA buffer (pH 9.0) or citrate antigen retrieval solution, endogenous peroxidase activity was blocked with 3% H_2_O_2_ for 10 min. Sections were blocked by goat serum at room temperature for 1 h, incubated with the primary antibody at 4 °C overnight, and subsequent incubated with the secondary antibody at room temperature for 1 h. The immunosignal was visualized by diaminobenzidine kit (DAB, ZSGB-BIO, China). Finally, the tissues were counterstained with hematoxylin and photographed under the light microscope.

### Transmission electron microscope (TEM)

Pre‑stimulated cells (≥1 × 10⁶) were fixed with 2.5% Gluta fixative at 4 °C for 2–4 h, then embedded in 1% agarose. After post‑fixation with 1% OsO₄ in 0.1 M PB (pH7.4) for 2 h at room temperature, the samples were dehydrated in a graded ethanol series (30–100%) and acetone, followed by infiltration with epoxy resin. Polymerization was performed at 60 °C for 48 h. Ultrathin sections were cut and double-stained with uranyl acetate. The sections were observed under a TEM.

### Cell proliferation assays

For Cell Counting Kit-8 (CCK-8) assay, HCT116 and HCT15 cells, respectively transfected with oe-HMGCR and treated with the specific inhibitor, namely Simvastatin, were seeded at a density of 1000 cells per well in 100 μl of respective medium in 96-well plates. Every 24 h for 96 h, the 10% CCK8 kit reagent (Beyotime Biotechnology, China) was added to each well, and cell viability was measured with spectrophotometry at OD_450nm_ after two-hour-incubation.

The 5-ethynyl-2-deoxyuridine (EdU) assay was performed using the Cell-Light™ EdU Apollo® 567 kit (Ribobio, China) according to the manufacturer’s instructions. Briefly, the transfected cells were seeded at a density of 2.0 × 10^5^ in 12-well plates. After the three-hour incubation with EdU, Apollo and Hoechst staining were performed. EdU-positive cells were calculated on the formula: the number of Apollo-stained cells/total number of Hoechst-stained cells.

### Transwell migration assay

The 40,000 transfected cells were suspended in 200 μl serum-free medium and seeded into the upper compartment of the transwell chambers with polycarbonate membranes of 8 μm pore size (Corning Costar). The lower compartment was filled with 700 μl of complete medium. Following 48 h of incubation, invasive cells on the lower surface of the membrane were fixed and stained with crystal violet, and counted using ImageJ software.

### Total cholesterol measurement

For total cholesterol measurement of cells, the Amplex™ Red cholesterol assay kit (Thermo Fisher Scientific, USA) was used according to the manufacturer’s instructions. Briefly, indicated cells were incubated in 1X Reaction Buffer and a working solution of 300 μM Amplex® Red reagent for 30 min at 37 °C, protected from light. The fluorescence was measured by a fluorescence microplate reader using excitation in the range of 530–560 nm and emission detection at ~590 nm.

For total cholesterol measurement of serum, a Total Cholesterol Assay Kit (Leagene, China) was used according to the manufacturer’s instructions. Briefly, equivalent serum of animal serum was added into 300 μL COD-POD working solution containing Reagent A and B (1:1). A microplate reader conducts colorimetric determination at a range of 500–520 nm.

### Filipin III and BODIPY staining

For staining of free cholesterol with Filipin III (Sigma, USA) and lipid droplets with BODIPY 493/503 (GlpBio, USA) staining, different-stiffness-stimulated cells were washed with 1X PBS, fixed with 4% paraformaldehyde, and stained using 50 μg/mL Filipin and 5 μM BODIPY for 30 min in the dark at room temperature. The cells were also incubated with DAPI (Santa, USA) to stain the nuclei. Observation was made using fluorescence confocal microscope (Olympus FluoView™ FV1200, Japan).

### Rhodamine phalloidin staining

Cells seeded on PA gels of various stiffness were washed by PBS three times, fixed in 4% paraformaldehyde for 10 min at room temperature, and permeabilized in 0.3% Triton X-100 for 5 min. Following washed with PBS once, working stock of rhodamine phalloidin (100 nM, Cytoskeleton Inc., USA) was added to incubate cells at room temperature in the dark for 30 min. Nuclei were counterstained with DAPI, and observation was made using fluorescence confocal microscope (Olympus FluoView™ FV1200, Japan).

### ER tracker and Golgi staining

After Indicated treatment, cell culture medium was removed and the cells were washed by 1X PBS twice, followed by pre-incubation of the Golgi-Tracker Green II Working Solution (Beyotime, C1885S) at 4 °C for 30 minutes in the dark. Next, remove the working solution and wash the cells twice using 1X PBS. Add the ER-Tracker Red working solution (Beyotime, C1041S) at 37 °C for 30 min. Lastly, incubate the cells using Hoechst 33342 (1X) in the dark for 10 min. The cells were examined using fluorescence confocal microscope (Olympus FluoView™ FV1200, Japan) and a spinning disk confocal and optical diffraction tomography (ODT) dual-mode microscope (Cell Xelence, CSR Biotech, China).

### Dual-luciferase reporter assay

The sequence of HMGCR promoter was inserted into pGL3-Basic plasmid. Cells were cultured to ~50–60% confluence and co-transfected with luciferase reporter plasmid and HMGCR promoter plasmid. After 24 h, the cells were seeded onto PA gels of various stiffness. After 48 h, the activity of luciferase was measured according to the manufacturer’s instructions.

### Chromatin immunoprecipitation (ChIP) experiment

ChIP experiment was performed using a ChIP Assay Kit (Beyotime, China). After stimulated by various stiffness of PA gels, CRC cells (~1 × 10^6^ cells) were used for the ChIP experiment according to the manufacturer’s protocol. Briefly, cells were incubated with formaldehyde (1%) for 10 min at 37 °C and then added with Glycine Solution (10X) for 5 min at room temperature. After washed with ice-cold PBS containing 1 mM PMSF for three times, cells were lysed by an appropriate amount of SDS Lysis Buffer containing 1 mM PMSF. After sonication, each sample was analyzed using agarose gel electrophoresis and the ideal bands were concentrated between 200 and 1000 base pairs. Immunoprecipitation was performed using the SREBP2 antibody (5 μg, Novus, USA), and IgG (5 μg, Santa, USA) acted as a control. The enrichment level of the HMGCR promoter was analyzed by qPCR using the primers listed in the Supplementary Table S1 The fold enrichment index was calculated based on the following formula: 2^^[−CT value (SREBP2 − IgG)]^.

### Animal experiments

Male C57BL/6 mice aged four-to-six weeks were purchased from Charles River (China). All animal experiments were performed in accordance with the Animal Care and Use Committee of the Capital Medical University (AEEI-2025-058). Mice were housed under specific pathogen-free conditions with 12-hour light/dark cycles and free access to water and food. Two investigators were blinded to group allocation and outcome assessment, including tumor measurement, blood collection, and tissue processing.

An orthotopic mouse model of colorectal cancer was established as follows. First, mice were anesthetized with tribromoethanol/tert-amyl alcohol (Sigma, 250 mg/kg, *i.p*.), and a ventral incision was employed to expose the cecum. Second, MC38 cells (4.0 × 10^6^), resuspended in 40 μL PBS, were injected orthotopically into the cecal wall. Two weeks after surgery, the mice were randomly assigned to treatment groups and control groups. For β-aminopropionitrile (BAPN) treatment, BAPN was given (50 mg/kg, *i.p*.) every two days, or saline was used as a control. After four-week injection, the mice were sacrificed and necropsies were performed.

An AOM/DSS model was established as described previously [37]. The model was based on a single injection with AOM (10 mg/kg, *i.p*., Aladdin) and a single cycle of 2% DSS (MeilunBio, China) in drinking water. Eight weeks after the injection, the mice were randomly assigned into three groups, including the control group, the BAPN (50 mg/kg) group and the Fatostatin (15 mg/kg) + BAPN group. Six weeks after the treatment, the mice were sacrificed and necropsies were performed.

### Human subjects

For immunohistochemistry (IHC) staining, we utilized a human CRC tissue microarray (HColA030PG08-1) sourced from Outdo Biotech Company (Shanghai, China). Additional human CRC tumor tissues were collected from patients of Beijing Friendship Hospital, with the study being approved by the Institutional Ethics Committee of Beijing Friendship Hospital (approval number 2020-P2-290-01). Informed consent was obtained from all subjects. Clinical information is presented in Supplementary Table S3.

### Bioinformatics analysis

RNA-seq data and clinical information were downloaded from The Cancer Genome Atlas (TCGA) and Gene Expression Omnibus (GEO) databases (GSE20970, GSE77955, GSE103512, GSE128449, GSE156451, GSE211831). The ECM-related genes were from the Human Gene Database (www.genecards.org). Pathway enrichment analysis were conducted based on Gene Set Enrichment Analysis (GSEA, www.gsea-msigdb.org), Gene Oncology (GO, www.geneontology.org) and Kyoto Encyclopedia of Genes and Genomes (KEGG, www.kegg.jp). Gene Regulatory Networks Database (GRNdb, www.grndb.com) and JASPAR (jaspar.genereg.net) were used to predict the transcription factors for HMGCR and the binding sites. The images of HMGCR protein level were extracted from the Human Protein Atlas (www.proteinatlas.org).

Firstly, we extracted RNA-seq data from TCGA-COAD dataset and calculated Matrix Stiffness (MS) score [38] using Gene Set Variation Analysis (GSVA) analysis. The specific genes included COL1A1, EHD2, FBLN1, FN1, LOX, MSN, PDGFRA, PDGFRB, PRRX1, RUNX1, and TNC. The detailed parameters included minimum size of gene sets = 1, maximum size of gene sets = 500, kernel function type for density estimation = Poisson, and parallel computing scale = 30. The optimal cutoff of the MS score was determined using the Youden index to dichotomize samples into high- and low-MS groups. Kaplan–Meier survival analysis was performed based on the grouping defined above, after excluding samples with survival time <30 days or missing survival data. In addition, correlations between the MS score and TNM stage as well as overall stage were analyzed. Subsequently, differentially expressed genes (DEGs) between the high- and low-MS groups were identified and then GSEA, KEGG, and GO enrichment analyses were conducted. ScRNA-seq data of four CRC datasets (GSE289314, GSE221575, GSE200997, GSE81861) were extracted and several cell types were annotated. Malignant epithelial cells were extracted and were endowed with MS score using “AddModuleScore” algorithm. Subsequently, DEGs between the high- and low-MS groups, grouped by median, were identified and then GSEA, KEGG, and GO enrichment analyses were conducted.

### Statistical analysis

All statistical analyses were performed using Rstudio (4.3.1) and GraphPad Prism 9.0.0. All the data are expressed as mean ± SD. Two-group comparisons employed either paired/unpaired *t*-tests (for normally distributed data) or Mann–Whitney U tests (for non-parametric data). Multi-group analyses utilized Kruskal–Wallis test followed by Dunn’s post-hoc correction for pairwise contrasts. For two-factor experimental designs, two-way ANOVA was performed with Tukey’s multiple comparison procedure. Quantitative colocalization analysis was performed using ImageJ software with the Colocalization Finder plugin and results were presented as Pearson’s coefficient. For survival analysis, KM survival curves and log-rank test were used. All experiments were performed with a minimum of three independent biological replicates (*n* = 3) per experimental group. Statistical significance was considered at a two-tail *p* < 0.05 (*p < 0.05, **p < 0.01, ***p < 0.001, ****p < 0.0001).

## Results

### Mechanical stiffening induces EMT and poor prognosis in CRC

**Fig. 1:**
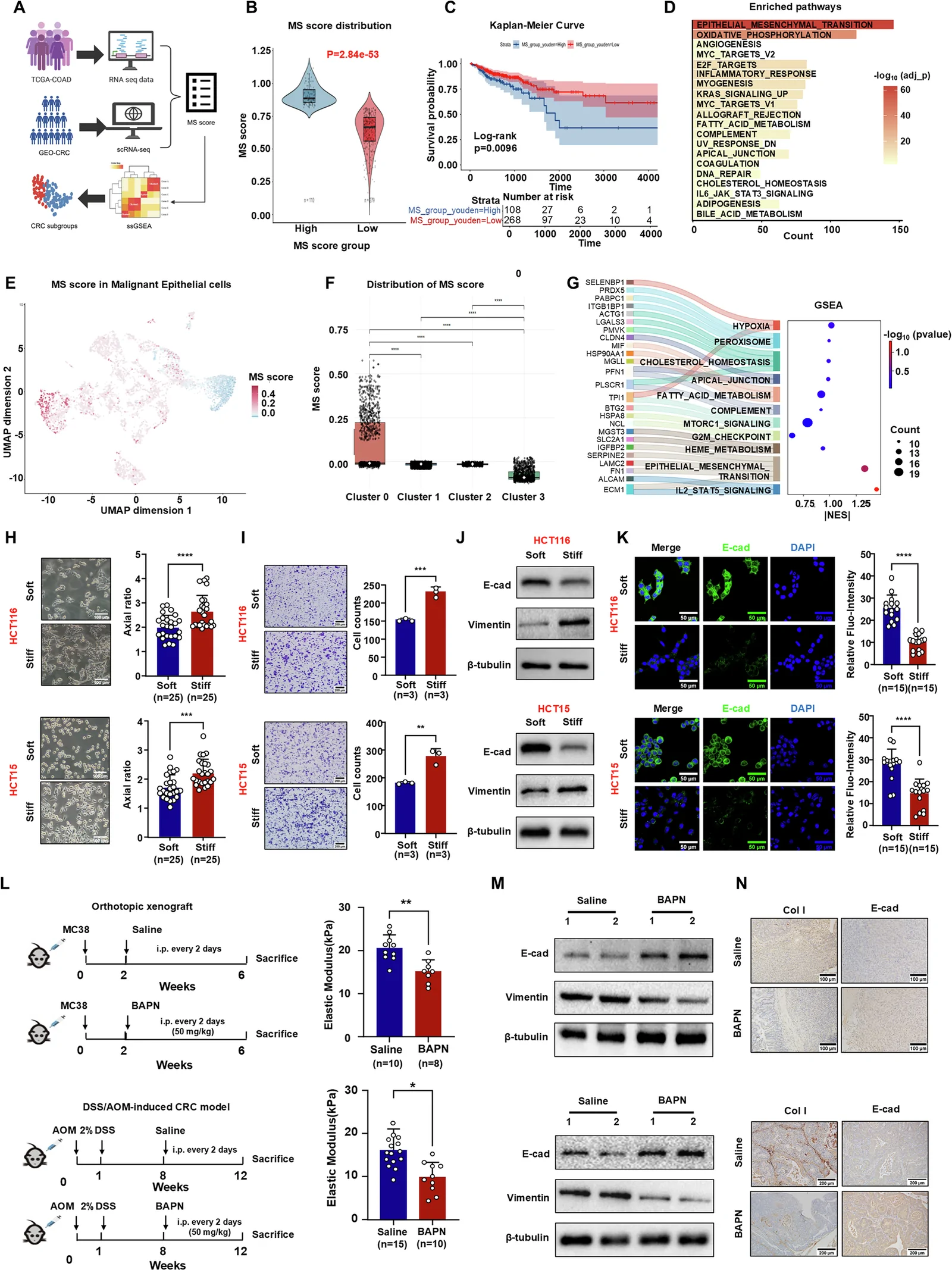
Matrix stiffness promotes EMT and poor prognosis in CRC. **A** The flowchart of bioinformatic analysis. **B** The histogram of MS scores in two groups of TCGA-COAD population. **C** The K-M survival analysis of the two groups in overall survival. **D** Gene Set Enrichment Analysis of differential genes of the two groups. **E** The distribution plot of MS score in the four clusters of malignant epithelial cells. **F** The histogram of MS score in the four clusters of malignant epithelial cells. **G** Gene Set Enrichment Analysis of differential genes of the clsuter0 and cluster3. **H** The morphology plots of HCT116 and HCT15 cells cultured on different stiffness of hydrogels. **I** The representative Transwell images of HCT116 and HCT15 cells cultured on different stiffness of hydrogels. **J** The Western blots of E-cadherin and Vimentin in HCT116 and HCT15 cells cultured on different stiffness of hydrogels. **K** The immunofluorescence staining images of E-cadherin in HCT116 and HCT15 cells cultured on different stiffness of hydrogels. **L** The schematic diagram of orthotopic xenograft and DSS/AOM induced-CRC models treated with BAPN (50 mg/kg). **M** The Western blots of E-cadherin and Vimentin. **N** The representative IHC images of collagen I and E-cadherin. Note: two-tailed *p* < 0.05 (*p < 0.05, **p < 0.01, ***p < 0.001, ****p < 0.0001) by the Mann–Whitney U test (**B**), Log-rank test (**C**), Kruskal–Wallis test with Dunn’s post-hoc correction (**F**), unpaired *t* test (**H**, **K**, **L**) or paired t test (**I**); MS matrix stiffness, E-cad E-cadherin, Col I collagen I, BAPN β-aminopropionitrile; data are presented as mean ± SD.

To quantify tissue stiffness in tumor samples from TCGA-COAD, we used the MS score, where a higher score indicates greater stiffness [38], to roughly estimate matrix stiffness for each sample. We then stratified 389 tumors into two distinct subtypes, “Soft” and “Stiff”, after ruling out the samples with missing data based on the Youden index (Fig. 1A, Supplementary Fig. S1A), and the Stiff group exhibited a significantly higher MS score (Fig. 1B). Kaplan–Meier survival analysis revealed significantly poorer overall survival in the Stiff group (*p* < 0.01; Fig. 1C). To explore the biological behaviors associated with these subtypes, we performed functional enrichment analysis of DEGs and found significant enrichment in pathways related to EMT and cholesterol metabolism (Fig. 1D). To further clarify the impact of matrix stiffness on tumor cells, we integrated four CRC single-cell transcriptome datasets and obtained 60,929 high-quality cells using the Seurat integration algorithm. All cells were classified into seven major cell types based on canonical marker genes (Supplementary Fig. S1B, C). Malignant cells were subsequently reclustered and divided into four primary subclusters (Supplementary Fig. S1D, E). MS score-based visualization of matrix stiffness status in malignant cells showed that cells with high ECM stiffness predominantly clustered in Cluster 0, whereas Cluster 3 exhibited lower stiffness (Fig. 1E, F). GSEA indicated that ECM stiffening regulated EMT in malignant cells (Fig. 1G).

In vitro, CRC cells cultured on stiff polyacrylamide hydrogels displayed an elongated morphology with increased axial ratio and spreading area (Fig. 1H and Supplementary Fig. S2A, B), along with enhanced migration (Fig. 1I). Consistently, stiff substrates upregulated the mesenchymal marker Vimentin and downregulated the epithelial marker E-cadherin (Fig. 1J, K and Supplementary Fig. S2C, D). In vivo, BAPN treatment reduced tumor stiffness in both orthotopic xenograft and DSS/AOM-induced CRC models (Fig. 1L and Supplementary Fig. S2E), and this reduction correlated with elevated E-cadherin and suppressed Vimentin expression (Fig. 1M, N). These results establish that matrix stiffening promotes EMT and a pro-invasive phenotype in CRC.

### Matrix stiffening reprograms cholesterol metabolism to drive EMT

**Fig. 2:**
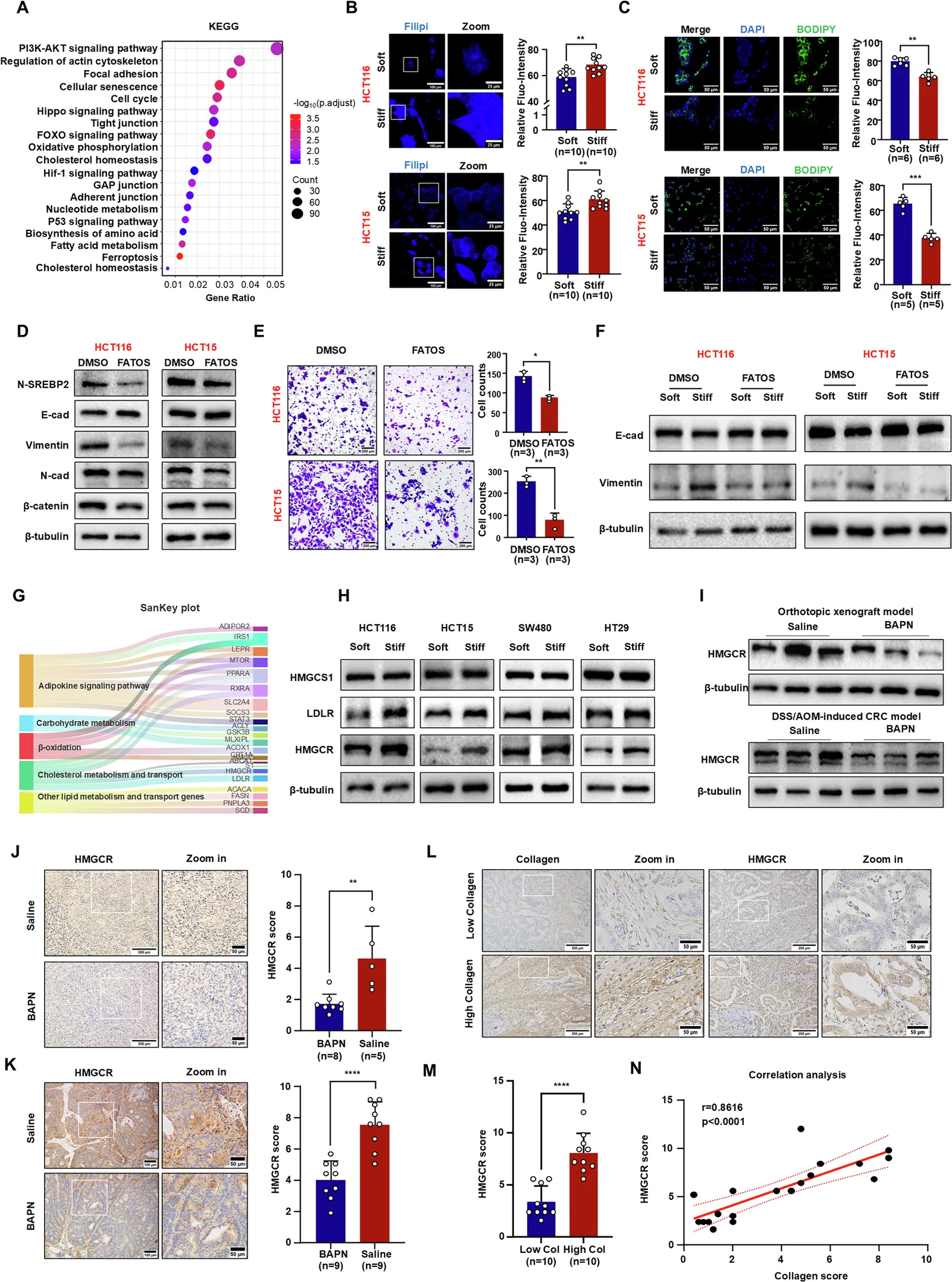
Matrix stiffening reprograms cholesterol metabolism to drive EMT. **A**. KEGG analysis of RNA-seq data in HCT116 cells. The free cholesterol levels (**B**) and lipid droplets (**C**) in HCT116 and HCT15 cells were cultured on different stiffness of hydrogels. The Western blots (**D**) of E-cadherin, Vimentin, N-cadherin, β-catenin, and the Transwell images (**E**) in HCT116 and HCT15 treated with Fatostatin (10 μM). **F** The Western blots of E-cadherin and Vimentin in HCT116 and HCT15 cultured on different stiffness of PA hydrogels and were then treated with Fatostatin (10 μM). **G** The SanKey plot of lipid-metabolism-related genes in HCT116 cells cultured on different stiffness of PA gels. **H** The Western blots of HMGCS1, LDLR, and HMGCR in different CRC cells (HCT116, HCT15, SW480, and HT29) cultured on different stiffness of hydrogels. The Western blots (**I**) and representative IHC images (**J**, **K**) of HMGCR in the orthotopic xenograft of CRC mice model and the AOM/DSS mice model. The representative IHC images (**L**), relative quantitative analysis (**M**), and the correlation analysis (**N**) of collagen I and HMGCR in CRC tissues in our cohort (*n* = 20). Note: two-tailed *p* < 0.05 (*p < 0.05, **p < 0.01, ***p < 0.001, ****p < 0.0001) by the unpaired *t* test (**B**, **C**, **J**, **K**, **M**), paired *t* test (**E**) or simple linear regression (**N**); FATOS, Fatostatin; data are presented as mean ± SD.

GSEA indicated that the mechanical signals from ECM stiffening also disrupted cholesterol homeostasis in CRC (Fig. 1D, G). RNA sequencing of CRC cells cultured on stiff substrates identified DEGs (Supplementary Fig. S3A and Supplementary Table S4) that were significantly enriched in fatty acid and cholesterol homeostasis pathways (Fig. 2A). In the TCGA database, enrichment scores and gene expression levels of the cholesterol homeostasis pathway were significantly correlated with those of the EMT pathway (Supplementary Fig. S3B, C). Functionally, matrix stiffening increased cellular total and free cholesterol levels and reduced lipid droplet content in vitro (Fig. 2B, C and Supplementary Fig. S4A), whereas in vivo matrix softening by BAPN lowered serum cholesterol in both orthotopic and DSS/AOM-induced models (Supplementary Fig. S4B). Pharmacological inhibition of cholesterol synthesis with Fatostatin reduced cellular cholesterol (Supplementary Fig. S4C), downregulated mesenchymal markers (Vimentin, N-cadherin) and β-catenin, restored E-cadherin expression (Fig. 2D), and significantly suppressed cell invasion (Fig. 2E). Fatostatin also lowered cellular cholesterol across different stiffness conditions and abolished stiffness-induced E-cadherin suppression and Vimentin induction (Fig. 2F and Supplementary Fig. S4D). These findings implicate reprogramming of cholesterol metabolism as a critical mediator of stiffness-induced EMT.

### Stiffness-induced HMGCR upregulation links ECM mechanics to cholesterol metabolism

To identify molecular mediators linking substrate stiffness to cholesterol metabolism, we performed PCR microarray screening and identified HMGCR, LDLR, and HMGCS1 as stiffness-responsive genes (Fig. 2G). Validation across multiple CRC cell lines confirmed that HMGCR expression was consistently and robustly upregulated by stiffening (Fig. 2H and Supplementary Fig. S5A). In vivo, BAPN-induced matrix softening reduced HMGCR expression in both orthotopic xenografts and DSS/AOM-induced CRC tissues (Fig. 2I–K and Supplementary Fig. S5B). In a clinical cohort (*n* = 20), IHC revealed a positive correlation between HMGCR and collagen I levels (Fig. 2L–N), supporting the clinical relevance of the stiffness-HMGCR axis.

### HMGCR upregulation promotes CRC progression

**Fig. 3:**
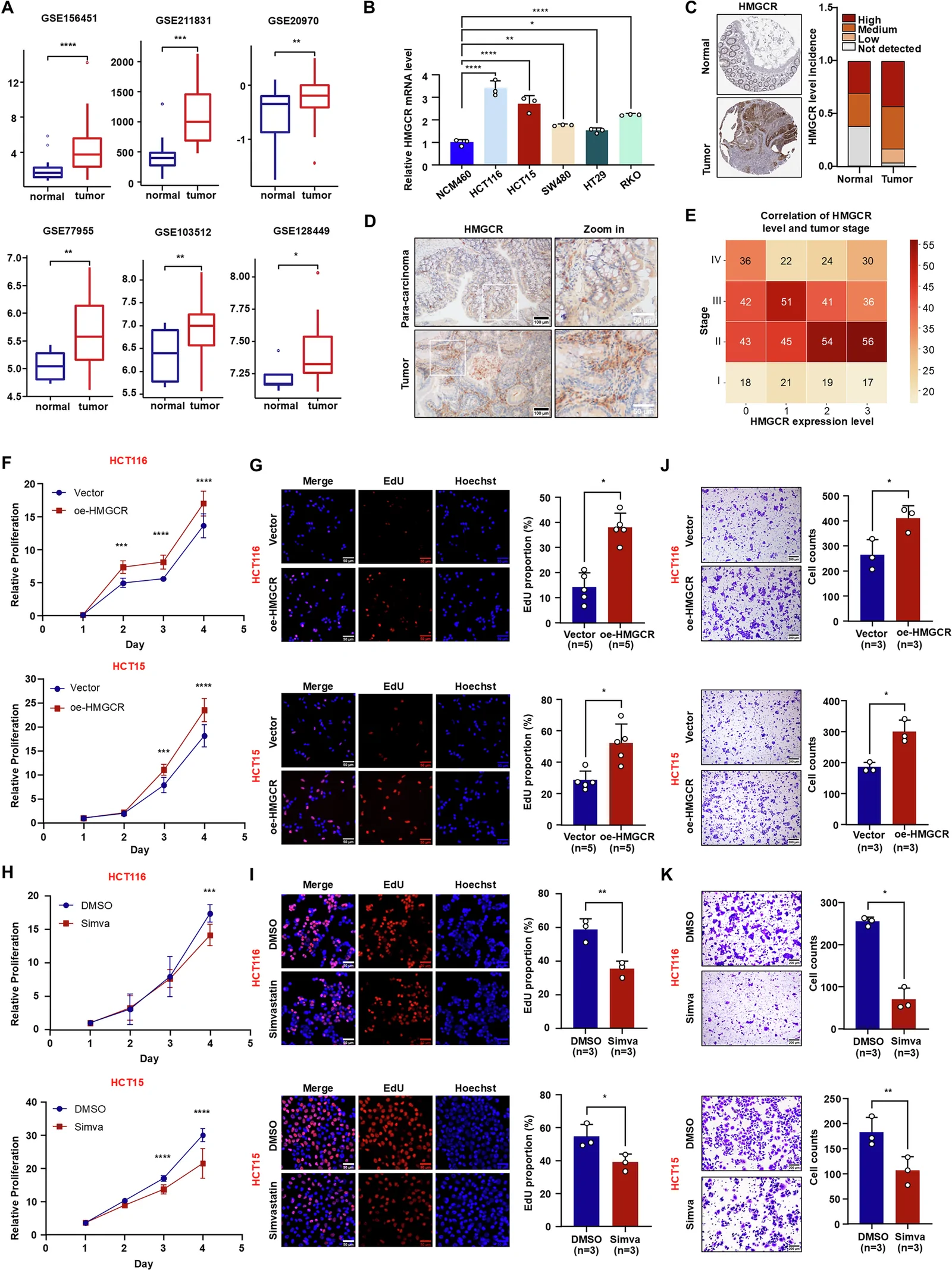
HMGCR upregulation promotes CRC progression. **A** The HMGCR expression level of CRC and normal tissues from six different GEO databases. **B** The mRNA level of HMGCR in the normal enterocyte (NCM460) and different CRC cells (HCT116, HCT15, SW480, HT29, and RKO). **C** The immunostaining analysis of HMGCR in primary CRC tissues from the HPA database. **D** The representative immunohistochemical images of HMGCR of tumor and para-carcinoma tissues from the AOM/DSS model. **E** The correlation analysis of HMGCR expression and tumor stages from TCGA-COAD population. **F**, **G** HCT116 and HCT15 cells were transfected with the HMGCR plasmid. The cells were harvested for CCK8 (**F**), and EdU (**G**). **H**, **I** The HCT116 and HCT15 cells were treated with the inhibitor of HMGCR, namely Simvastatin (10 μM). The cells were harvested for CCK8 (**F**), and EdU (**G**). The Transwell tests of HCT116 and HCT15 cells which were transfected with the HMGCR plasmid (**J**) and inhibited with Simvastatin (**K**). Note: two-tailed *p* < 0.05 (*p < 0.05, **p < 0.01, ***p < 0.001, ****p < 0.0001) by the Mann–Whitney U test (**A**), one-way ANOVA followed by Dunnett’s post hoc test (**B**), two-way ANOVA test (**F**, **H**), unpaired *t* test (**G**, **I**), or paired *t* test (**J**, **K**); Simva, Simvastatin; data are presented as mean ± SD.

Analysis of public databases and our own data confirmed elevated HMGCR expression in CRC tissues and cell lines compared with normal counterparts (Fig. 3A–D and Supplementary Fig. S6). Integrated analysis of TCGA and GEO data revealed a significant positive correlation between HMGCR expression levels and advancing tumor stage (Fig. 3E). Functionally, HMGCR overexpression enhanced cholesterol synthesis (Supplementary Fig. S7A, B) and promoted CRC cell proliferation (Fig. 3F, G) and invasion (Fig. 3J). Conversely, HMGCR inhibition with Simvastatin or siRNA-mediated knockdown (Supplementary Fig. S7C, D) reduced cholesterol levels (Supplementary Fig. S7E, F), suppressed proliferation (Fig. 3H, I and Supplementary Fig. S7G, H), and attenuated invasive capacity (Fig. 3K and Supplementary Fig. S7I). These data establish HMGCR as a key driver of CRC progression.

### HMGCR mediates stiffness-induced EMT by disrupting β-catenin/E-cadherin complexes

**Fig. 4:**
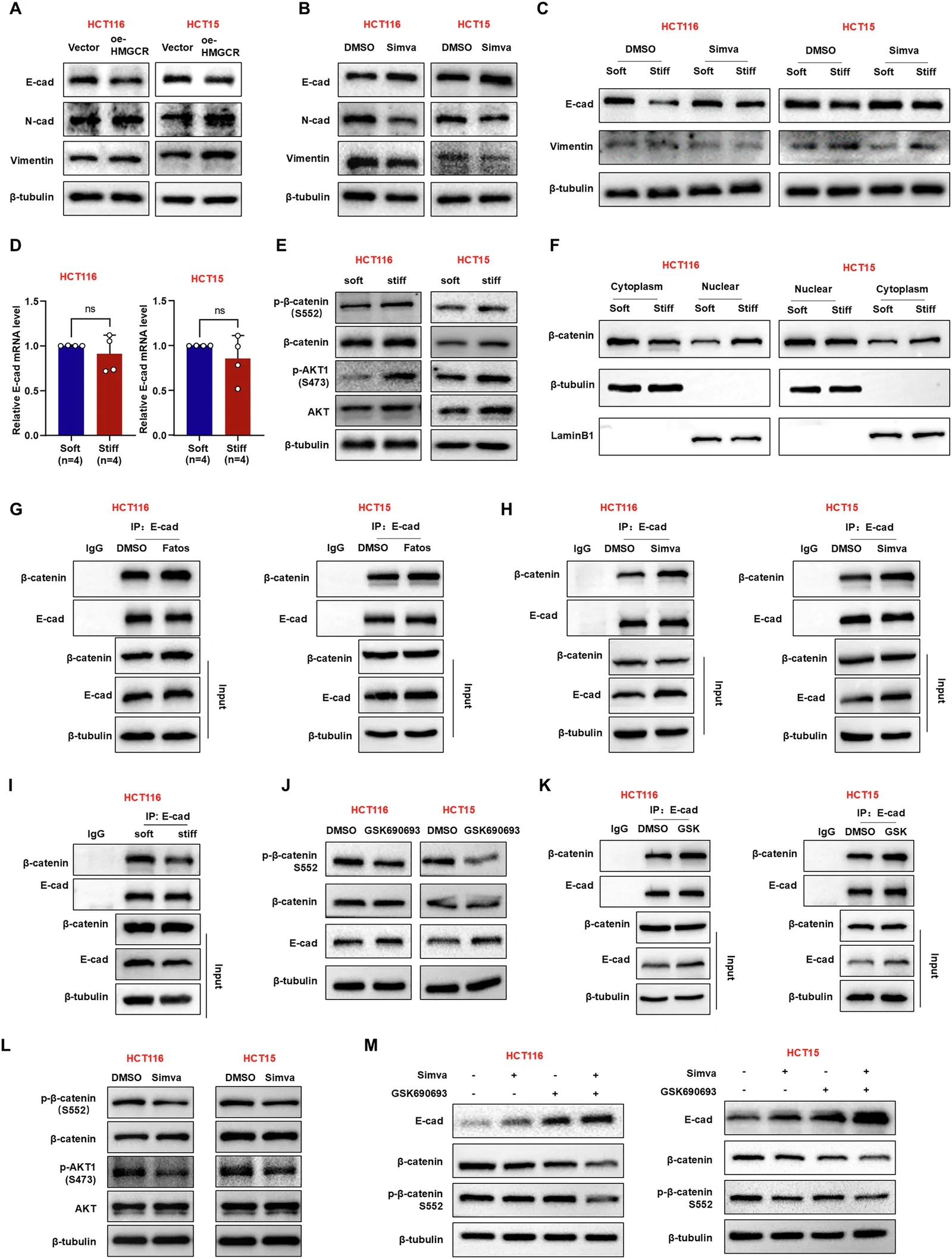
HMGCR drives matrix stiffness-induced EMT and tumorigenicity by disrupting β-catenin/E-cadherin complexes. The Western blots of E-cadherin, N-cadherin and Vimentin in HCT116 and HCT15 cells which were transfected with the HMGCR plasmid (**A**) and were inhibited with Simvastatin (**B**). **C** The Western blots of E-cadherin and Vimentin in HCT116 and HCT15 cells cultured on the different stiffness of PA hydrogels were treated with Simvastatin (10 μM). **D** The mRNA level of E-cadherin of HCT116 and HCT15 cells cultured on the different stiffness of PA hydrogels. **E** The Western blots of phosphorylation of β-catenin at Ser552, β-catenin, phosphorylation of AKT at Ser473 and AKT in HCT116 and HCT15 cultured on different stiffness of PA hydrogels. **F** The nuclear cytoplasm separation analysis of β-catenin in HCT116 and HCT15 cultured on different stiffness of PA hydrogels. The CO-IP analysis of HCT116 and HCT15 treated with Fatostatin (**G**) and Simvastatin (**H**). **I** The CO-IP analysis of HCT116 cultured on the different stiffness of PA hydrogels. **J** The Western blots of phosphorylation of β-catenin at Ser552, β-catenin and E-cadherin in HCT116 and HCT15 cells treated with GSK690693. **K** The CO-IP analysis of HCT116 and HCT15 treated with GSK690693. The Western blots of HCT116 and HCT15 cells treated with Simvastatin (**L**) and combination of Simvastatin and GSK690693 (**M**). Note: two-tailed *p* < 0.05 (*p < 0.05, **p < 0.01, ***p < 0.001, ****p < 0.0001) by the *p*aired *t* test (**D**); E-cad, E-cadherin, N-cad, N-cadherin, Simva, Simvastatin, Fatos, Fatostatin, GSK, GSK690693, ns, not significant; data are presented as mean ± SD.

To clarify the role of HMGCR in stiffness-induced EMT, we examined its specific function. HMGCR overexpression suppressed E-cadherin and induced N-cadherin and Vimentin (Fig. 4A), whereas HMGCR inhibition with Simvastatin produced the opposite effects (Fig. 4B). Simvastatin also partially reversed stiffening-induced cholesterol elevation (Supplementary Fig. S8) and rescued the associated E-cadherin downregulation and Vimentin upregulation (Fig. 4C), confirming the functional involvement of HMGCR. Notably, matrix stiffness did not alter CDH1 (E-cadherin) mRNA levels (Fig. 4D), indicating that E-cadherin downregulation occurs post-transcriptionally. This downregulation was accompanied by increased phosphorylation of β-catenin at Ser552 (Fig. 4E) and subsequent nuclear accumulation of β-catenin (Fig. 4F). Since dissociation of β-catenin from E-cadherin is a critical step in adhesion junction disassembly, we examined their interaction. Co-immunoprecipitation assays showed that treatment with Fatostatin or Simvastatin, both of which inhibit cholesterol synthesis, significantly enhanced β-catenin/E-cadherin binding (Fig. 4G, H). Contrastly, matrix stiffening markedly reduced this interaction (Fig. 4I). Crucially, direct inhibition of β-catenin phosphorylation restored E-cadherin expression (Fig. 4J) and significantly strengthened β-catenin/E-cadherin binding (Fig. 4K). Finally, Simvastatin treatment effectively suppressed stiffness-induced β-catenin phosphorylation (Fig. 4L) and reversed the corresponding E-cadherin downregulation (Fig. 4M). Together, these data delineate a pathway in which matrix stiffness, through HMGCR, promotes β-catenin phosphorylation and its dissociation from E-cadherin, leading to junctional disassembly and EMT.

### Matrix stiffening transcriptionally promotes HMGCR via SREBP2 activation

**Fig. 5:**
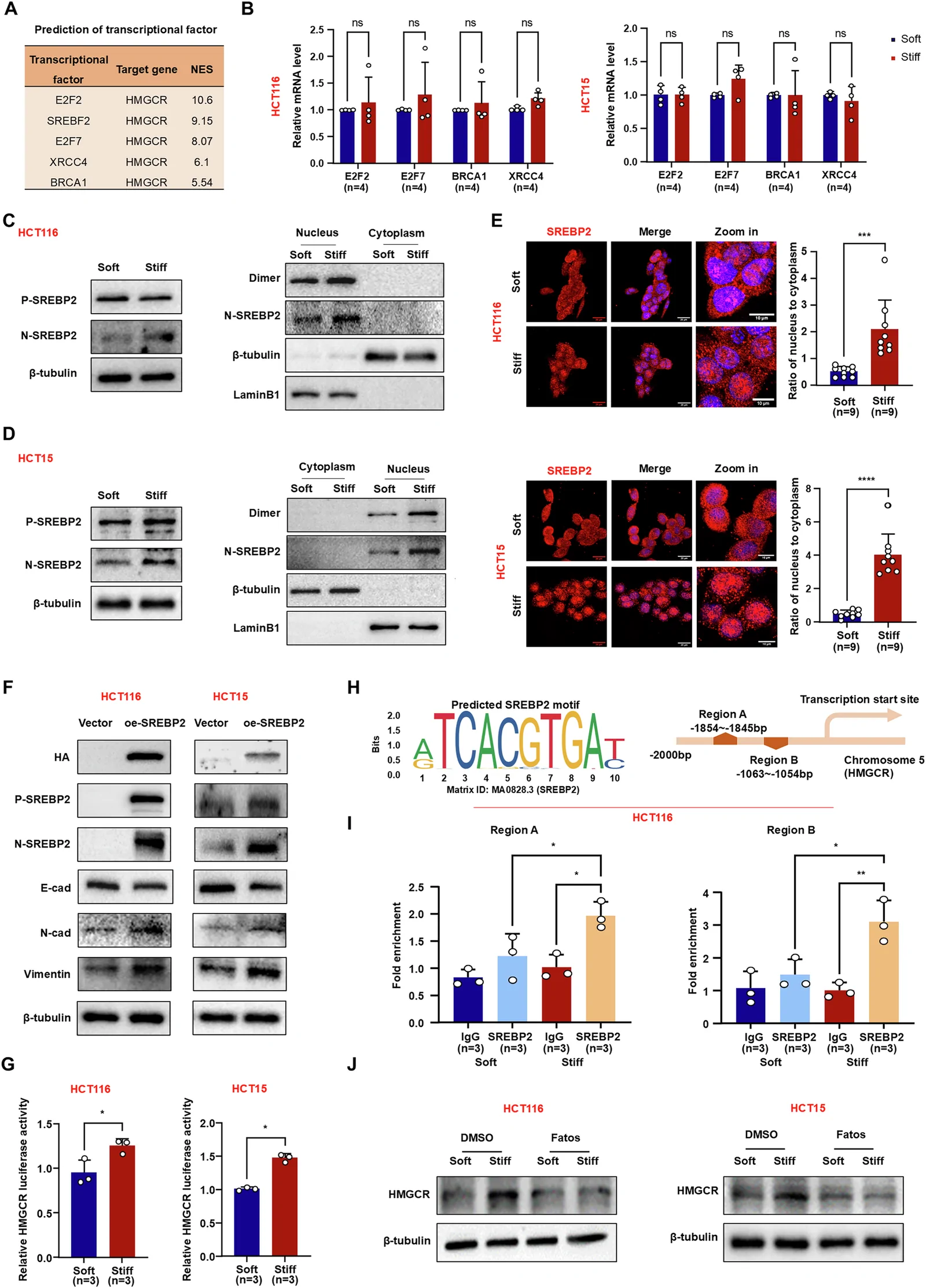
Matrix stiffness-induced HMGCR activation were transcriptionally regulated by SREBP2. **A** The top 5 predicted transcriptional factors of HMGCR from the GRNdb. **B** The mRNA levels of the predicted transcriptional factors in the HCT116 and HCT15 cultured on different stiffness of PA hydrogels. The nuclear cytoplasm separation analysis of HCT116 (**C**) and HCT15 (**D**) cultured on different stiffness of PA hydrogels. **E** The representative immunofluorescence images of SREBP2 in the HCT116 and HCT15 cultured on different stiffness of PA hydrogels. **F** The HCT116 and HCT15 cells transfected with the SREBP2 plasmid were harvested for Western blot analysis. **G** The luciferase activity of HMGCR in the HCT116 and HCT15 cultured on different stiffness of PA hydrogels. **H** The predicted binding sites of HMGCR promoter according to the JASPAR database. **I** The ChIP-PCR tests of the HCT116 and HCT15 cultured on different stiffness of PA hydrogels. **J** The Western blot analysis of HCT116 and HCT15 cells cultured on different stiffness of PA hydrogels and then treated with Fatostatin (10 μM). Note: two-tailed *p* < 0.05 (*p < 0.05, **p < 0.01, ***p < 0.001, ****p < 0.0001) by the *p*aired *t* test (**B**, **G**), un*p*aired *t* test (**E**), or one-way ANOVA followed by Turkey’s test (**I**); E-cad E-cadherin, N-cad N-cadherin, Fatos Fatostatin, ns not significant; data are presented as mean ± SD.

To elucidate how stiffness upregulates HMGCR, we analyzed potential transcription factors using GRNdb. SREBF2 (SREBP2) was among the top predicted regulators (Fig. 5A). While SREBF2 mRNA levels remained unchanged (Fig. 5B and Supplementary Fig. S9), the transcriptionally active cleaved form of SREBP2 (50–75 kDa) and its dimeric precursor (120–150 kDa) were increased in nuclear fractions under stiff conditions (Fig. 5C, D). Immunofluorescence confirmed stiffness-induced nuclear translocation of SREBP2 (Fig. 5E). Functionally, SREBP2 overexpression promoted EMT (Fig. 5F). Luciferase reporter and ChIP-qPCR assays demonstrated that stiff matrix enhanced both HMGCR promoter activity and SREBP2 binding to this promoter (Fig. 5G–I). Moreover, the SREBP inhibitor Fatostatin blocked stiffness-induced HMGCR upregulation (Fig. 5J). These data identify SREBP2 as the key transcriptional activator responsible for HMGCR induction in response to matrix stiffening.

### Stiffness promotes SREBP2 activation via induction of Golgi–ER fusion

**Fig. 6:**
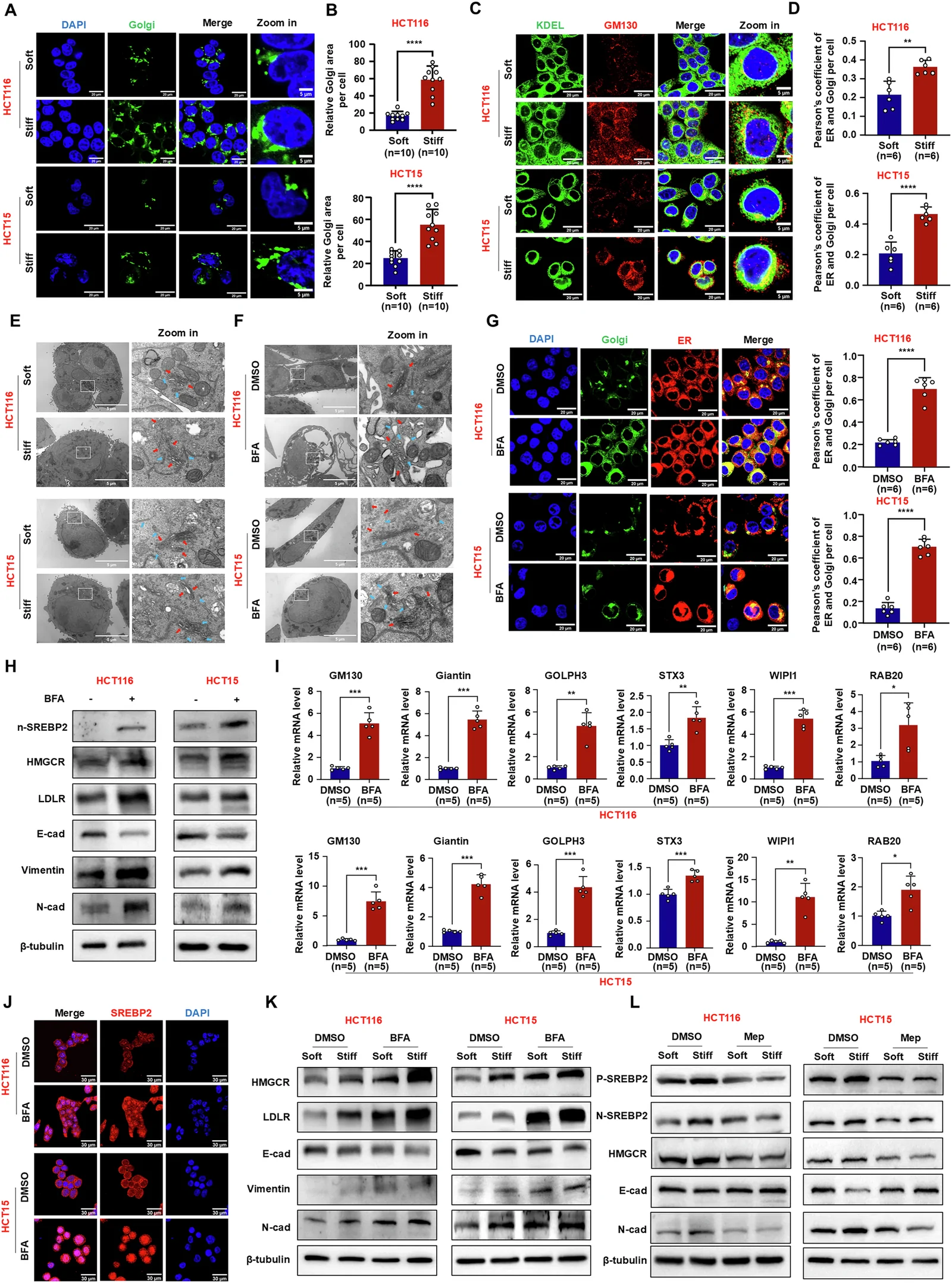
Matrix stiffness-induced Golgi–ER fusion regulates the SREBP2/HMGCR pathway. The immunofluorescence staining (**A**) and quantitative morphometric analysis (**B**) of Golgi apparatus in the living HCT116 and HCT15 cultured on different stiffness of PA hydrogels. The colocalization analysis of ER and Golgi using immunofluorescence staining (**C**) and quantitative analysis (**D**) in the HCT116 and HCT15 cultured on different stiffness of PA hydrogels. The transmission electron microscopy images of ER and Golgi in HCT116 and HCT15 under different stiffness (**E**) and treated with BFA (**F**). **G** The immunofluorescence staining of ER and Golgi in the living HCT116 and HCT15 cells treated by BFA (50 μM, 2 h). **H** The Western blot analysis of cholesterol biosynthesis-related genes and EMT-related genes. **I** The mRNA levels of Golgi-related genes in HCT116 and HCT15 treated by BFA (200 nM, 24 h). **J** The immunofluorescence staining of SREBP2 in HCT116 and HCT15 treated by BFA (200 nM, 24 h). **K**, **L** The Western blot analysis of cholesterol biosynthesis-related and EMT-related proteins in the HCT116 and HCT15 under different stiffness treated by BFA (200 nM, 24 h) and Mep (40 μg/ml, 24 h). Note: two-tailed *p* < 0.05 (*p < 0.05, **p < 0.01, ***p < 0.001, ****p < 0.0001) by the *p*aired *t* test (**I**), unpaired *t* test (**B**, **D**, **G**); ER endoplasmic reticulum, E-cad E-cadherin, N-cad N-cadherin, BFA Brefeldin A, Mep Mepanipyrim; data are presented as mean ± SD.

We next explored how stiffness regulates SREBP2 processing. Although no changes were observed in the mRNA or total protein levels of core SREBP2 pathway components (SCAP, Insig1, S1P, S2P) (Supplementary Fig. S10), IF staining revealed that stiffening induced a dramatic reorganization of the Golgi apparatus. Golgi apparatus became dispersal (Fig. 6A). Morphometric analysis confirmed a significant increase in Golgi area per cell on stiff substrates (Fig. 6B). Consistent with structural remodeling, expression of Golgi structural (GM130, GOLPH3, Giantin) and trafficking proteins (STX3, WIPI1, RAB20) was elevated (Supplementary Fig. S11). Importantly, stiff matrix robustly stimulated Golgi apparatus remodeling, including cisternal destruction and dilation, and structural disorder. Stiff matrix also promotes the proximity and fusion of ER-Golgi, as evidenced by increased colocalization (Fig. 6C–E). To test whether Golgi-ER fusion is sufficient for SREBP2 activation, we used brefeldin A (BFA), a known inducer of Golgi-ER fusion. BFA treatment recapitulated the stiffness-induced fusion phenotype (Fig. 6F, G and Supplementary Fig. S12), upregulated Golgi-related genes, and enhanced SREBP2 cleavage, nuclear translocation, and target gene transcription (Fig. 6H–J and Supplementary Fig. S13). Notably, BFA synergized with matrix stiffening to amplify cholesterol biosynthesis and EMT (Fig. 6K), while treating Mepanipyrim which suppressed Golgi-ER fusion could reverse stiffness-activated cholesterol biosynthesis and EMT (Fig. 6L). These findings demonstrate that matrix stiffness drives Golgi-ER fusion, which facilitates the proteolytic activation and nuclear entry of SREBP2.

### Stiffness promotes Golgi–ER fusion by activating Piezo1

**Fig. 7:**
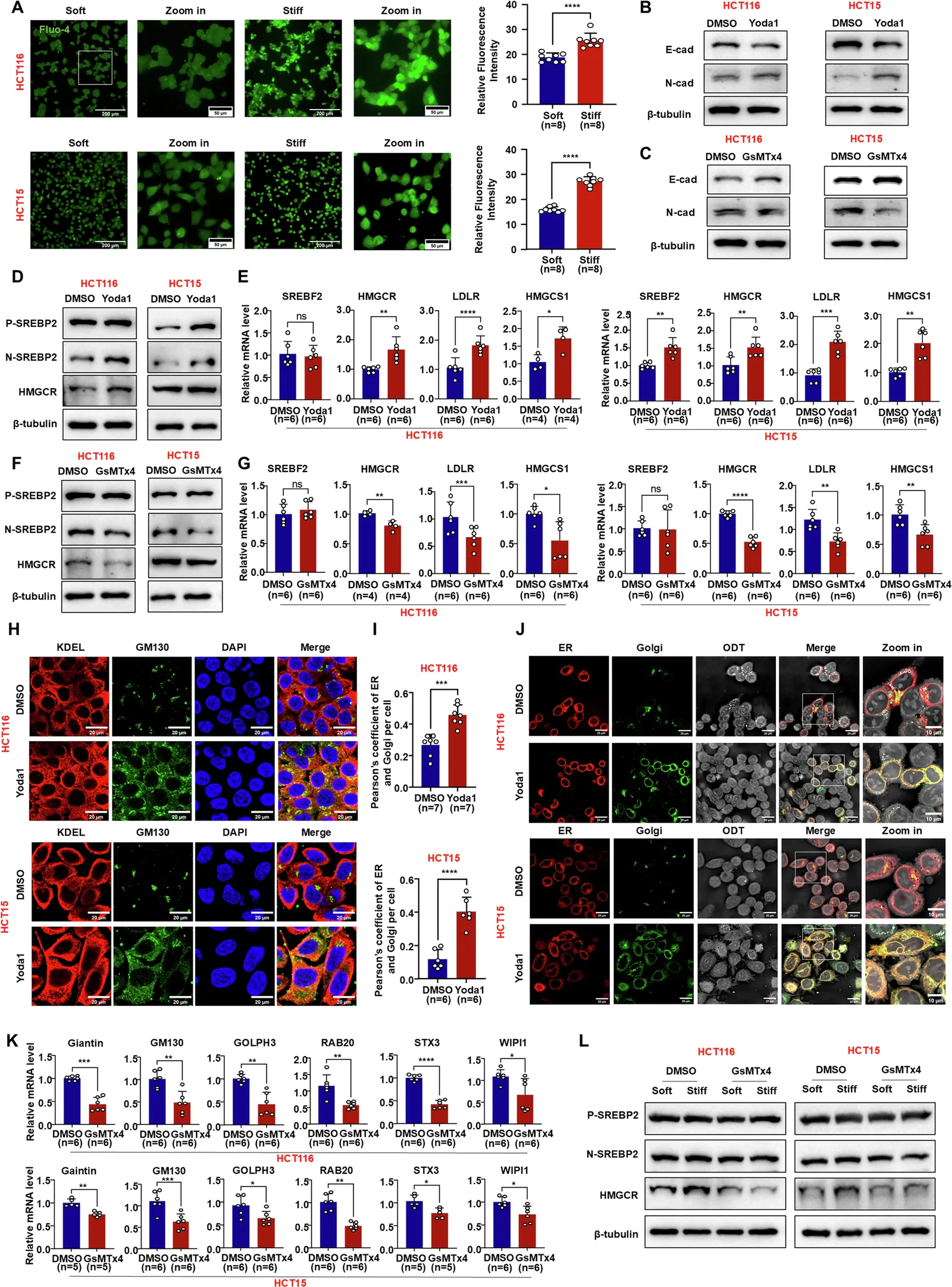
Matrix stiffness promotes Golgi–ER fusion by activating Piezo1. **A** The representative images (Left) and relative quantitative analysis (Right) of calcium concentration in HCT116 and HCT15 under different stiffness. The Western blots of E-cadherin and N-cadherin in HCT116 and HCT15 treated with Yoda1 (10 μm, **B**) and GsMTx4 (2.5 μm, **C**). The Western blots and mRNA levels of SREBP2 and target genes in HCT116 and HCT15 treated with Yoda1 (10 μm, **D**, **E**) and GsMTx4 (2.5 μm, **F**, **G**). The representative confocal images (**H**) of ER (KDEL) and Golgi (GM130) and relative quantitative analysis (**I**) in HCT116 and HCT15 treated with Yoda1 (10 μm). **J** The representative ODT images of ER and Golgi in HCT116 and HCT15 treated with Yoda1 (10 μm). The mRNA levels (**K**) of Golgi-related genes in HCT116 and HCT15 treated with GsMTx4 (2.5 μm) and Western blots (**L**) of SREBP2 and HMGCR in in HCT116 and HCT15 under different stiffness treated with GsMTx4 (2.5 μm). Note: two-tailed *p* < 0.05 (*p < 0.05, **p < 0.01, ***p < 0.001, ****p < 0.0001) by the paired t test (**E**, **G**, **K**), unpaired *t* test (**A**, **I**); ER endoplasmic reticulum, ns not significant; data are presented as mean ± SD.

Piezo1 is a mechanosensitive Ca²⁺ channel that mediates mechanical responses in CRC cells. We therefore examined the effect of substrate stiffness on calcium influx. Stiff substrates increased calcium influx in CRC cells (Fig. 7A). Activation of Piezo1 with Yoda1 decreased E-cadherin, increased N-cadherin, and promoted EMT (Fig. 7B), whereas inhibition of Piezo1 with GsMTx4 suppressed EMT (Fig. 7C). Yoda1-induced Piezo1 activation increased the levels of the transcriptionally active cleaved form of SREBP2 (50–75 kDa) and target gene transcription (Fig. 7D, E). Conversely, Piezo1 inhibition by GsMTx4 had the opposite effect (Fig. 7F, G). Importantly, Piezo1 activation markedly stimulated Golgi dispersal and fusion with the ER, as shown by increased colocalization (Fig. 7H–J). Consistent with this remodeling, Yoda1 upregulated Golgi structural proteins (GM130, GOLPH3, Giantin) and trafficking proteins (STX3, WIPI1, RAB20) (Supplementary Fig. S14), whereas these proteins were downregulated by GsMTx4 (Fig. 7K). Furthermore, GsMTx4-mediated suppression of Piezo1 attenuated matrix stiffening-induced SREBP2 activation and HMGCR upregulation (Fig. 7L). Collectively, matrix stiffness activates Piezo1 to drive Golgi-ER fusion and SREBP2-dependent HMGCR upregulation.

### Co-targeting ECM stiffness and cholesterol metabolism synergistically inhibits CRC progression

**Fig. 8:**
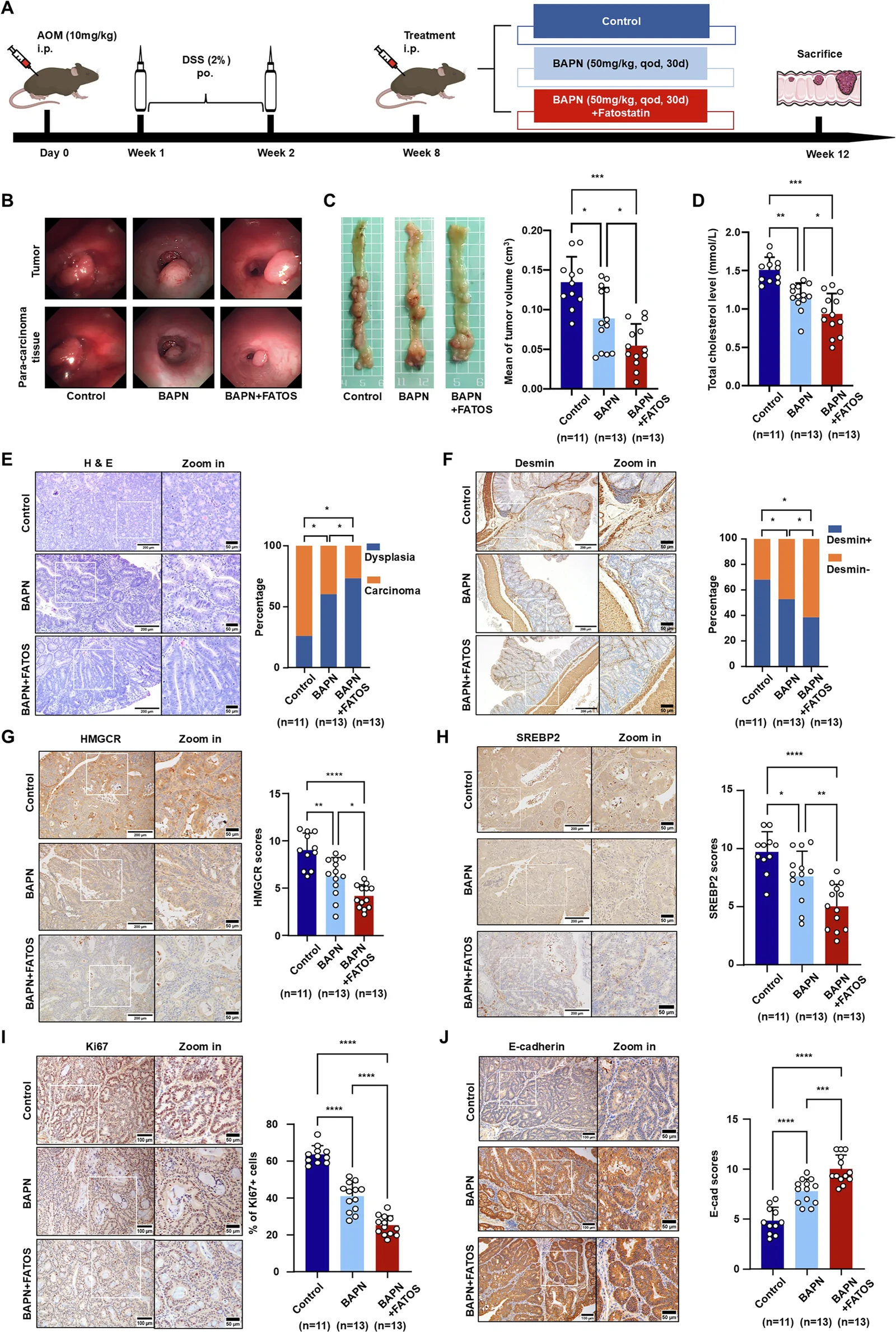
The synergistic anticancer strategy targeting matrix stiffness and cholesterol metabolism inhibits CRC progression. **A** The schematic diagram of the AOM/DSS-induced mice CRC model. The mouse colonoscopy images (**B**) and the average tumor volume (**C**) of the mice. **D** The serum cholesterol levels of the mice. The representative H&E staining (**E**) and the immunohistochemistry staining with Desmin (**F**), HMGCR (**G**), SREBP2 (**H**), Ki67 (**I**), and E-cadherin (**J**). Note: two-tailed *p* < 0.05 (*p < 0.05, **p < 0.01, ***p < 0.001, ****p < 0.0001) by the Tukey’s multiple comparisons test (**C**, **D**, **G**, **H**, **I**, **J**) or Chi-squared test (**E**, **F**); BAPN, β-aminopropionitrile, FATOS, Fatostatin; data are presented as mean ± SD.

We evaluated a combined therapeutic approach targeting ECM stiffness and cholesterol metabolism in an AOM/DSS-induced CRC model (Fig. 8A). Pre-harvest colonoscopy revealed more pronounced intraluminal hemorrhage in control mice compared with BAPN- or combination (BAPN/Fatostatin)-treated groups. Control tumors exhibited a friable texture, irregular surface, and contact-induced bleeding (Fig. 8B). Both BAPN monotherapy and combination therapy significantly reduced tumor volume and serum cholesterol levels (Fig. 8C, D). Histological analysis showed a lower proportion of carcinomas in BAPN-treated mice than in controls (Fig. 8E). Desmin staining revealed more frequent rupture of the muscularis mucosa in controls, indicating greater invasiveness, which was reduced in both treatment groups (Fig. 8F). SREBP2 and its target gene HMGCR were significantly higher in control tumors, and combination therapy further reduced both (Fig. 8G, H). The proliferation marker Ki-67 was significantly elevated in control tumors, and combination therapy further decreased the number of Ki-67+ cells (Fig. 8I). BAPN treatment restored E-cadherin expression, an effect enhanced by combination therapy (Fig. 8J). These results demonstrate that ECM modulation by BAPN, alone or in combination with cholesterol synthesis inhibition, effectively suppresses CRC progression by reducing tumor burden, invasion, and proliferation while promoting epithelial integrity.

## Discussion

All cells experience force and all cells need energy to respond to their environments [39]. Especially, tumor cells have to produce much energy for rapid proliferation, self-renewal and differentiation. They also need to overcome surrounding obstruction for protrusion and invasion that requires enough energy. Therefore, understanding the mutual transmission between physical stimuli and biochemical signaling has great potential to discover new therapeutic targets. In this study, we focus on the two major issues of the physical stimuli and metabolic rewriting in CRC and innovatively propose that SREBP2 coordinates matrix stiffness with the cholesterol homeostasis to promote EMT.

Dysregulated cholesterol homeostasis has been proved to be a hallmark of CRC, especially enhanced cholesterol biosynthesis [40]. This study found increased cholesterol levels in the CRC cells cultured on stiffer substrates and decreased serum cholesterol concentration in the mice treated with BAPN. We found HMGCR might be the most critical gene of the cholesterol biosynthesis pathway when responded to matrix stiffness. Overexpression of HMGCR could promote CRC cells proliferation, invasion and EMT. The specific blockage of HMGCR activity could reverse these oncogenic effects meanwhile partly reverse matrix stiffness-induced CRC progression. Consistent with our results, increasing evidence has discovered synergetic anti-cancer effects of anti-HMGCR drugs such as statins [41]. The potential mechanisms of statins focus on the modulation of cell proliferation, differentiation, and apoptosis through the inhibition of posttranslational modification and activation of small GTPases and their subsequent intracellular signaling. In addition, statins may inhibit EMT through the depletion of metabolic products which play a crucial role in cell polarity, motility, adhesion, membrane integrity and cytoskeletal function. However, clinical and preclinical studies on statins’ anti-cancer efficacy report conflicting outcomes. For example, A 2025 meta-analysis of 46,154 patients across 25 studies found a 20% mortality reduction with statin use. However, survival benefits were confined to European populations, with no significant effects in Asian or U.S. cohorts, which raised concerns about regional heterogeneity and unaccounted confounders like dosage and adherence [42]. Despite promising preclinical data, systematic reviews have failed to confirm consistent anticancer efficacy in prospective trials, and some studies even link statins to poorer outcomes in certain cancers or increased toxicity [43, 44]. Biomarker analyses further complicate the picture: high tumor HMGCR expression correlates with lethal prostate cancer risk, yet statin use does not appear to alter its expression, suggesting that intrinsic metabolic reprogramming may limit therapeutic impact [45]. Therefore, the uncertain effects of statins are potentially due to variations in cancer types, statin regimens, and treatment duration that are necessary factors in the design of future researches.

Previous studies have shown that SREBP2 is a classical transcriptional factor that regulates the expression of key enzymes in the cholesterol biosynthesis pathway, including HMGCR [46]. Our results also showed matrix stiffness could stimulate SREBP2 to bind to the promoter of HMGCR to increase its transcriptional level. In addition, the activation of SREBP2 could increase the cholesterol level of CRC cells and promote invasion and migration. In vivo, specific inhibition of SREBP2 using Fatostatin could effectively reduce tumor volume and delay tumor progression. These multiple lines of evidence support the cancer-promoting effects of SREBP2 in CRC, consistent with other researches [47, 48]. However, the mechanism how SREBP2 responded to matrix stiffness remained inconsistent.

There have been several compelling evidence that organelle such as ER and Golgi remodeling is associated with lipid metabolism. For example, SREBP2 could be activated by hypoxia-induced Golgi disassembly in bone marrow-derived cells [49]. Additionally, mechanical cues such as stiffness and mechanical strain could regulate the Golgi-related protein secretion [34], influence the formation of the early secretory pathway, and accelerate the ER-to-Golgi transport [35]. The organelle crosstalk that the interactions between ER and Golgi apparatus could facilitate lipids transfer has been proved to play a key role in chronic kidney diseases [50]. Multi-omics analysis demonstrated a significantly positive association between Golgi apparatus dysfunction and lipid metabolism in chronic inflammatory diseases [51]. Therefore, we proposed a scientific hypothesis that Golgi apparatus might link matrix stiffness with SREBP2 activation. As expected, we found Golgi dispersal and Golgi-ER fusion in the HCT116 and HCT15 cells under stiffer substrates. Furthermore, pharmacological induction of Golgi-ER fusion by BFA triggered SREBP2 activation and the upregulation of its downstream targets, recapitulating the phenotype induced by high matrix stiffness. BFA treatment also promoted the degradation of E-cadherin and upregulation of Vimentin and N-cadherin, further supporting the significance of Golgi-ER fusion that coordinates matrix stiffness with cholesterol metabolism and promotes CRC progression.

There are several strengths in our study. First, we innovatively proposed a mechanosensitive organelle remodeling that linked ECM to lipid metabolism. Our findings identified Golgi-ER membrane fusion as a critical event in sensing and transducing mechanical forces, thereby regulating tumor metabolic reprogramming. To our knowledge, this is the first study to establish a direct connection between organelle dynamics and mechano-metabolic coupling in cancer cells. Secondly, out study uncovered a novel metabolism-mediated tumorigenic mechanism that stiffness-activated HMGCR led to the phosphorylation of β-catenin at Ser552, causing it to dissociate from E-cadherin. This disrupted cell-cell adhesion and allowed β-catenin to translocate to the nucleus, driving the EMT event. Thirdly, we developed a dual-targeting strategy that offered clinical translatability of statins and disruption of mechano-signaling, which aligned with emerging therapeutic strategies targeting immunosuppressive microenvironments. However, there are several limitations that should be acknowledged. Firstly, our study primarily utilized cell culture models and animal models, which may not fully recapitulate the complexity of the human tumor microenvironment. Future studies incorporating patient-derived xenografts models or organoid cultures may provide more clinically relevant insights. Secondly, the specific molecular mechanisms how stiff substrates stimulate Golgi dispersal and fusion with ER remain to be elucidated. Thirdly, while we demonstrated the therapeutic potential of combining matrix softening with cholesterol synthesis inhibition, the long-term efficacy and safety of these approaches in clinical settings remain to be determined. Future clinical trials are necessary to validate the effectiveness and feasibility of these combination therapies in CRC patients.

## Conclusion

In conclusion, our study demonstrates that a mechanosensitive Golgi-ER fusion circuit coordinates biophysical cues from ECM with cellular biochemical signaling to promote EMT in CRC. Mechanistically, high matrix stiffness stimulates Golgi dispersal and Golgi-ER fusion, thereby activating SREBP2 and upregulating its downstream HMGCR expression. This event enhances cholesterol biosynthesis and subsequent oncogenic signaling activation. Pharmacologic ECM softening and suppression of cholesterol synthesis restrain tumor growth in both orthotopic and carcinogen-induced CRC models without overt toxicity. By elucidating a mechano-metabolic circuit, our study provides a compelling mechanistic rationale for integrating ECM-modulating agents with mevalonate pathway inhibitors. This combination strategy offers a novel therapeutic blueprint to improve treatment efficacy in CRC and potentially other solid malignancies.

## Supplementary information

SUPPLEMENTAL MATERIAL--SP-Table and SP-Figure legend

SUPPLEMENTAL MATERIAL--SP-Figure

SUPPLEMENTAL MATERIAL--Unropped WB Figures

## Data Availability

This paper doesn’t report original code. Any additional information required to re-analyze the data reported in this paper is available from the lead contact upon request.
