# Supplementary material for: Matrix stiffness promotes colorectal cancer progression via Golgi-dependent cholesterol synthesis
Source: Cell Death Dis. 2026 Jul 24;17(1):894. doi: 10.1038/s41419-026-09101-5 (PMC13680387; doi:10.1038/s41419-026-09101-5)
Supplement: Supplementary file 1 [file 41419_2026_9101_MOESM2_ESM.pdf]

Figure S1

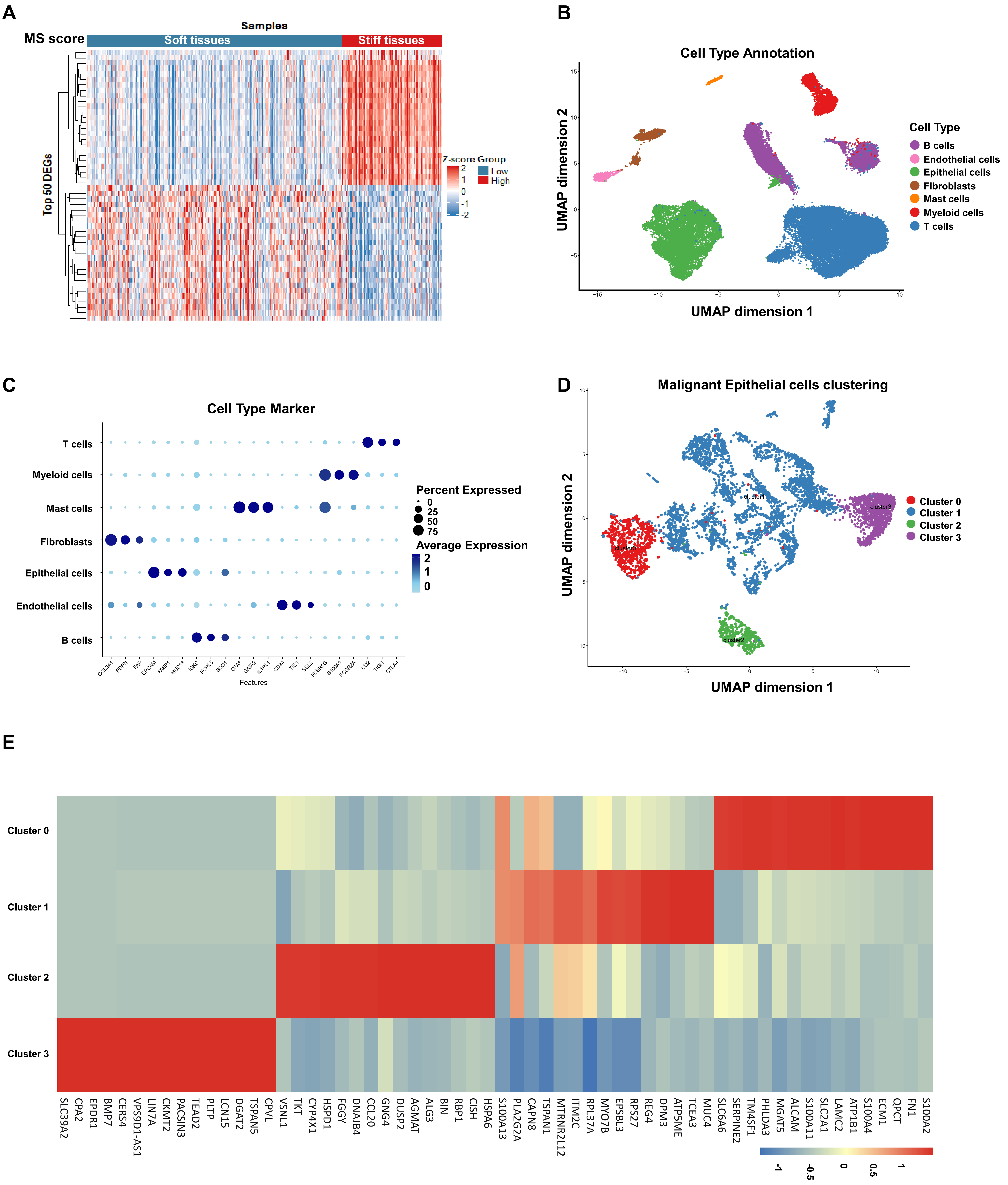

A
Samples
MS score
Soft tissues
Stiff tissues
Top 50 DEGs
Z-score
2
1
0
-1
-2
Group
Low
High
B
Cell Type Annotation
UMAP dimension 2
UMAP dimension 1
Cell Type
B cells
Endothelial cells
Epithelial cells
Fibroblasts
Mast cells
Myeloid cells
T cells
C
Cell Type Marker
T cells
Myeloid cells
Mast cells
Fibroblasts
Epithelial cells
Endothelial cells
B cells
Features
Percent Expressed
0
25
50
75
Average Expression
2
1
0
D
Malignant Epithelial cells clustering
UMAP dimension 2
UMAP dimension 1
Cluster 0
Cluster 1
Cluster 2
Cluster 3
E
Cluster 0
Cluster 1
Cluster 2
Cluster 3
SLC39A2
CPA2
EPDR1
BMP7
CERS4
VPS9D1-AS1
LIN7A
CKMT2
PACSIN3
TEAD2
PLTP
LCN15
DGAT2
TSPAN5
CPVL
VSNL1
TKT
CYP4X1
HSPD1
FGGY
DNAJB4
CCL20
GNG4
DUSP2
AGMAT
ALG3
BIN
RBP1
CISH
HSPA6
S100A13
PLA2G2A
CAPN8
TSPAN1
MTRNR2L12
ITM2C
RPL37A
MYO7B
EPS8L3
RPS27
REG4
DPM3
ATP5ME
TCEA3
MUC4
SLC6A6
SERPINE2
TM4SF1
PHLDA3
MGAT5
ALCAM
S100A11
SLC2A1
LAMC2
ATP1B1
S100A4
ECM1
QPCT
FN1
S100A2
-1
-0.5
0
0.5
1

Figure S2

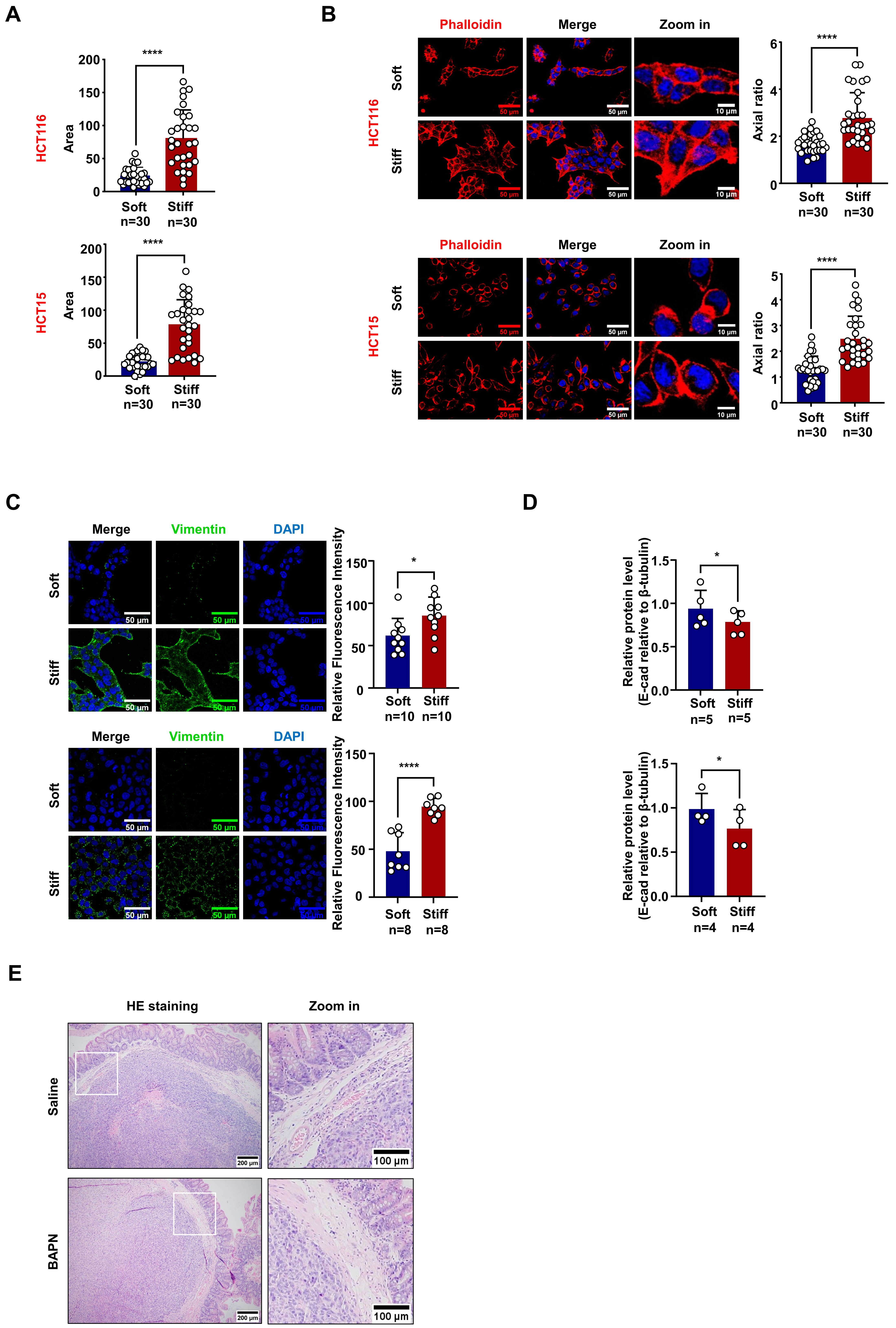
A
HCT116
Area
200
150
100
50
0
****
Soft n=30
Stiff n=30
HCT15
B
Phalloidin
Merge
Zoom in
Soft
Stiff
Axial ratio
50 μm
10 μm
C
Vimentin
DAPI
Relative Fluorescence Intensity
Soft n=10
Stiff n=10
Soft n=8
Stiff n=8
D
Relative protein level (E-cad relative to β-tubulin)
Soft n=5
Stiff n=5
Soft n=4
Stiff n=4
E
HE staining
Saline
BAPN
200 μm
100 μm

Figure S3

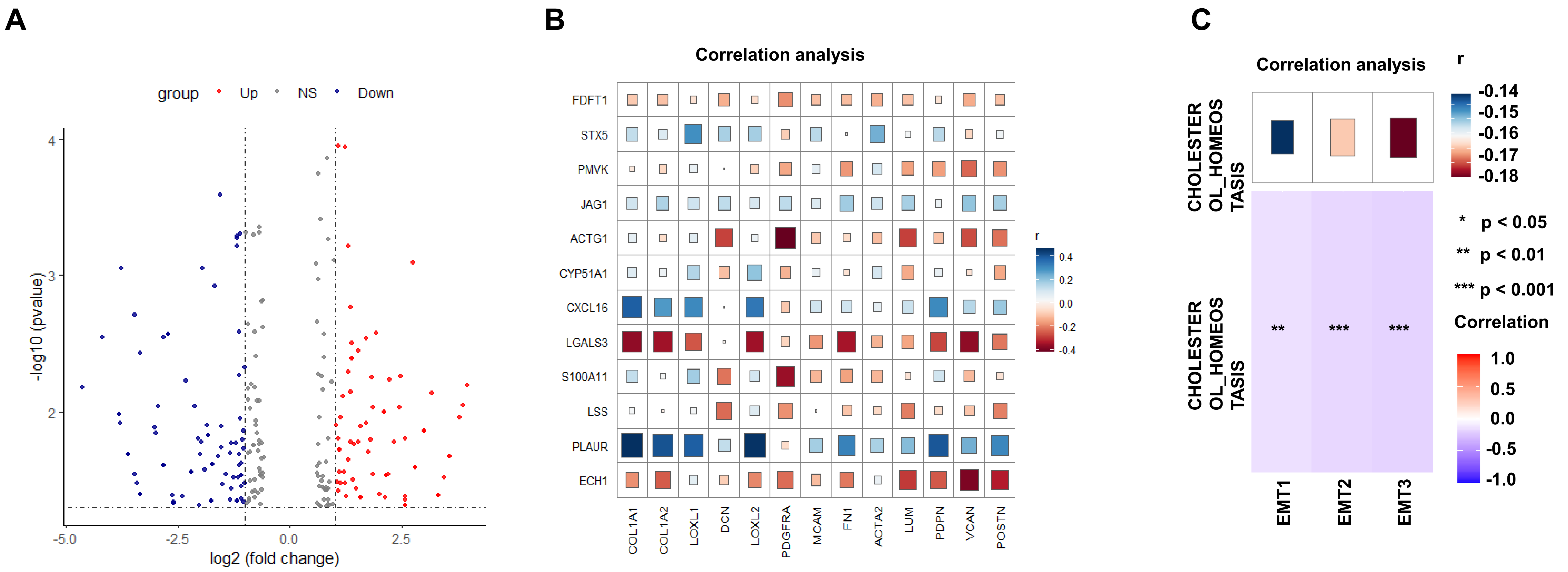
A
group Up NS Down
-log10 (pvalue)
log2 (fold change)
B
Correlation analysis
FDFT1
STX5
PMVK
JAG1
ACTG1
CYP51A1
CXCL16
LGALS3
S100A11
LSS
PLAUR
ECH1
COL1A1
COL1A2
LOXL1
DCN
LOXL2
PDGFRA
MCAM
FN1
ACTA2
LUM
PDPN
VCAN
POSTN
r
C
Correlation analysis
CHOLESTEROL_HOMEOSTASIS
EMT1
EMT2
EMT3
r
* p < 0.05
** p < 0.01
*** p < 0.001
Correlation

Figure S4

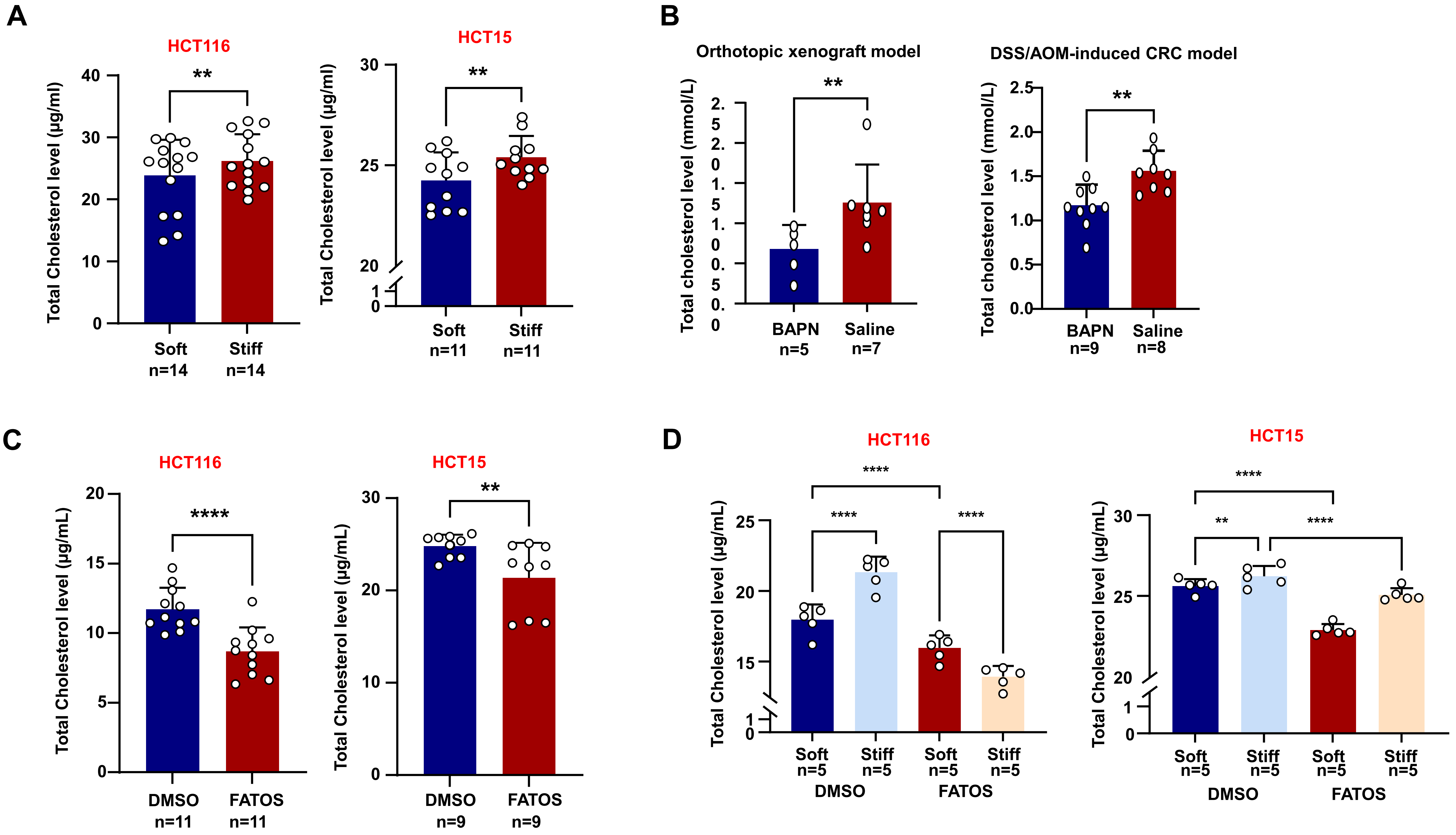

A
HCT116
Total Cholesterol level (μg/ml)
40
30
20
10
0
**
Soft
n=14
Stiff
n=14
HCT15
Total Cholesterol level (μg/ml)
30
25
20
1
0
**
Soft
n=11
Stiff
n=11
B
Orthotopic xenograft model
Total cholesterol level (mmol/L)
**
BAPN
n=5
Saline
n=7
DSS/AOM-induced CRC model
Total cholesterol level (mmol/L)
2.5
2.0
1.5
1.0
0.5
0.0
**
BAPN
n=9
Saline
n=8
C
HCT116
Total Cholesterol level (μg/mL)
20
15
10
5
0
****
DMSO
n=11
FATOS
n=11
HCT15
Total Cholesterol level (μg/mL)
30
20
10
0
**
DMSO
n=9
FATOS
n=9
D
HCT116
Total Cholesterol level (μg/mL)
25
20
15
1
0
****
****
****
Soft
n=5
Stiff
n=5
Soft
n=5
Stiff
n=5
DMSO
FATOS
HCT15
Total Cholesterol level (μg/mL)
30
25
20
1
0
****
**
****
Soft
n=5
Stiff
n=5
Soft
n=5
Stiff
n=5
DMSO
FATOS

Figure S5

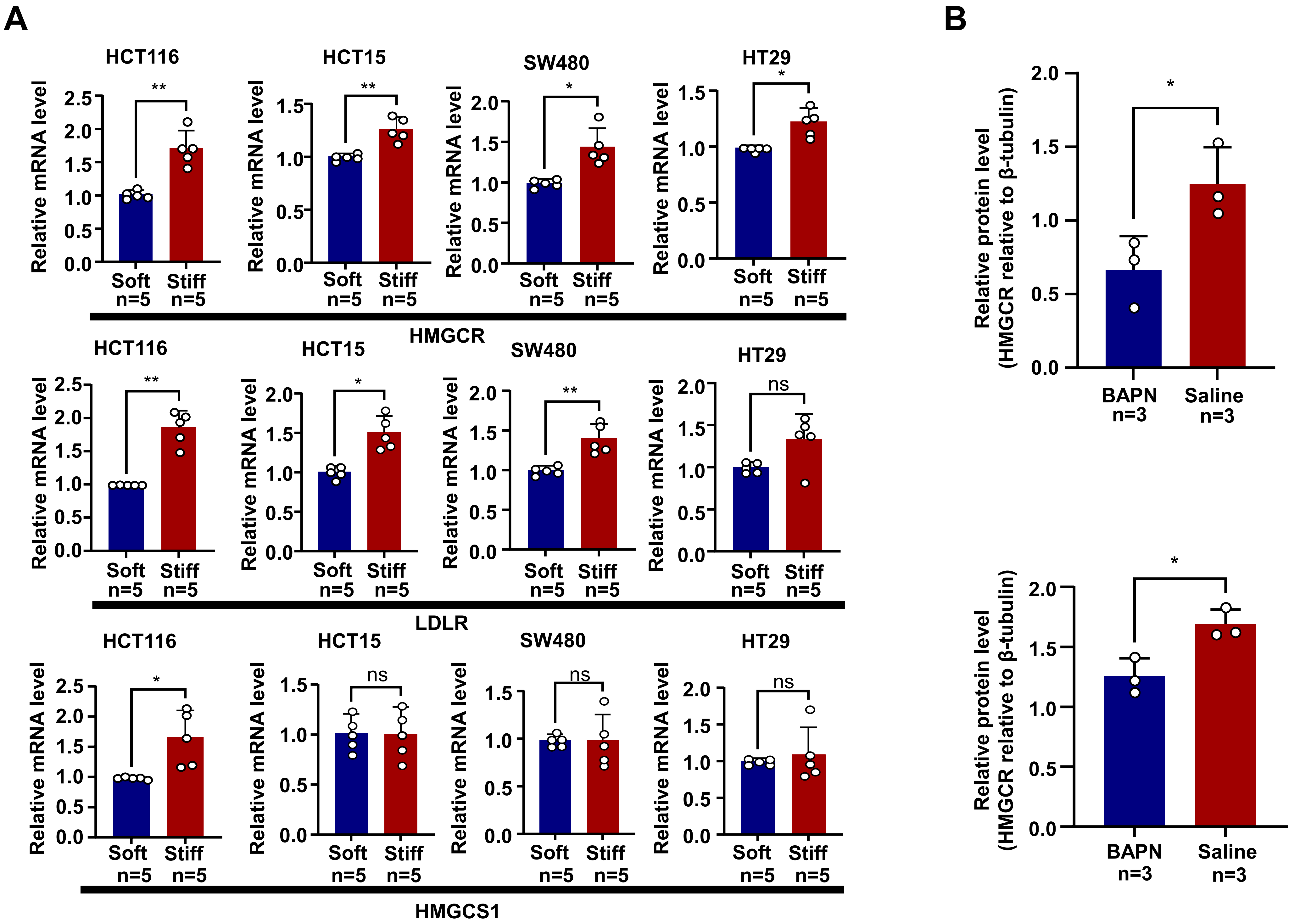

A
HCT116
HCT15
SW480
HT29
Relative mRNA level
Soft n=5
Stiff n=5
HMGCR
LDLR
HMGCS1
**
*
ns
B
Relative protein level (HMGCR relative to β-tubulin)
BAPN n=3
Saline n=3

Figure S6

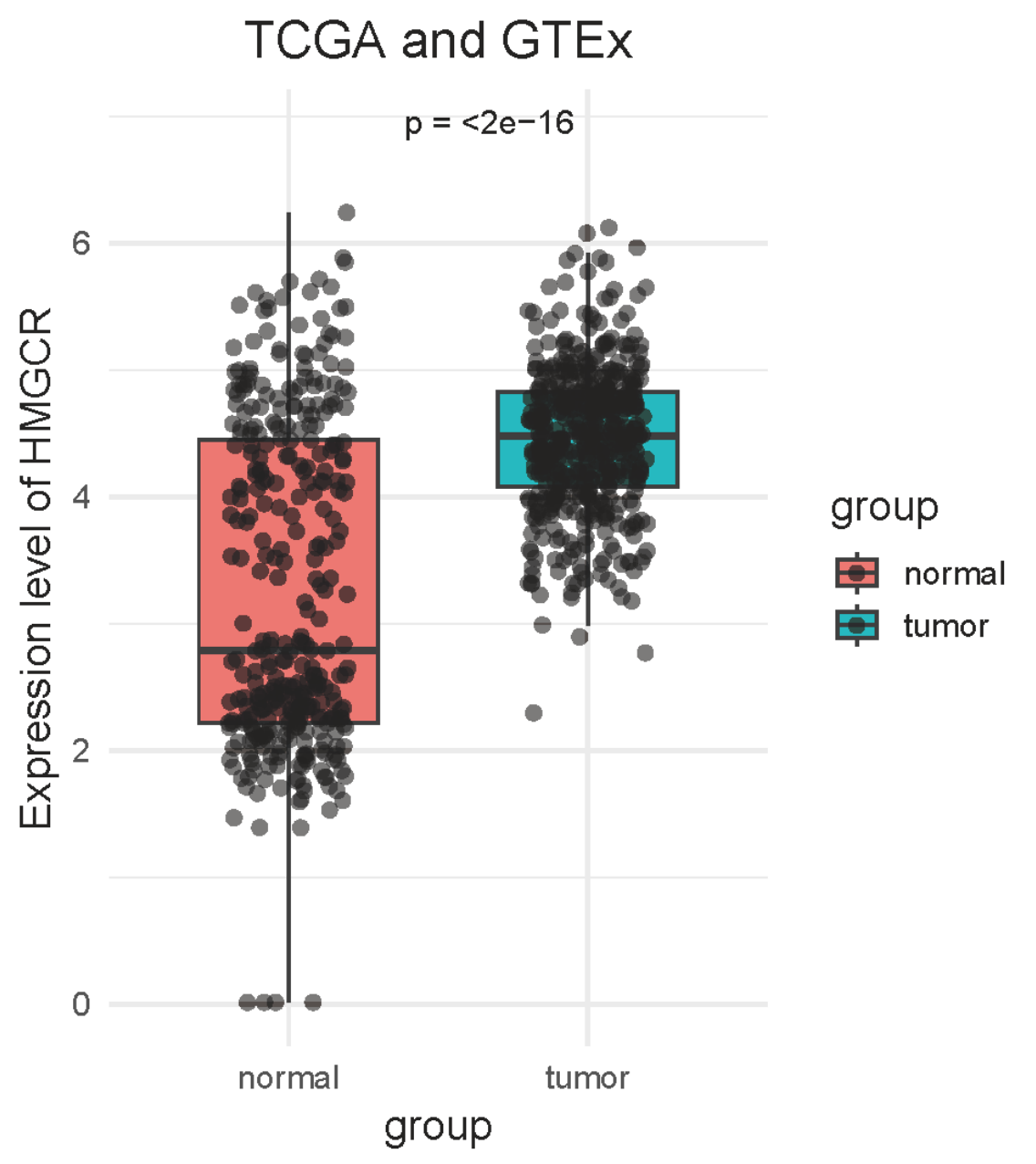
TCGA and GTEx
p = <2e−16
Expression level of HMGCR
6
4
2
0
normal
tumor
group
group
normal
tumor

Figure S7

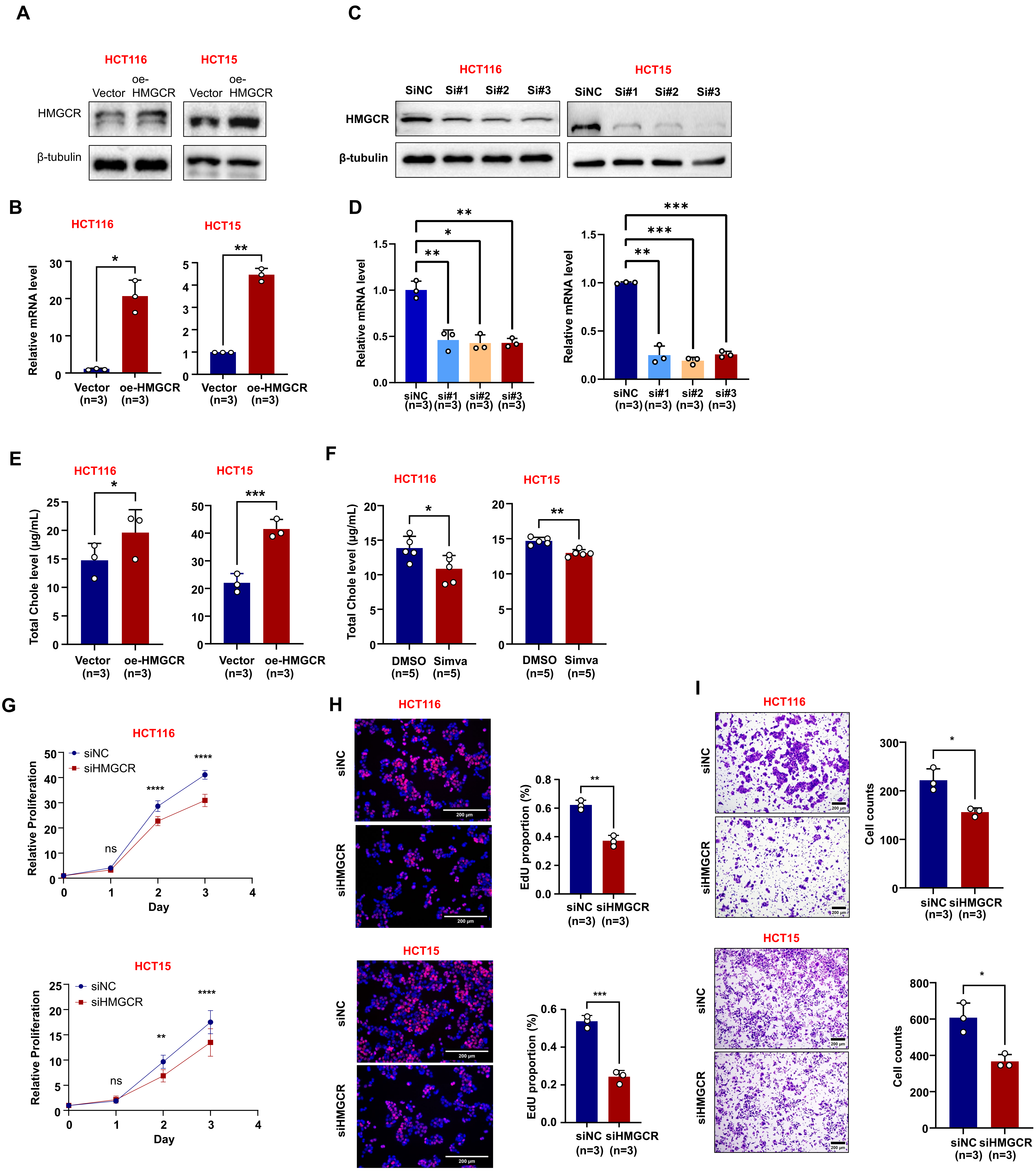
A
HCT116
HCT15
Vector
oe-HMGCR
HMGCR
β-tubulin
C
SiNC
Si#1
Si#2
Si#3
B
Relative mRNA level
Vector (n=3)
oe-HMGCR (n=3)
D
siNC (n=3)
si#1 (n=3)
si#2 (n=3)
si#3 (n=3)
E
Total Chole level (μg/mL)
F
DMSO (n=5)
Simva (n=5)
G
siNC
siHMGCR
Relative Proliferation
Day
ns
H
EdU proportion (%)
200 μm
I
Cell counts

Figure S8

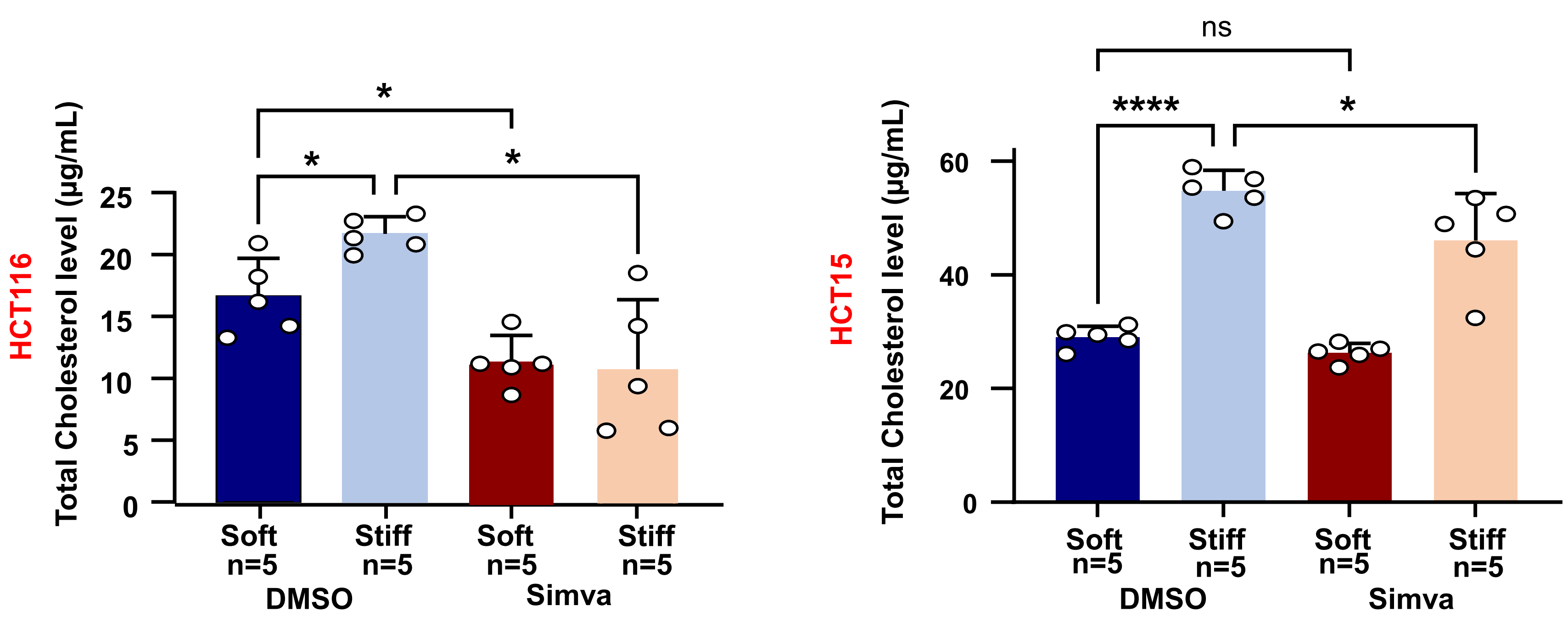

HCT116
Total Cholesterol level (µg/mL)
25
20
15
10
5
0
*
*
*
Soft n=5
Stiff n=5
Soft n=5
Stiff n=5
DMSO
Simva
HCT15
Total Cholesterol level (µg/mL)
60
40
20
0
ns
****
*
Soft n=5
Stiff n=5
Soft n=5
Stiff n=5
DMSO
Simva

Figure S9

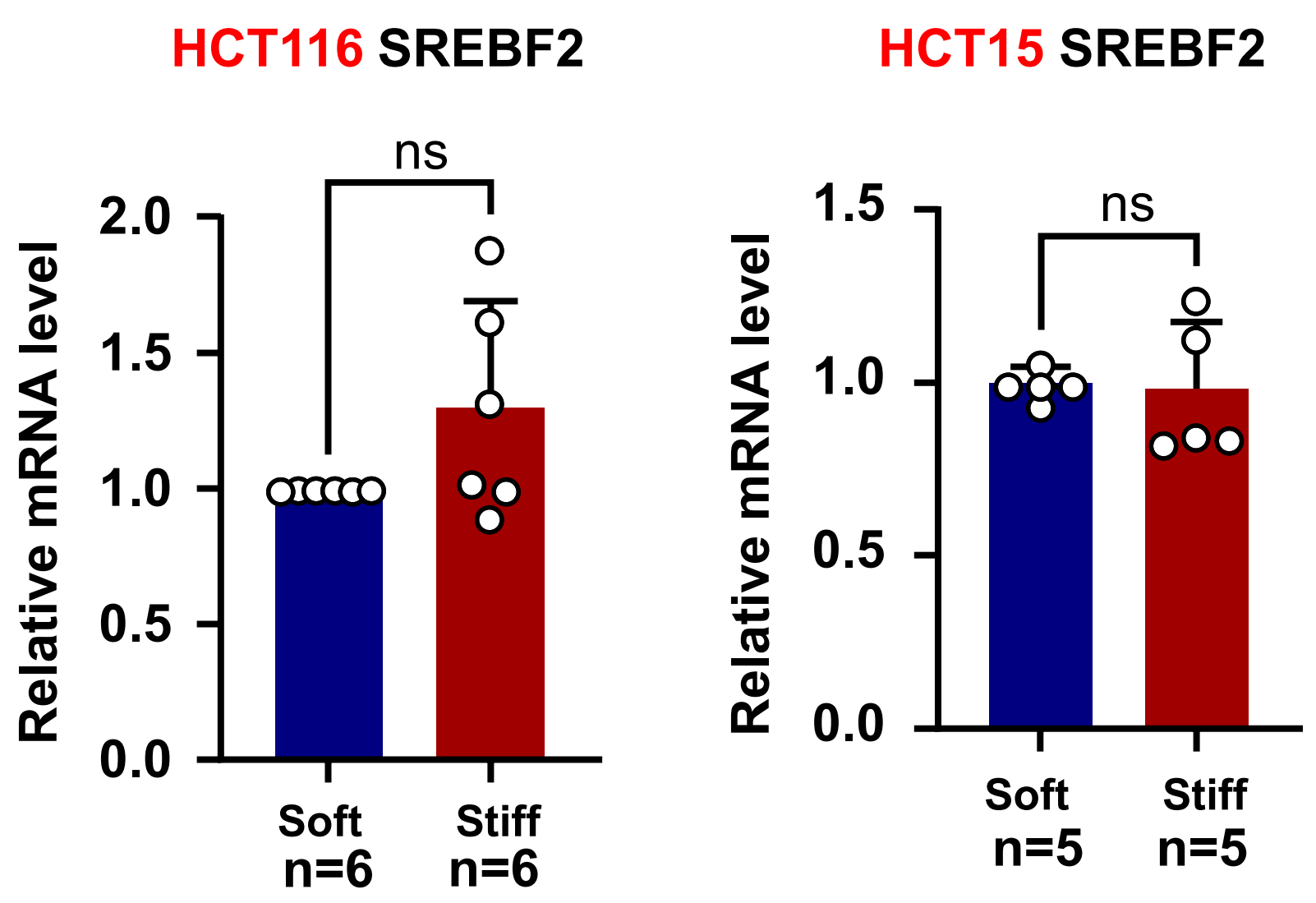
HCT116 SREBF2
ns
Relative mRNA level
2.0
1.5
1.0
0.5
0.0
Soft
n=6
Stiff
n=6
HCT15 SREBF2
ns
Relative mRNA level
1.5
1.0
0.5
0.0
Soft
n=5
Stiff
n=5

Figure S10

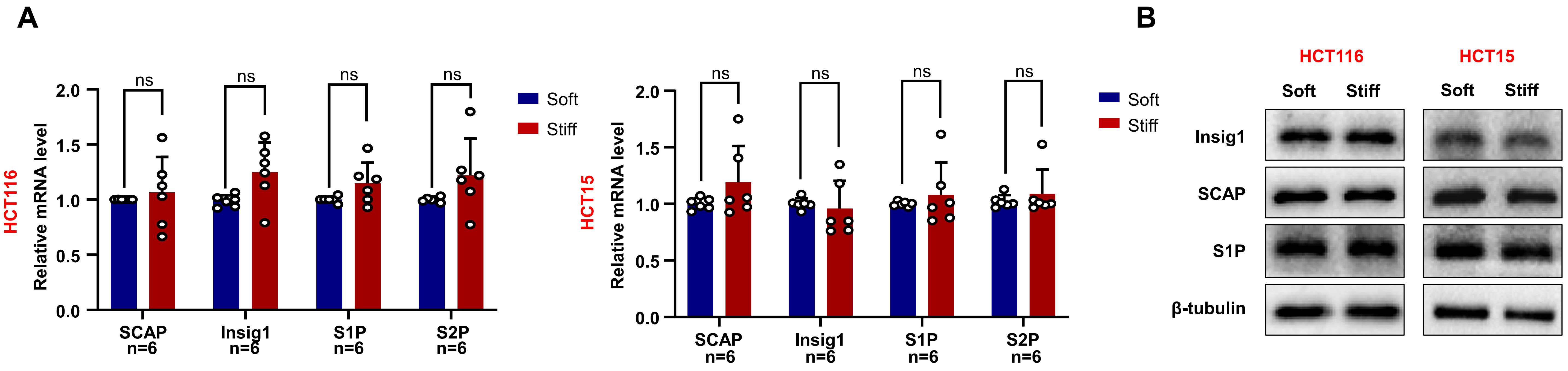

A
B
HCT116
Relative mRNA level
2.0
1.5
1.0
0.5
0.0
ns
SCAP
n=6
Insig1
n=6
S1P
n=6
S2P
n=6
Soft
Stiff
HCT15
HCT116
HCT15
Soft
Stiff
Insig1
SCAP
S1P
β-tubulin

Figure S11

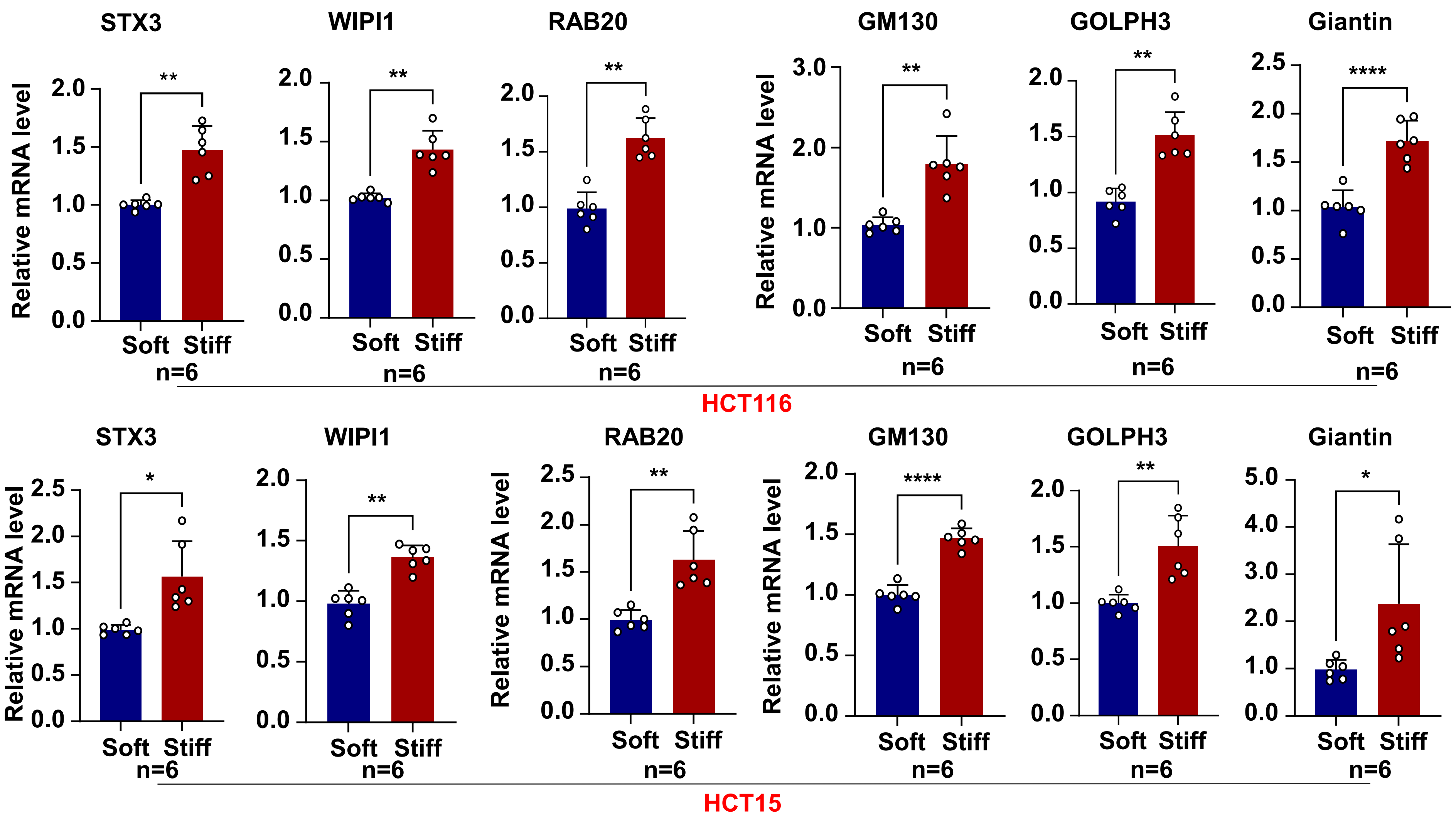

STX3
WIPI1
RAB20
GM130
GOLPH3
Giantin
Relative mRNA level
Soft
Stiff
n=6
HCT116
HCT15

Figure S12

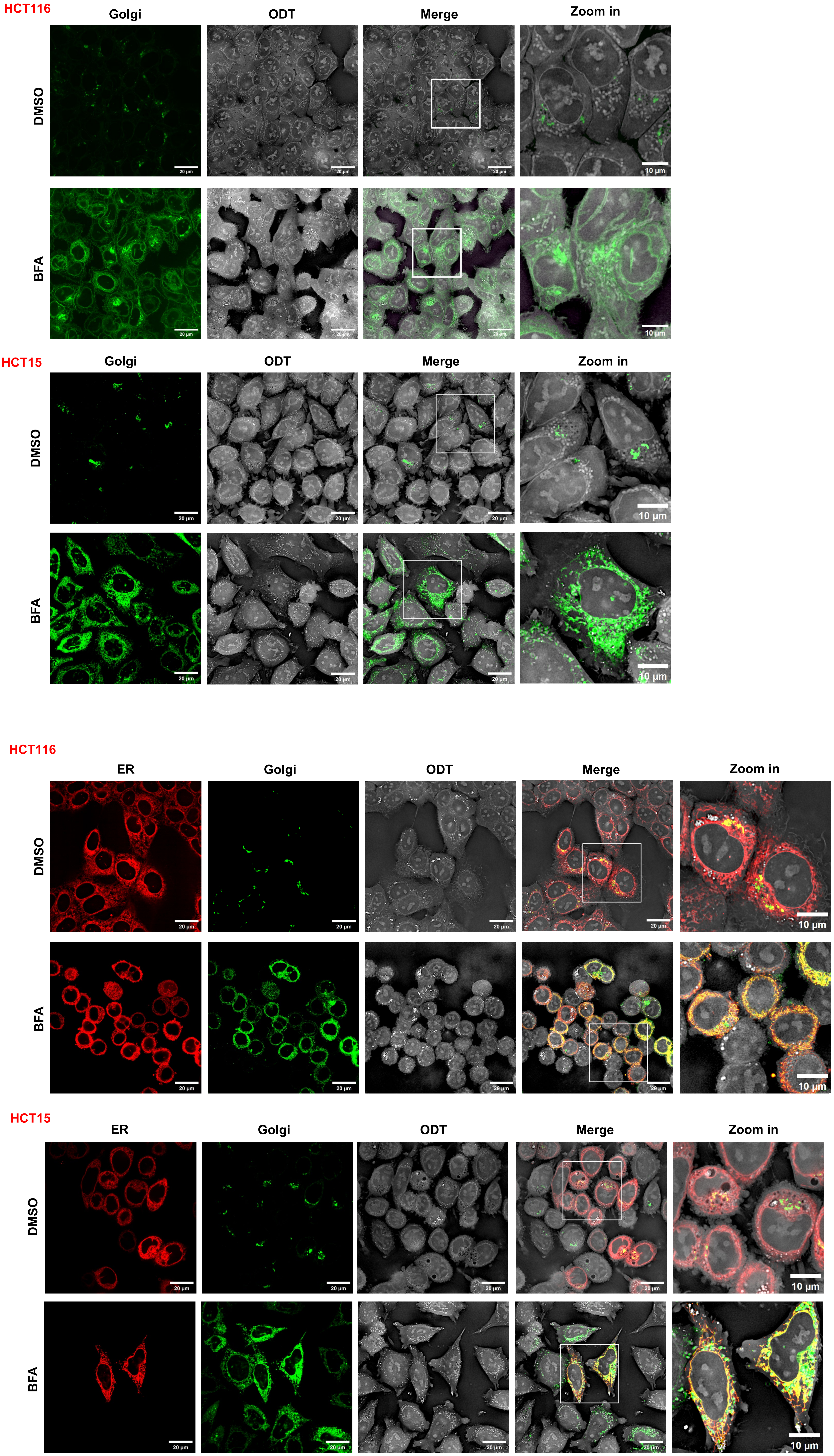

HCT116
Golgi
ODT
Merge
Zoom in
DMSO
BFA
10 µm
HCT15
Golgi
ODT
Merge
Zoom in
DMSO
BFA
20 µm
10 µm
HCT116
ER
Golgi
ODT
Merge
Zoom in
DMSO
BFA
20 µm
10 µm
HCT15
ER
Golgi
ODT
Merge
Zoom in
DMSO
BFA
20 µm
10 µm

Figure S13

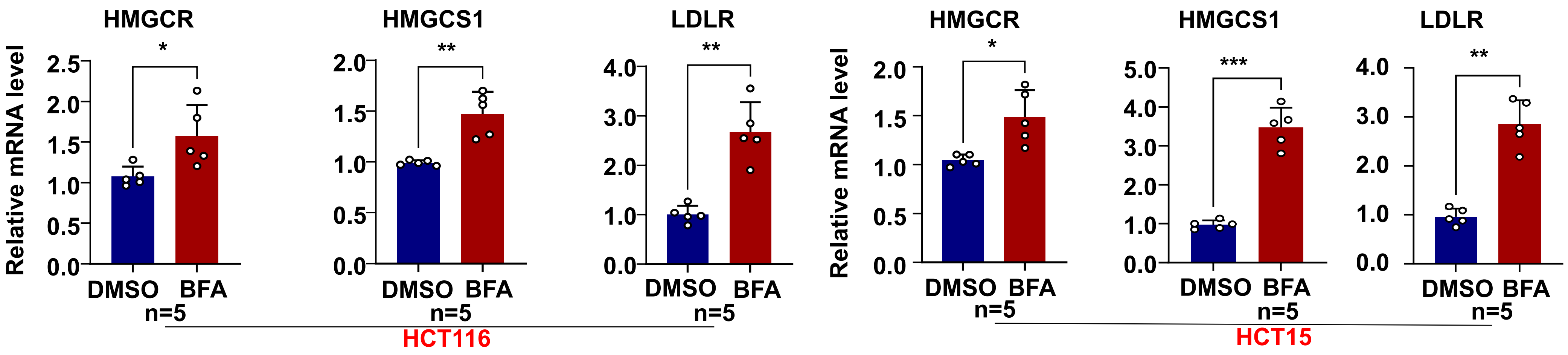

HMGCR
HMGCS1
LDLR
HMGCR
HMGCS1
LDLR
Relative mRNA level
DMSO BFA
n=5
HCT116
HCT15

Figure S14

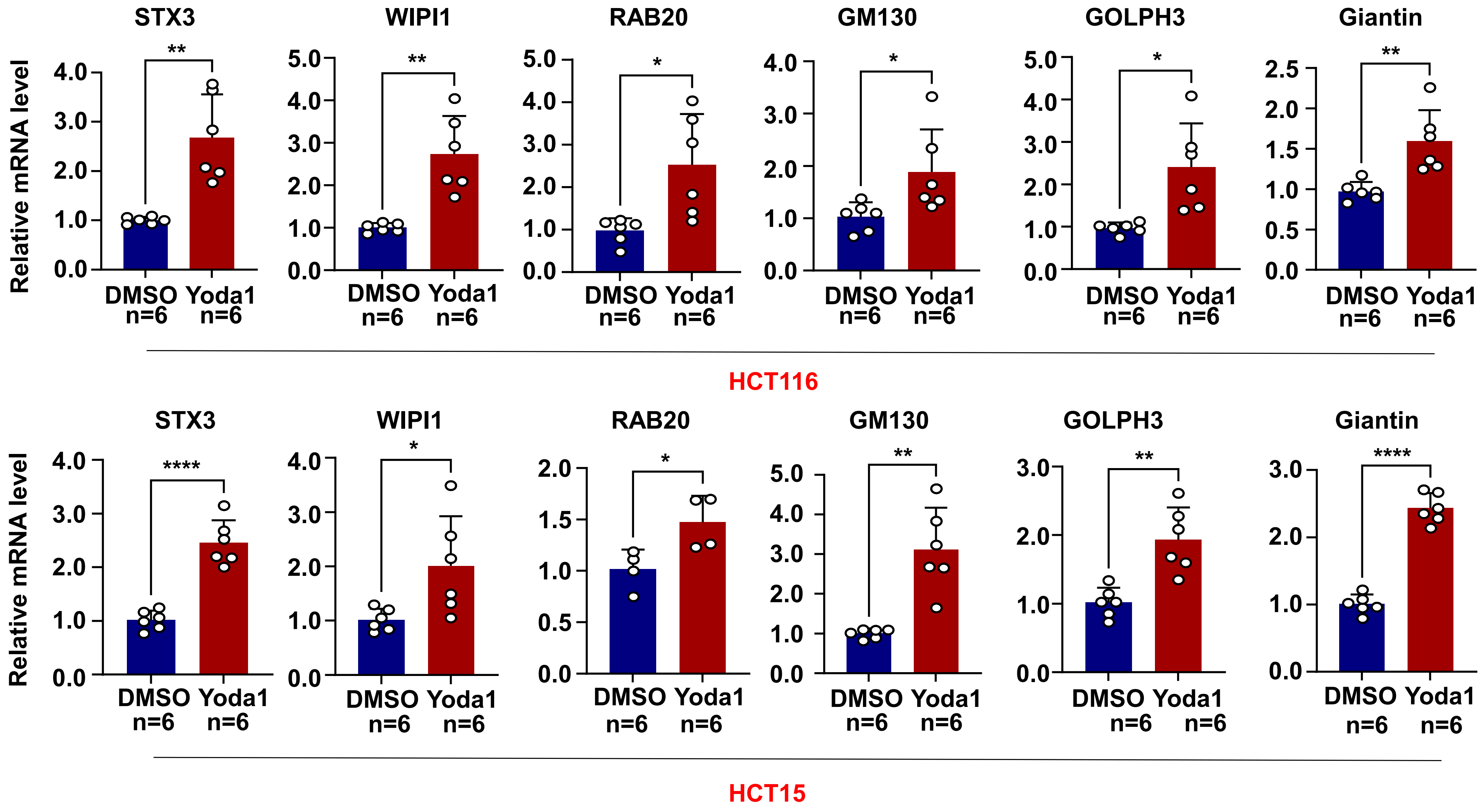
STX3
WIPI1
RAB20
GM130
GOLPH3
Giantin
Relative mRNA level
DMSO n=6
Yoda1 n=6
HCT116
HCT15
