# Supplementary material for: Matrix stiffness promotes colorectal cancer progression via Golgi-dependent cholesterol synthesis
Source: Cell Death Dis. 2026 Jul 24;17(1):894. doi: 10.1038/s41419-026-09101-5 (PMC13680387; doi:10.1038/s41419-026-09101-5)
Supplement: Supplementary file 2 [file 41419_2026_9101_MOESM3_ESM.pdf]

Figure 1

H

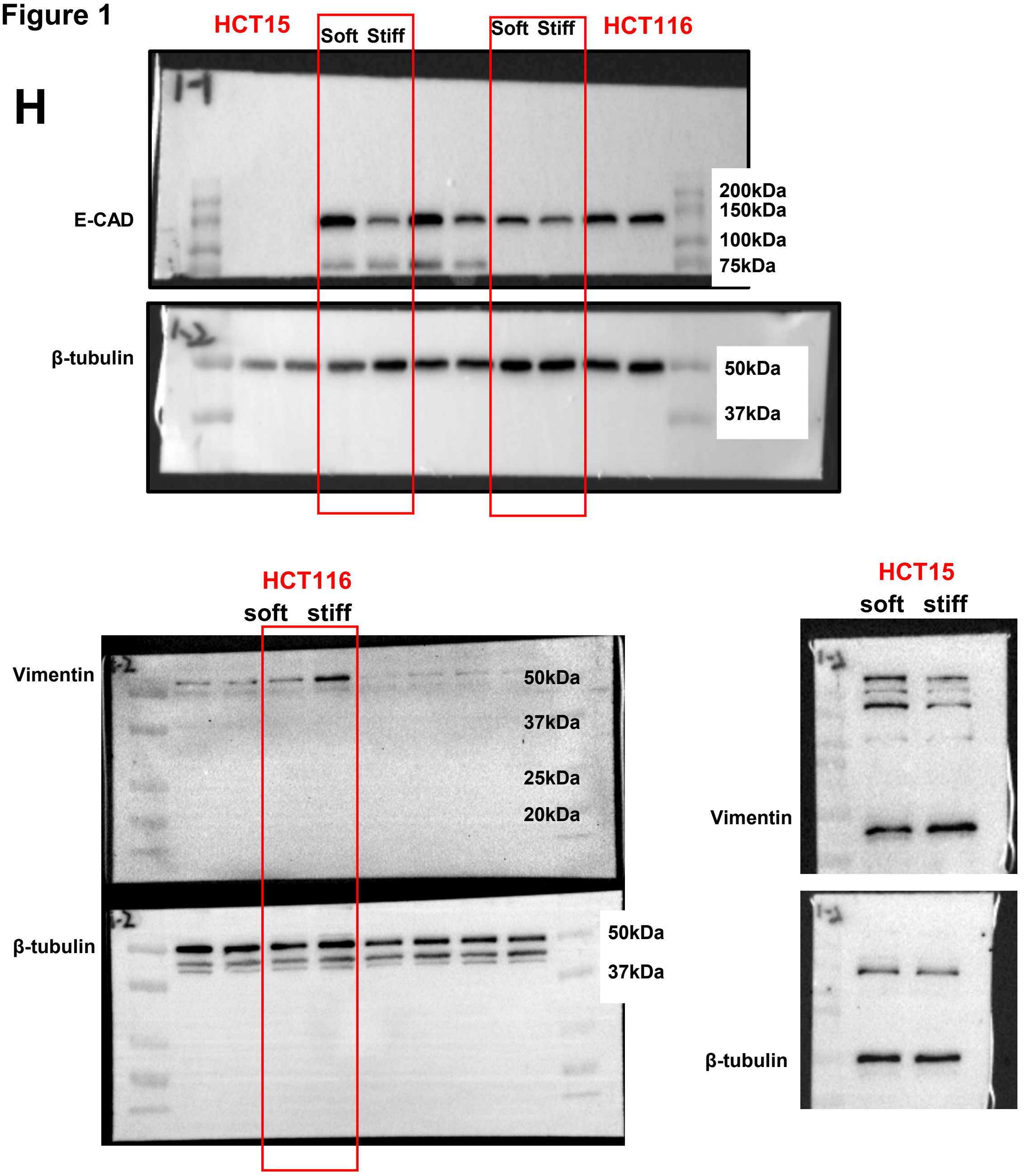
HCT15
Soft Stiff
Soft Stiff
HCT116
E-CAD
200kDa
150kDa
100kDa
75kDa
β-tubulin
50kDa
37kDa
HCT116
soft stiff
Vimentin
50kDa
37kDa
25kDa
20kDa
β-tubulin
50kDa
37kDa
HCT15
soft stiff
Vimentin
β-tubulin

Figure 1

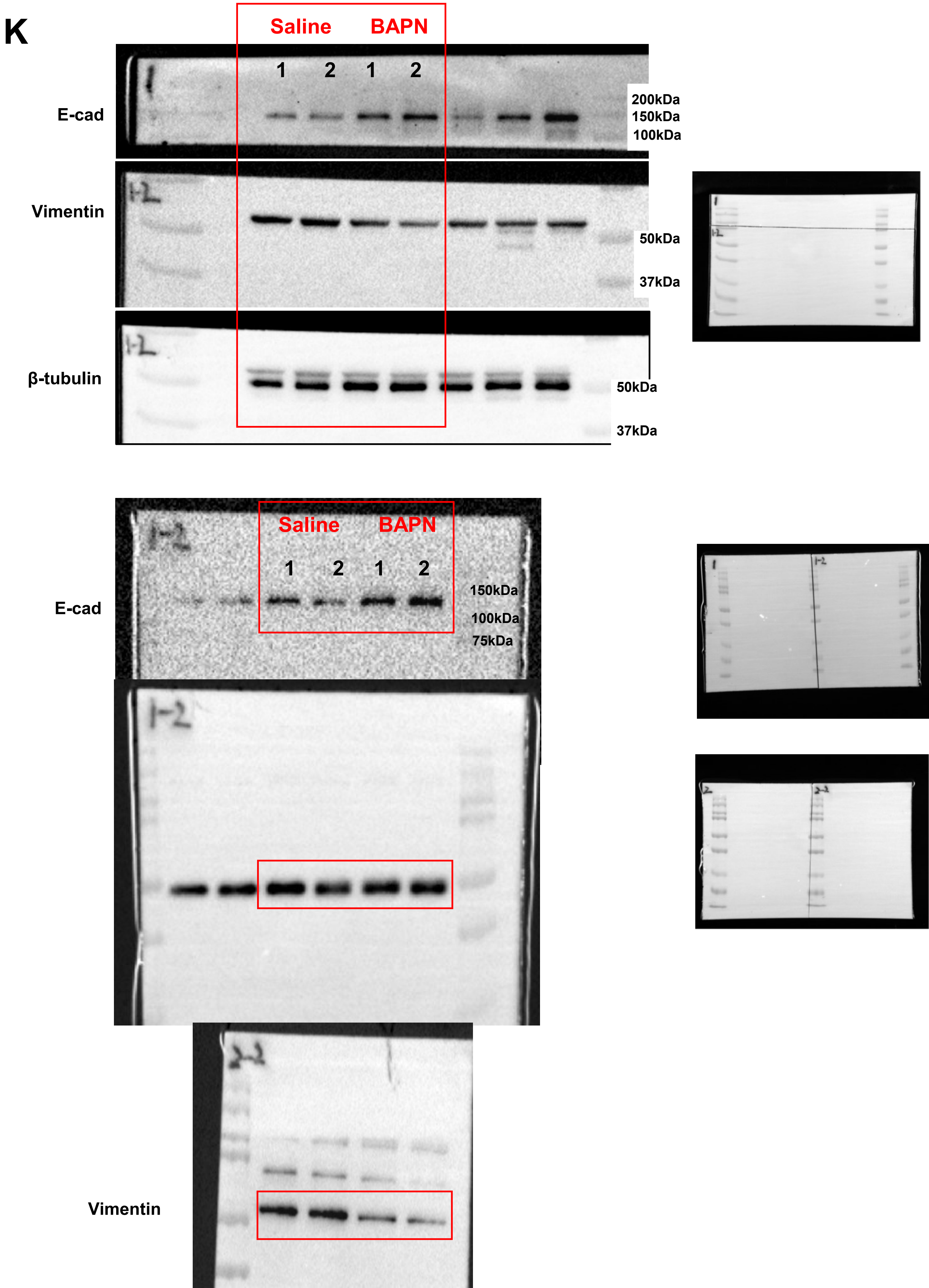
K
Saline
BAPN
1
2
1
2
E-cad
200kDa
150kDa
100kDa
Vimentin
50kDa
37kDa
β-tubulin
50kDa
37kDa
Saline
BAPN
1
2
1
2
E-cad
150kDa
100kDa
75kDa
Vimentin

Figure 2

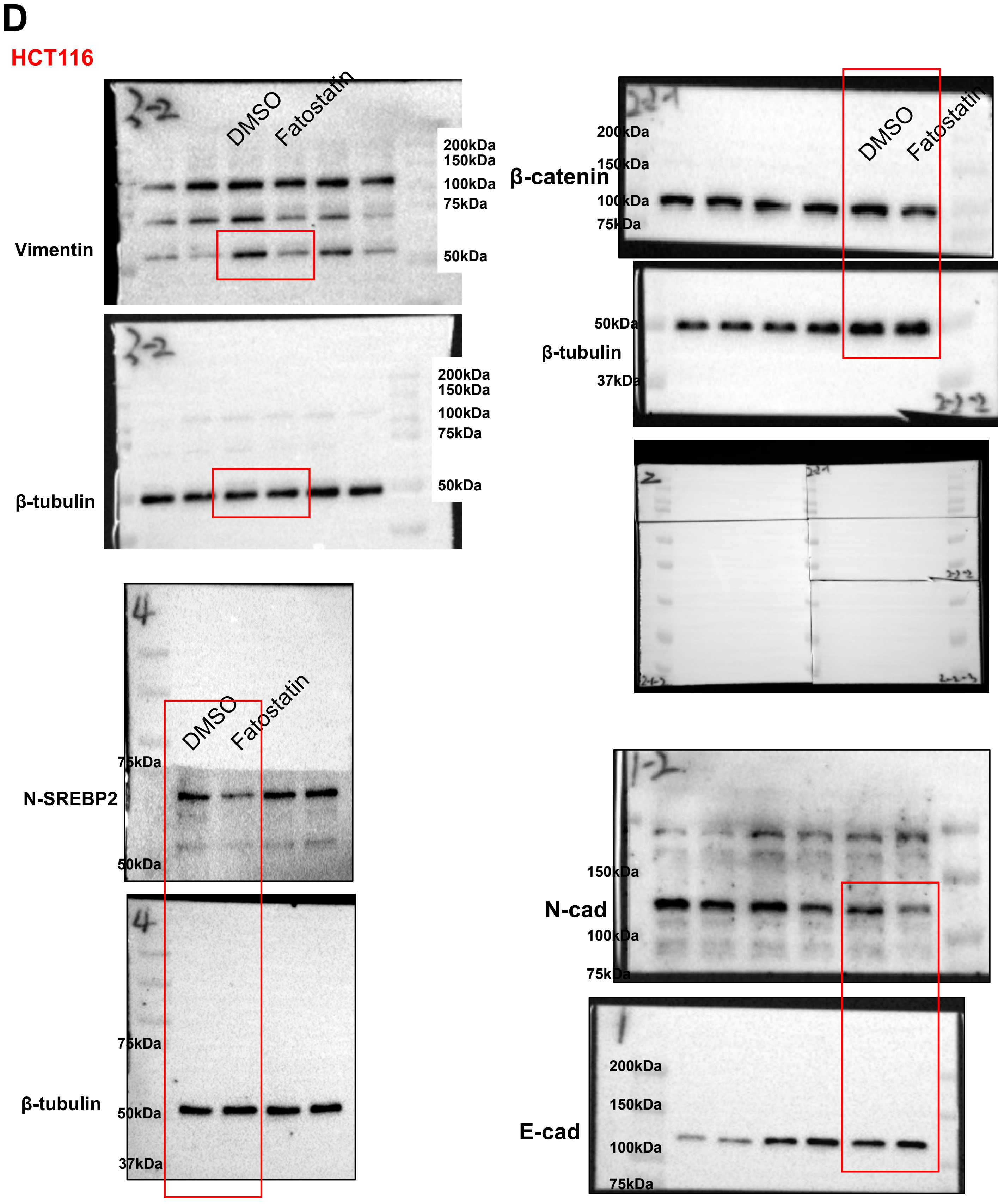
D
HCT116
DMSO
Fatostatin
200kDa
150kDa
100kDa
75kDa
50kDa
Vimentin
β-tubulin
β-catenin
37kDa
N-SREBP2
N-cad
E-cad

Figure 2

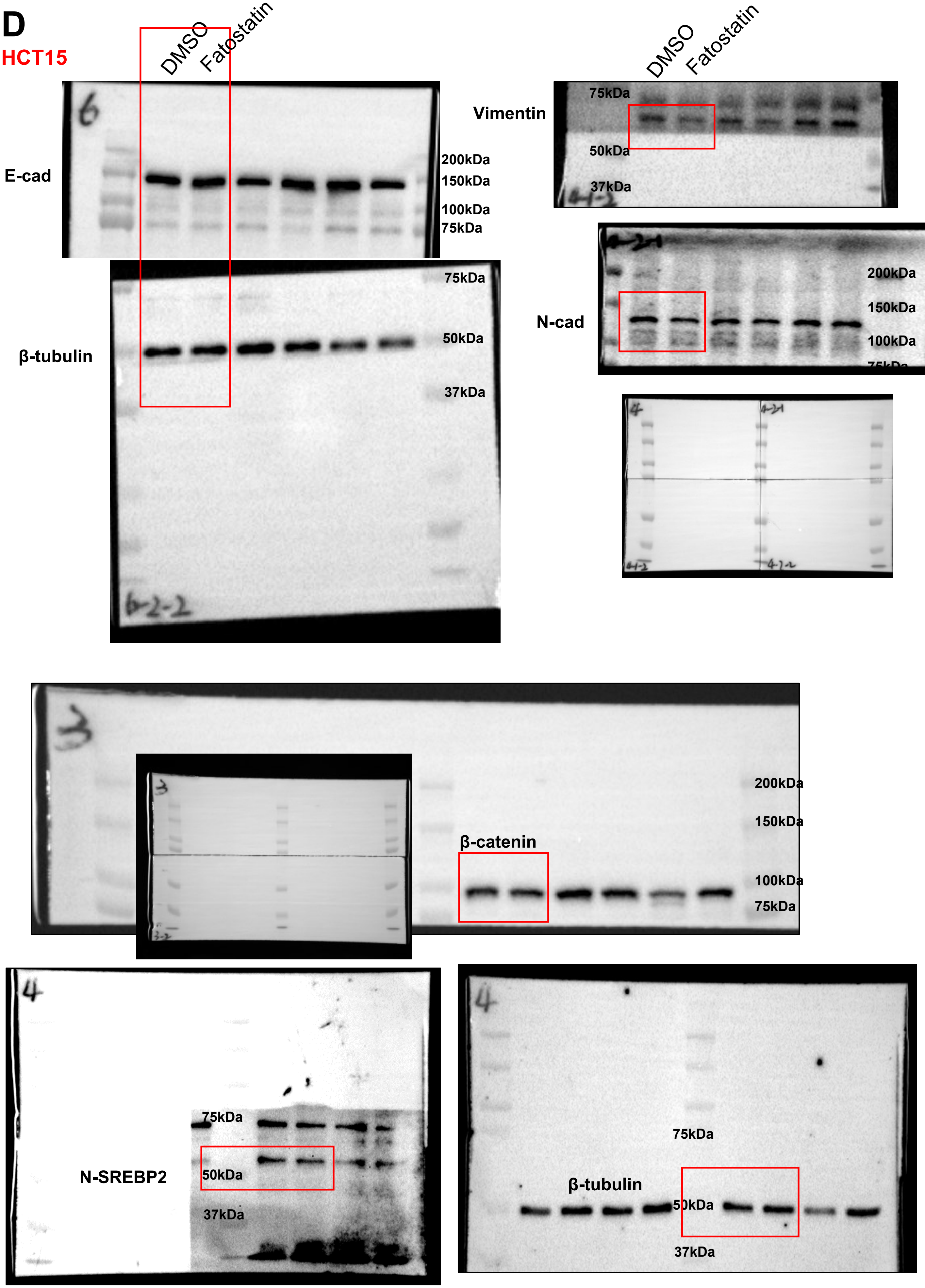
D
HCT15
DMSO
Fatostatin
E-cad
200kDa
150kDa
100kDa
75kDa
β-tubulin
75kDa
50kDa
37kDa
DMSO
Fatostatin
Vimentin
75kDa
50kDa
37kDa
N-cad
200kDa
150kDa
100kDa
β-catenin
200kDa
150kDa
100kDa
75kDa
N-SREBP2
75kDa
50kDa
37kDa
β-tubulin
75kDa
50kDa
37kDa

Figure 2

F

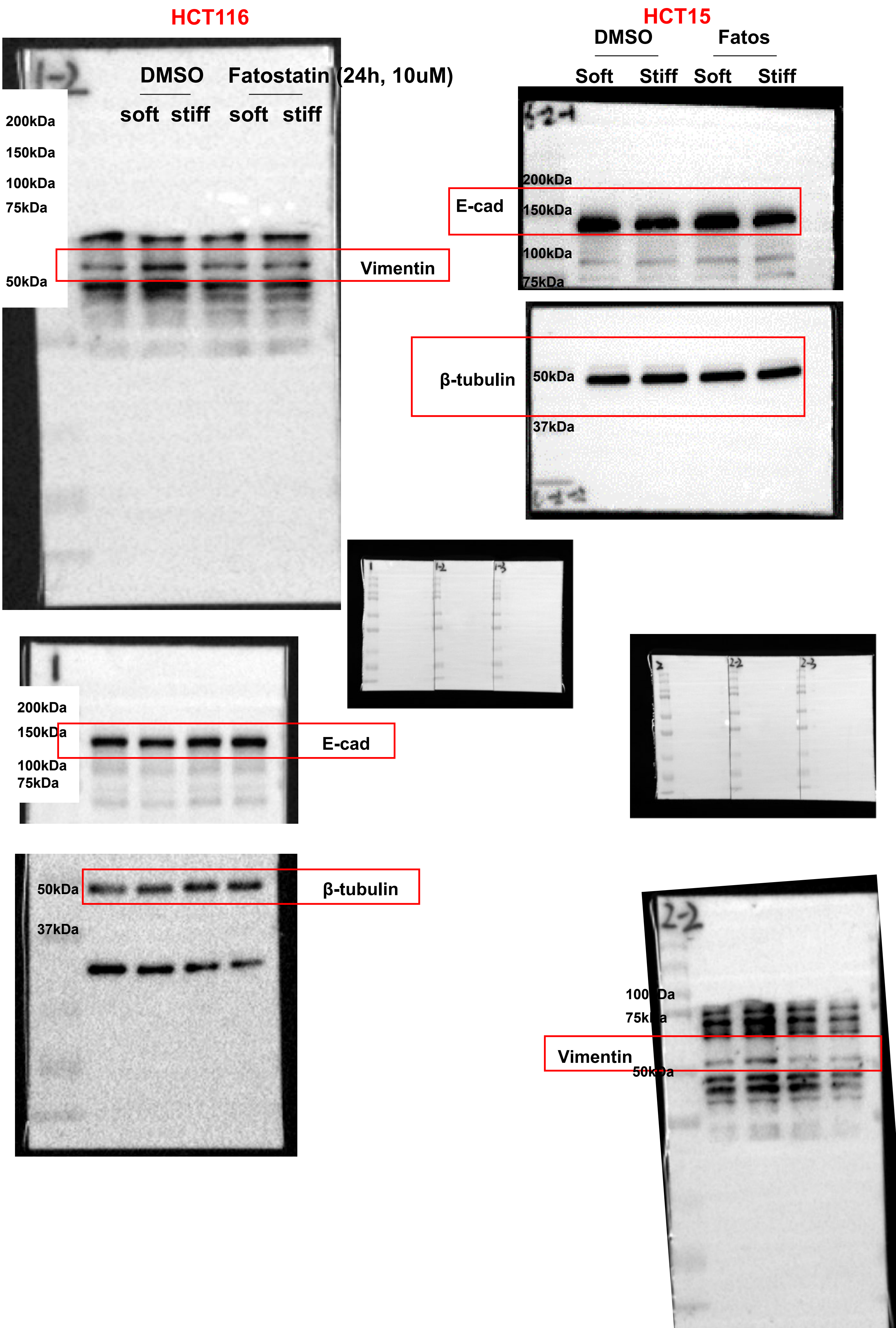
HCT116
DMSO
Fatostatin (24h, 10uM)
soft stiff soft stiff
200kDa
150kDa
100kDa
75kDa
50kDa
Vimentin
200kDa
150kDa
100kDa
75kDa
E-cad
50kDa
37kDa
β-tubulin
HCT15
DMSO
Fatos
Soft Stiff Soft Stiff
200kDa
150kDa
100kDa
75kDa
E-cad
50kDa
37kDa
β-tubulin
Vimentin

Figure 2

H

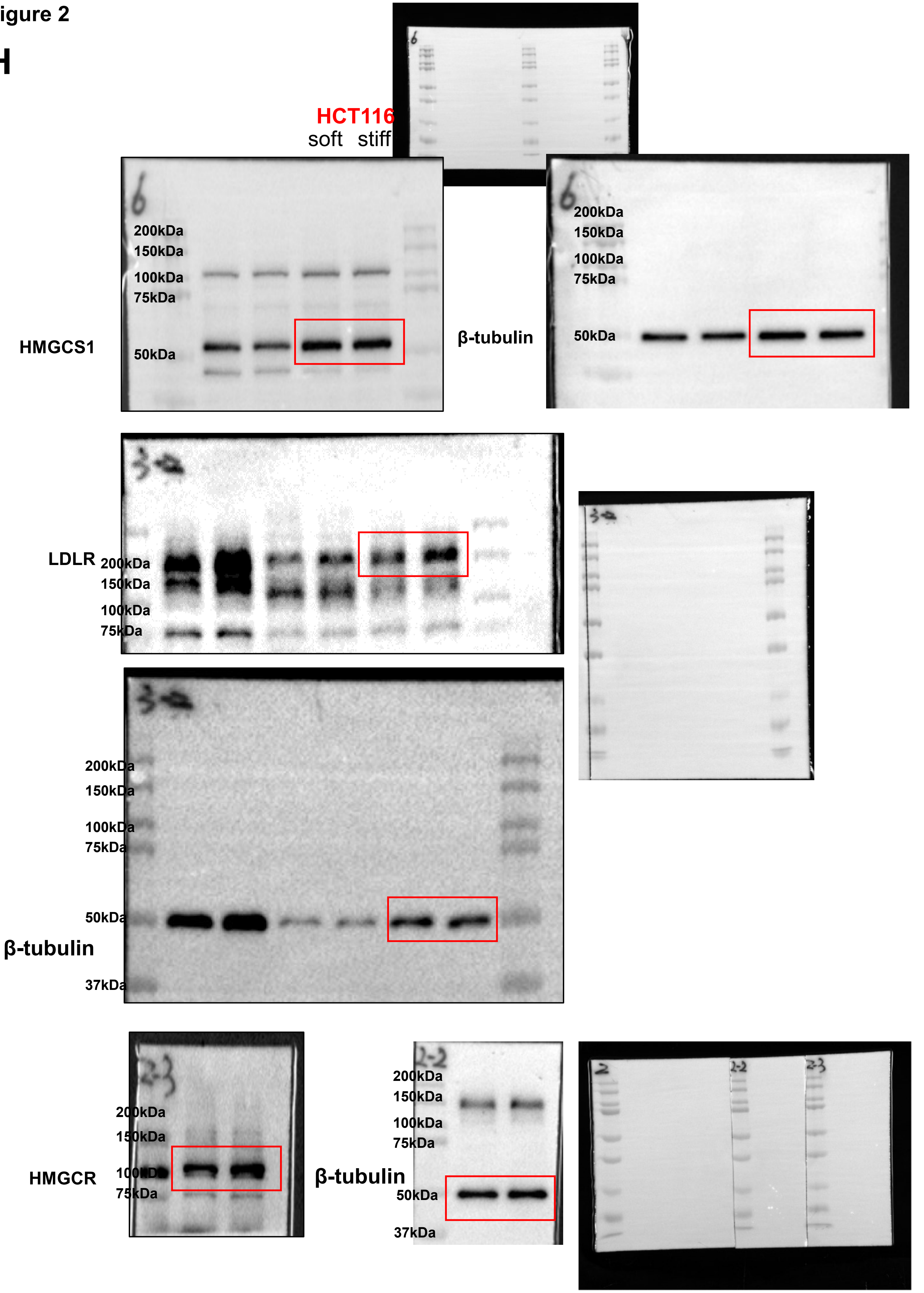
HCT116
soft
stiff
HMGCS1
200kDa
150kDa
100kDa
75kDa
50kDa
β-tubulin
200kDa
150kDa
100kDa
75kDa
50kDa
LDLR
200kDa
150kDa
100kDa
75kDa
β-tubulin
200kDa
150kDa
100kDa
75kDa
50kDa
37kDa
HMGCR
200kDa
150kDa
100kDa
75kDa
β-tubulin
200kDa
150kDa
100kDa
75kDa
50kDa
37kDa

Figure 2 HCT15

H

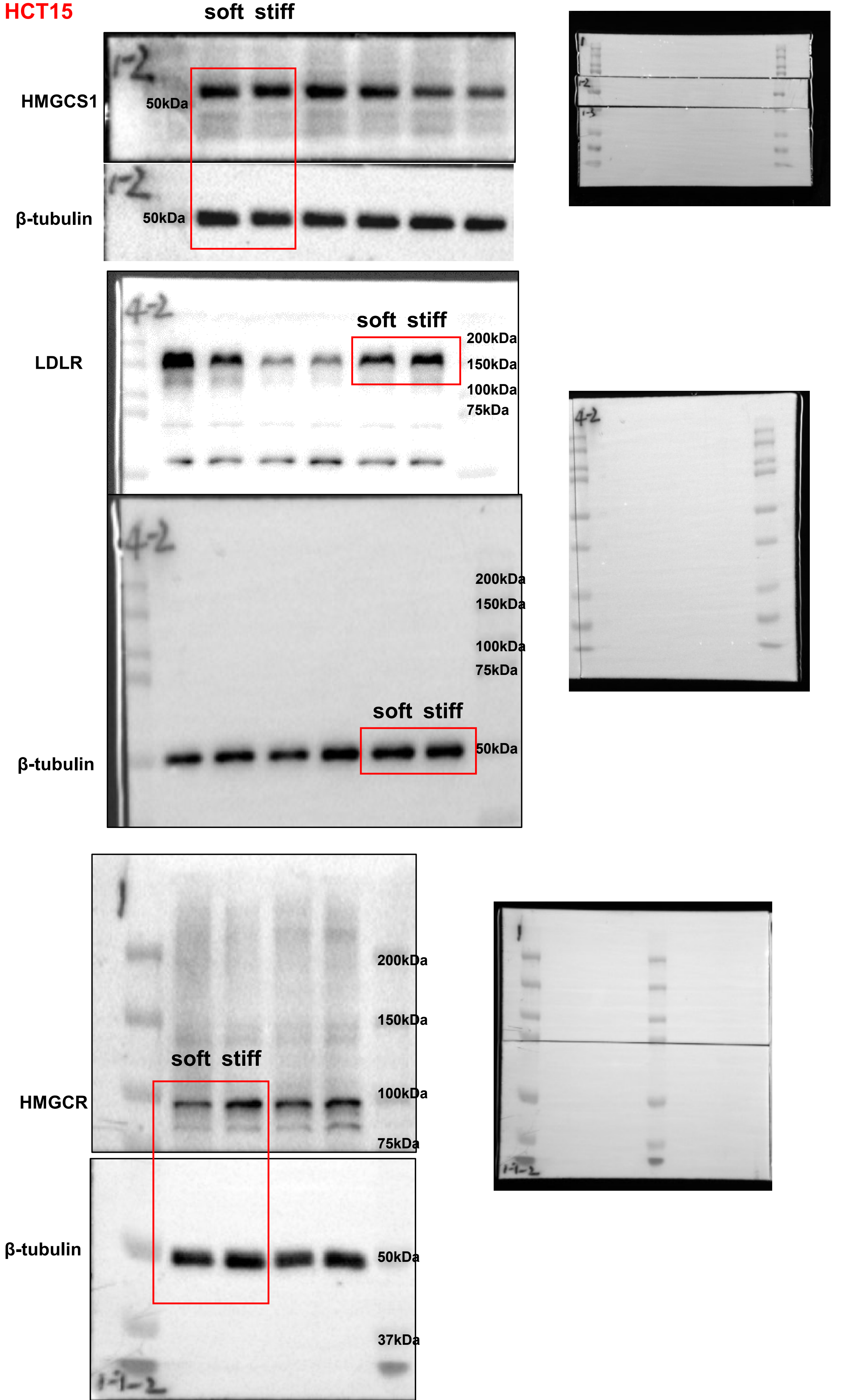

Figure 2 SW480

H

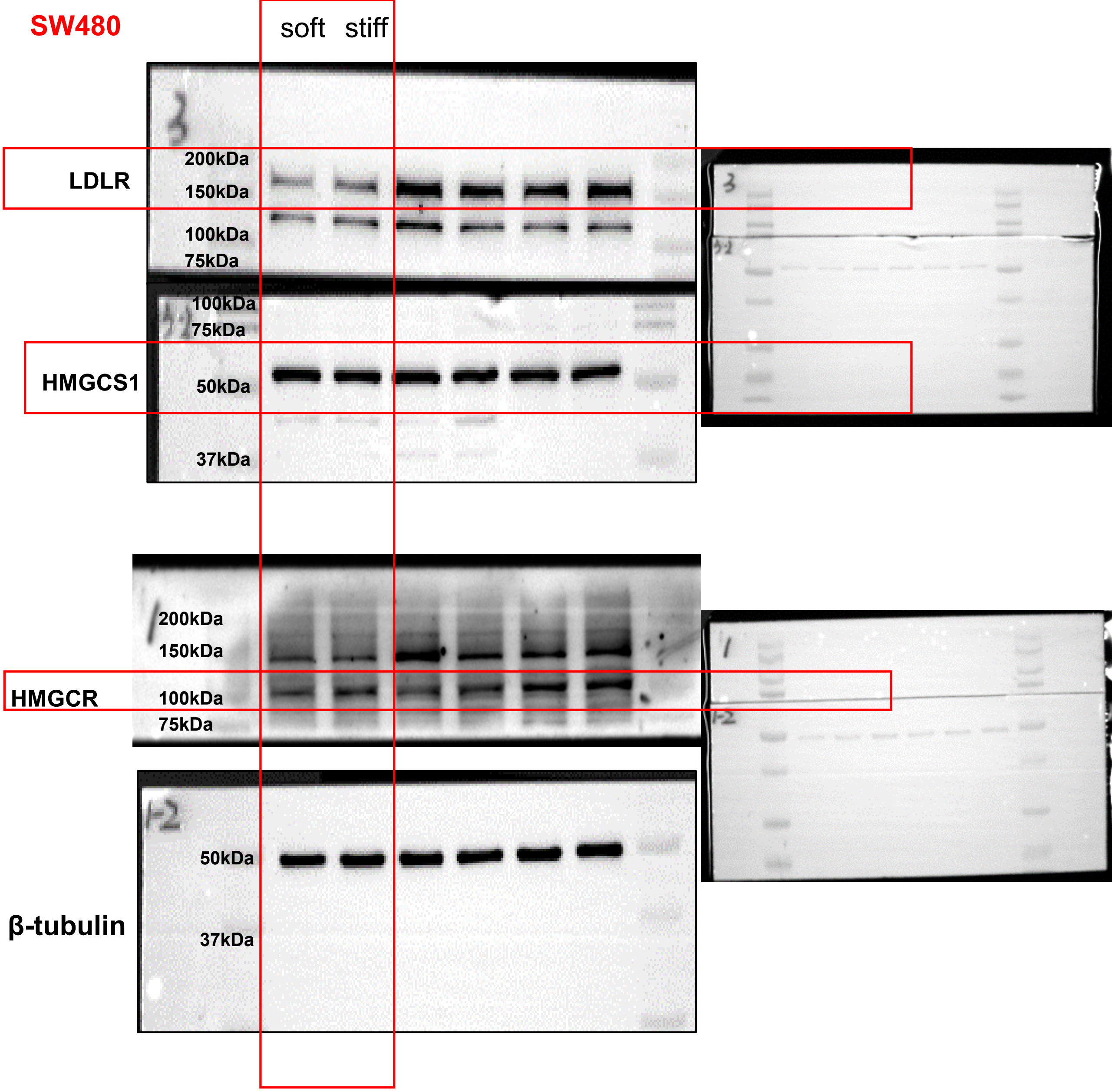

Figure 2 HT29

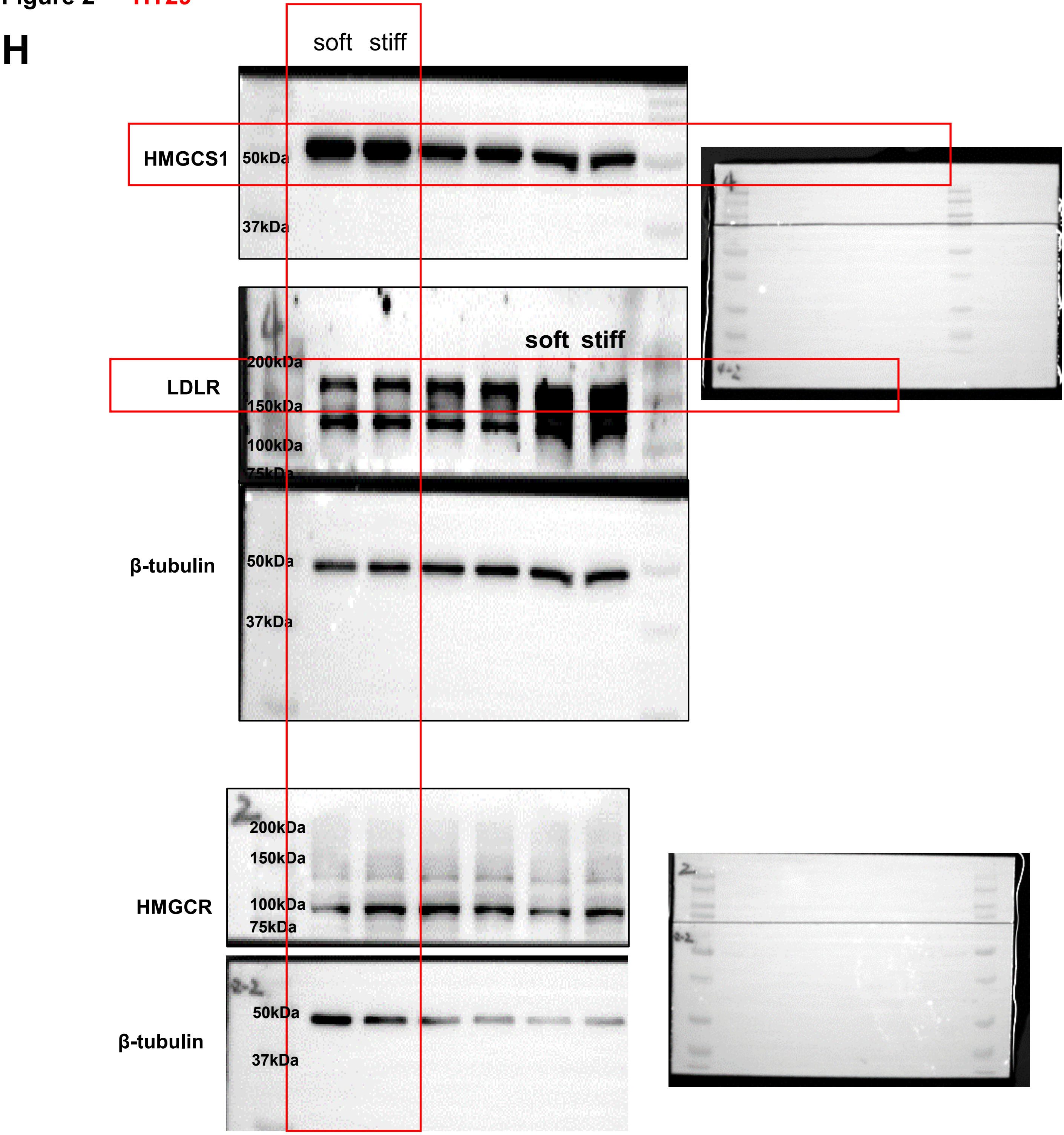

Figure 2

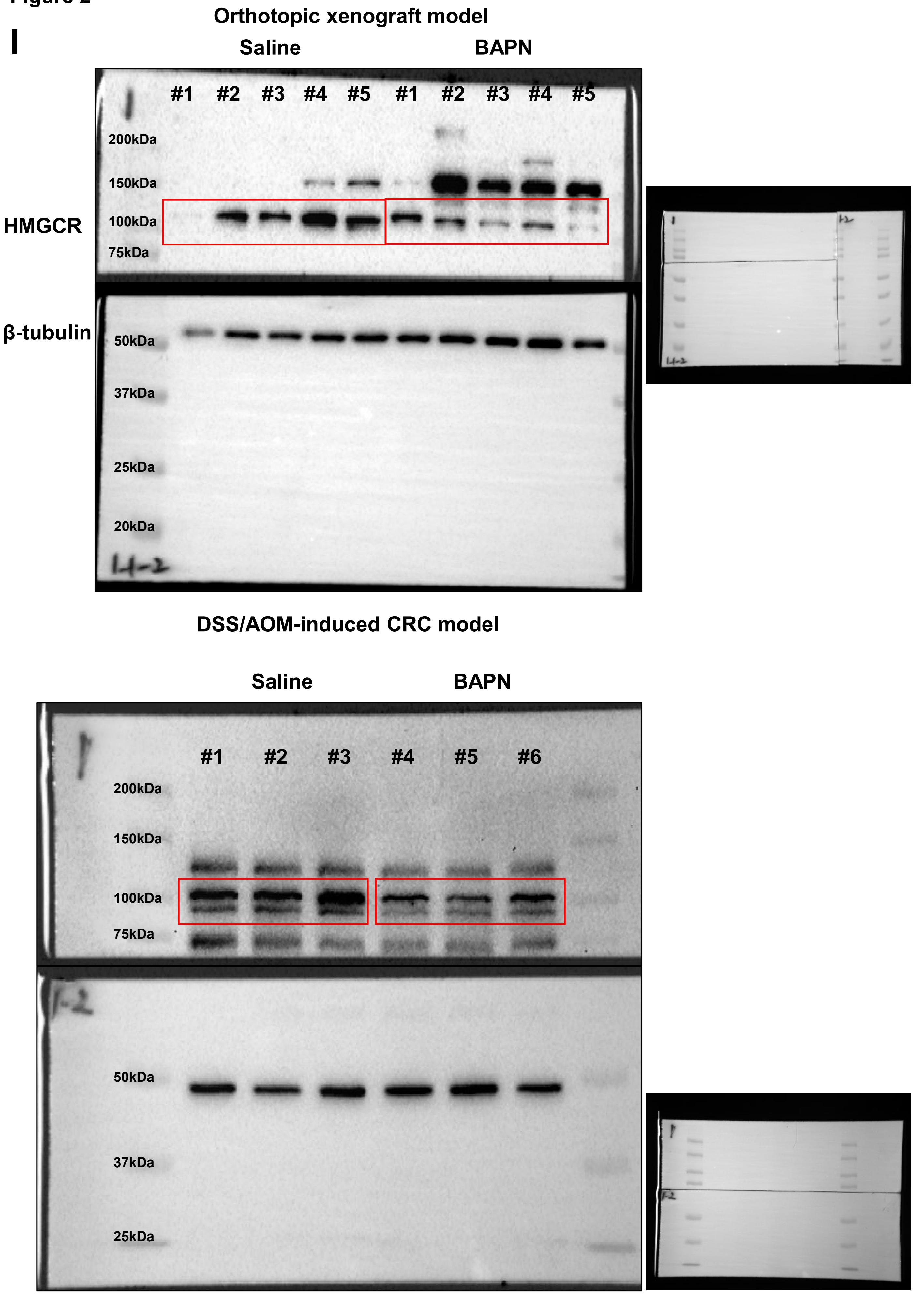
I
Orthotopic xenograft model
Saline
BAPN
#1 #2 #3 #4 #5 #1 #2 #3 #4 #5
200kDa
150kDa
100kDa
75kDa
HMGCR
β-tubulin
50kDa
37kDa
25kDa
20kDa
DSS/AOM-induced CRC model
Saline
BAPN
#1 #2 #3 #4 #5 #6
200kDa
150kDa
100kDa
75kDa
50kDa
37kDa
25kDa

Figure 4

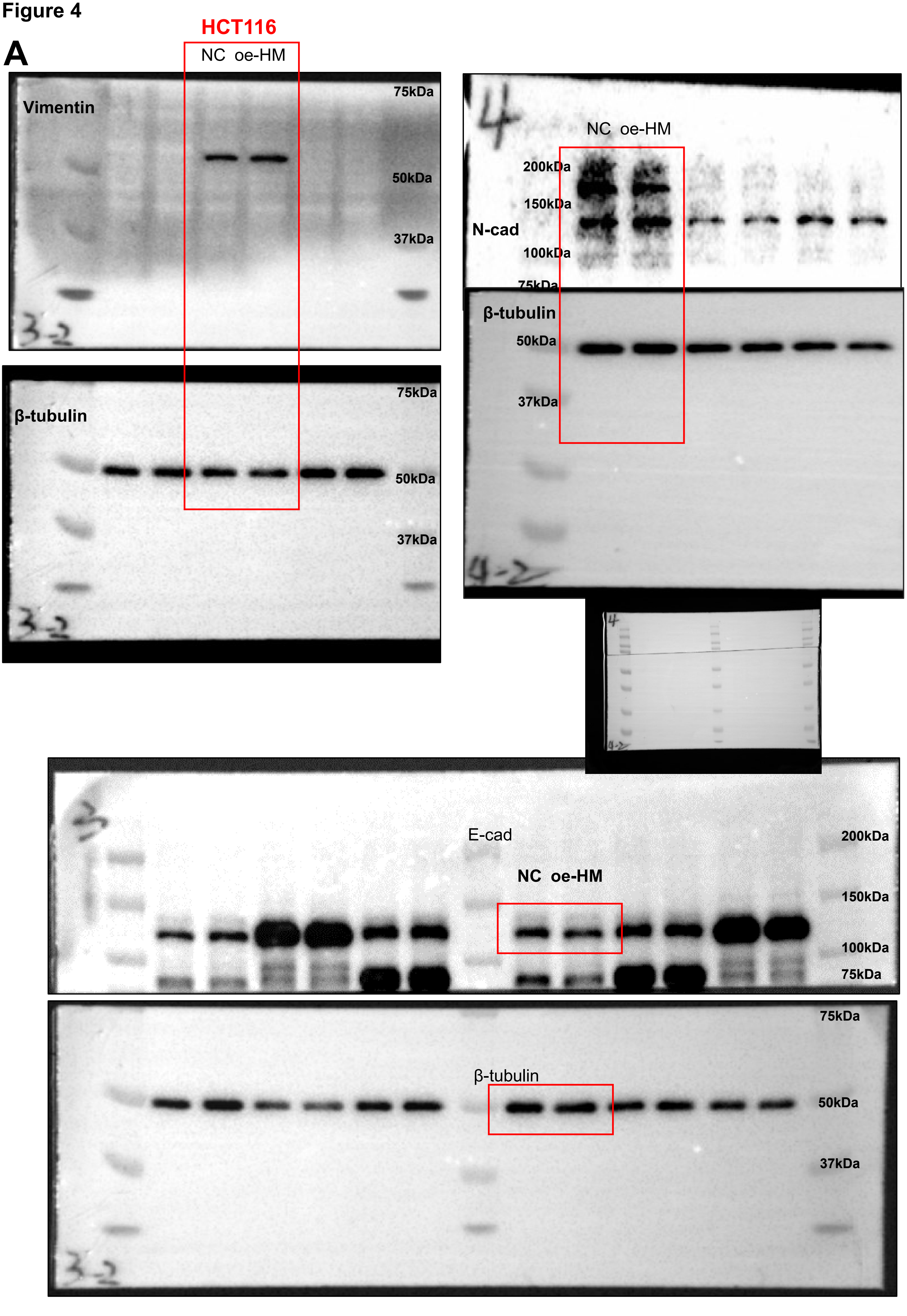
A
HCT116
NC oe-HM
Vimentin
75kDa
50kDa
37kDa
β-tubulin
75kDa
50kDa
37kDa
NC oe-HM
200kDa
150kDa
N-cad
100kDa
75kDa
β-tubulin
50kDa
37kDa
E-cad
NC oe-HM
200kDa
150kDa
100kDa
75kDa
β-tubulin
75kDa
50kDa
37kDa

Figure 4

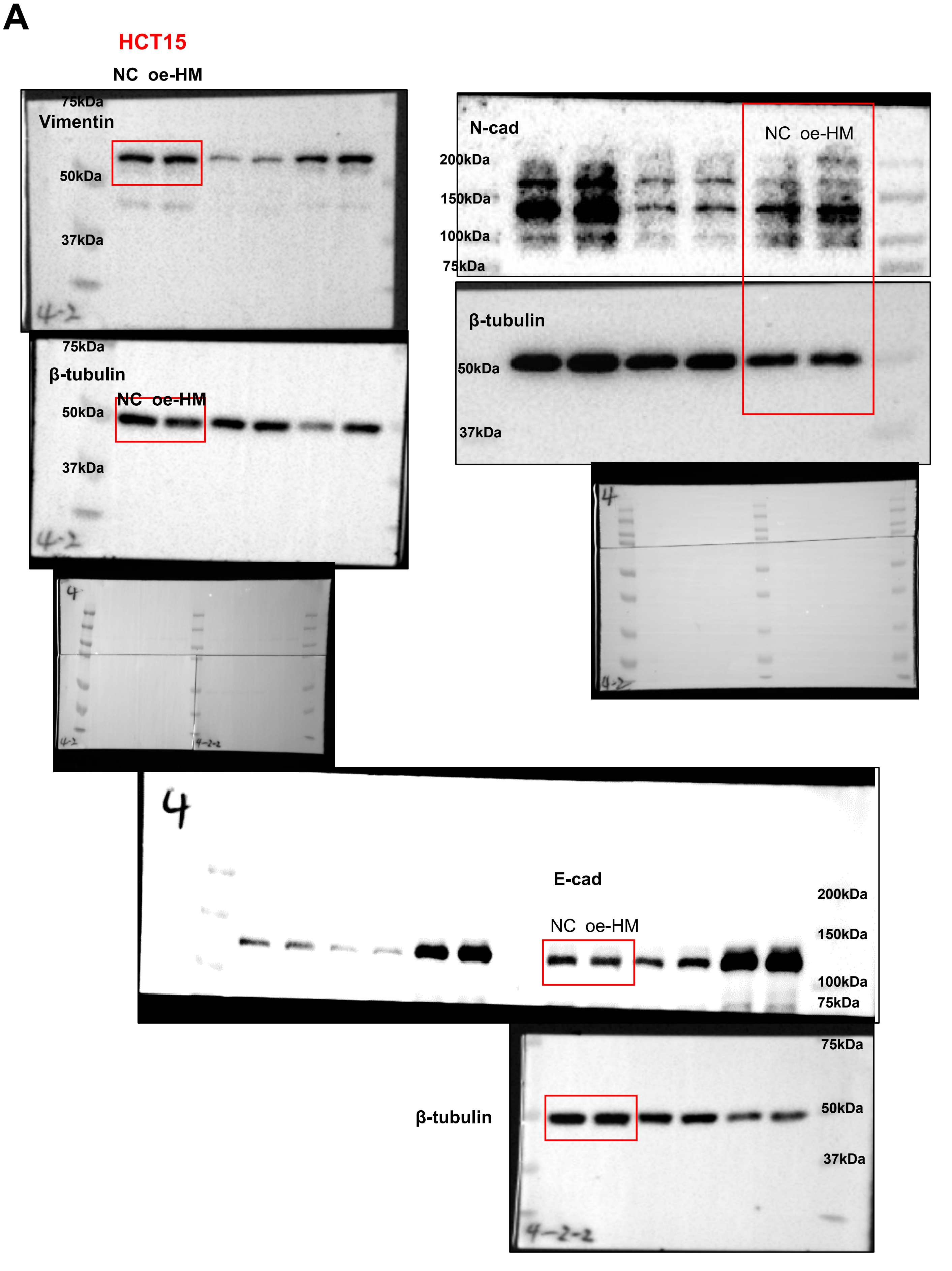
A
HCT15
NC oe-HM
75kDa
Vimentin
50kDa
37kDa
75kDa
β-tubulin
NC oe-HM
50kDa
37kDa
N-cad
NC oe-HM
200kDa
150kDa
100kDa
75kDa
β-tubulin
50kDa
37kDa
E-cad
NC oe-HM
200kDa
150kDa
100kDa
75kDa
β-tubulin
75kDa
50kDa
37kDa

Figure 4

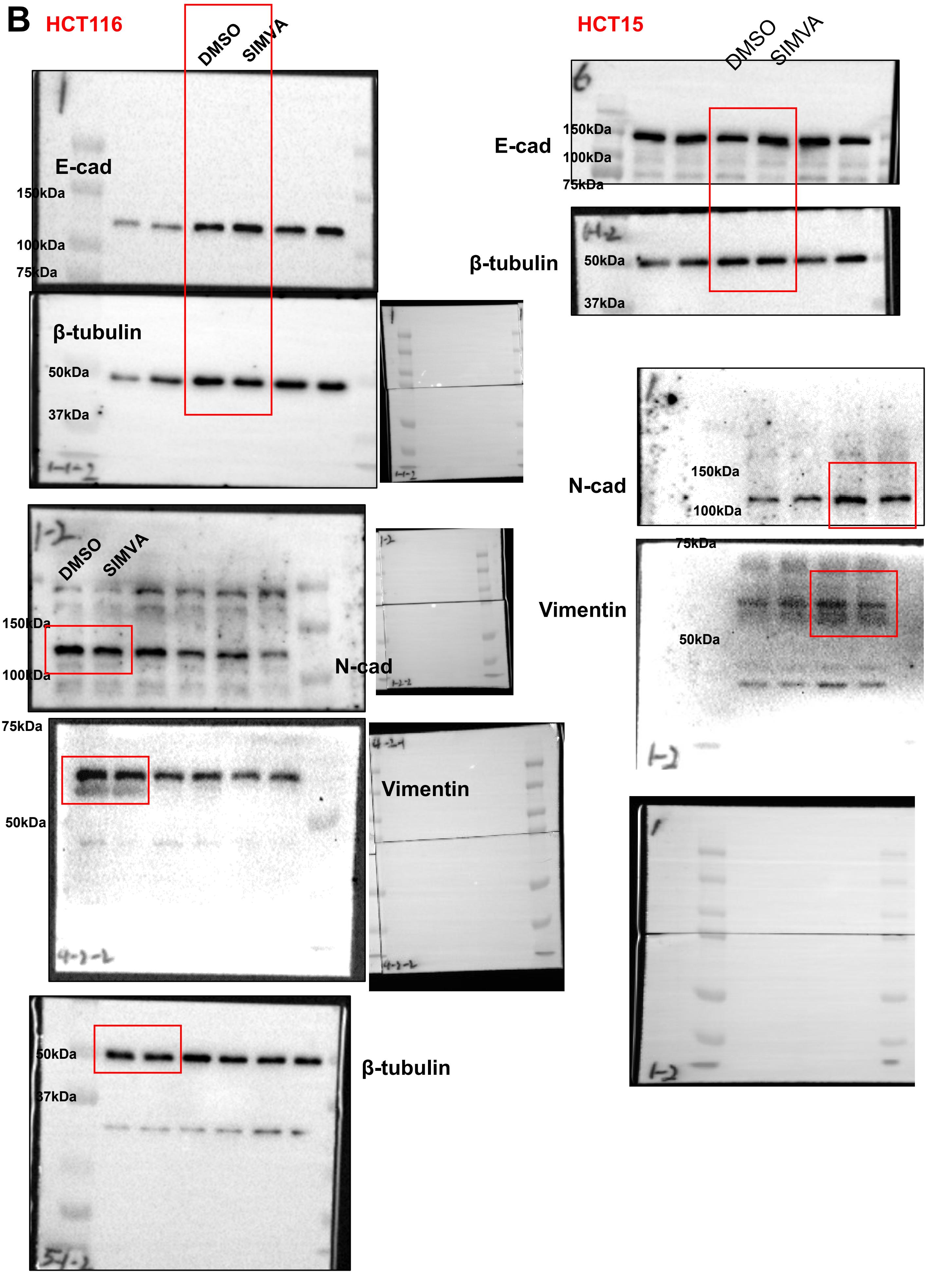

B
HCT116
DMSO
SIMVA
E-cad
150kDa
100kDa
75kDa
β-tubulin
50kDa
37kDa
DMSO
SIMVA
150kDa
100kDa
N-cad
75kDa
50kDa
Vimentin
50kDa
37kDa
β-tubulin
HCT15
DMSO
SIMVA
150kDa
E-cad
100kDa
75kDa
β-tubulin
50kDa
37kDa
N-cad
150kDa
100kDa
75kDa
Vimentin
50kDa

Figure 4

C

HCT116

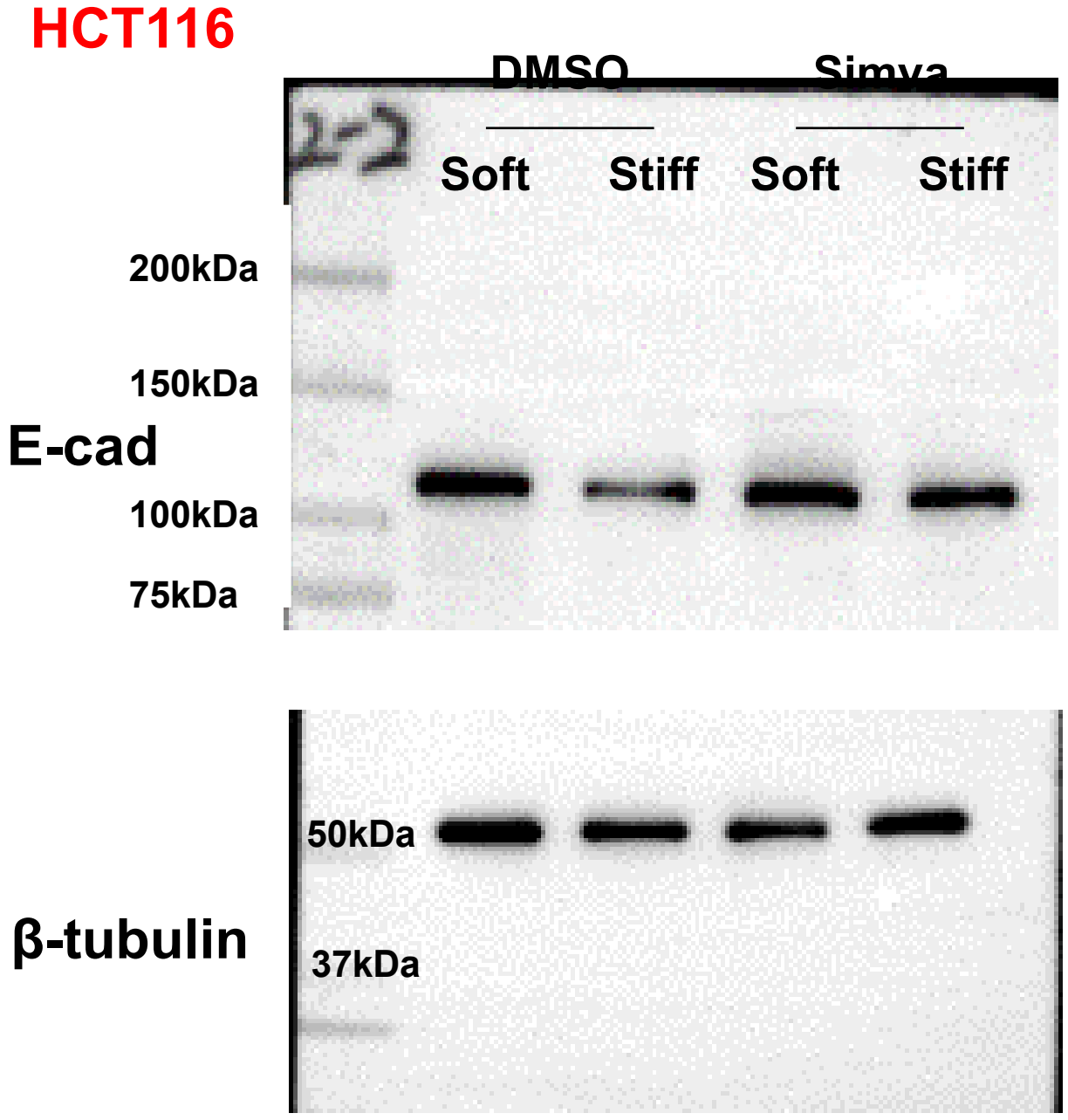

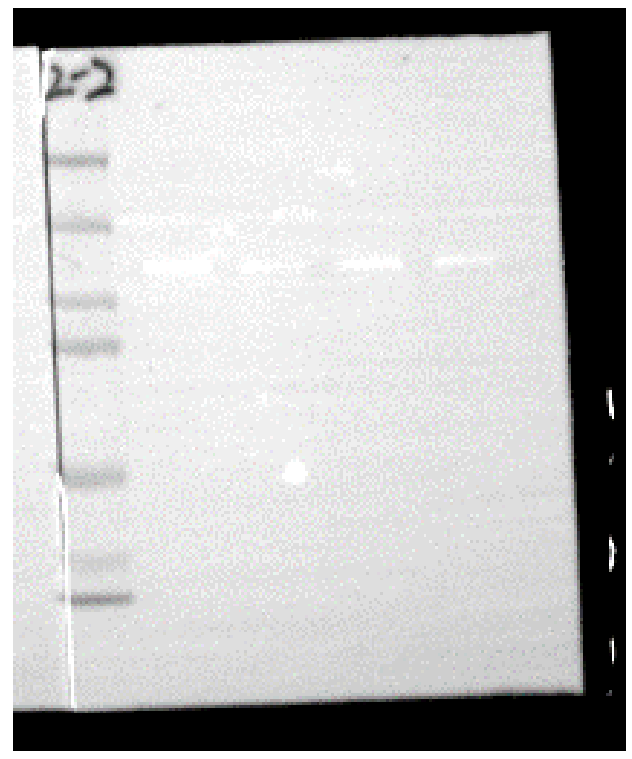

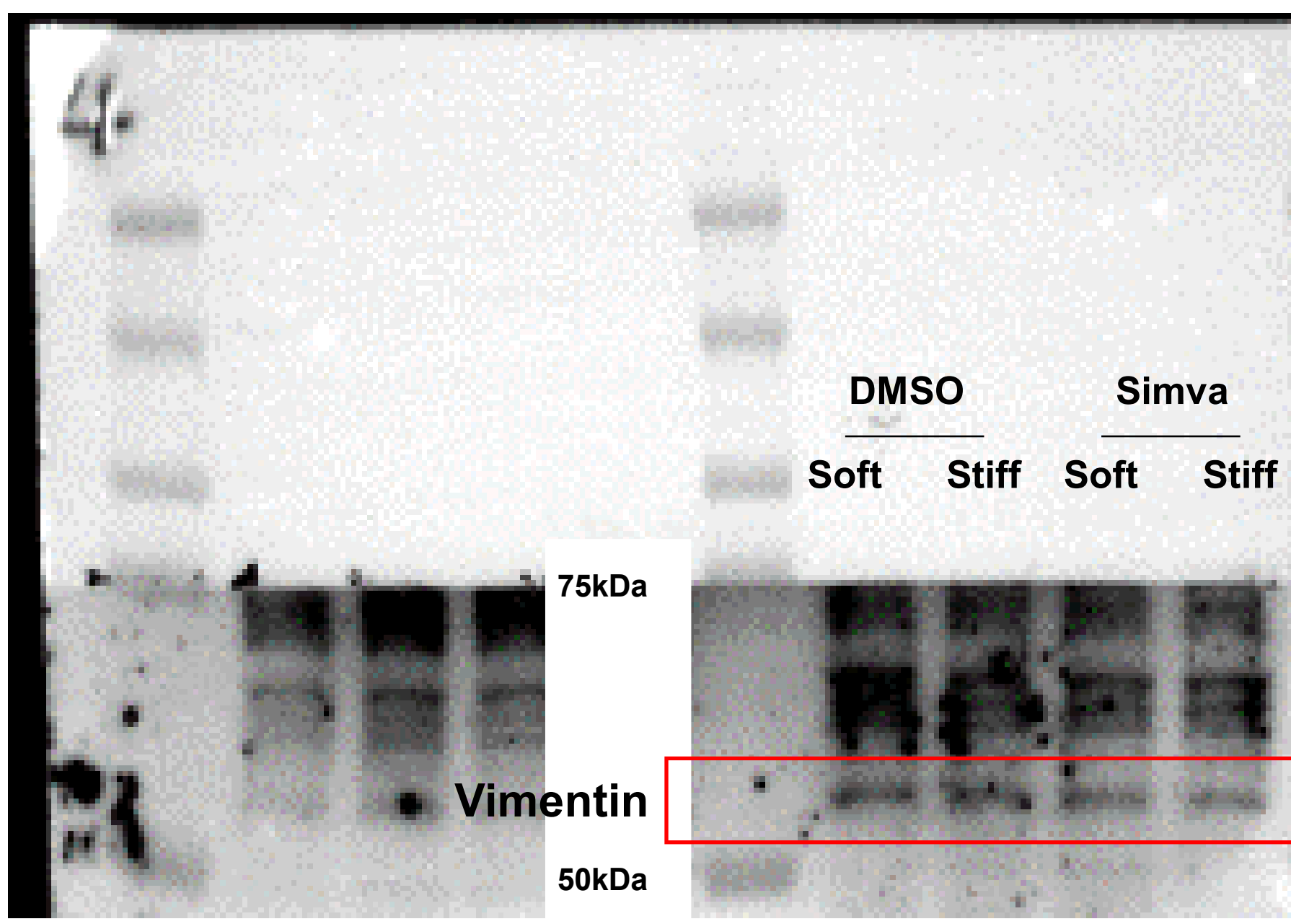

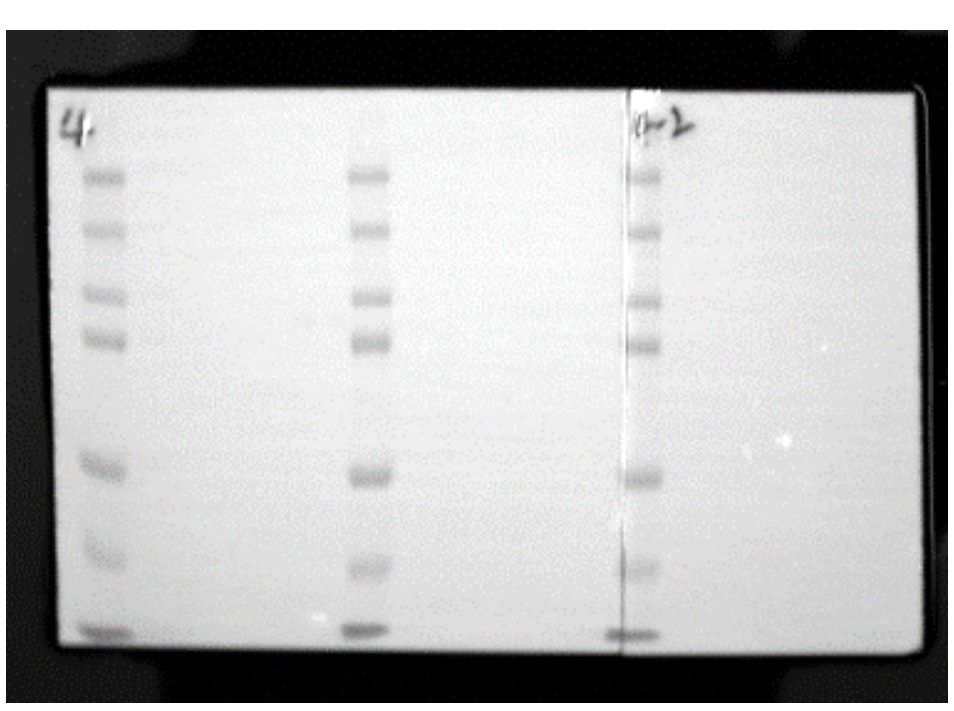

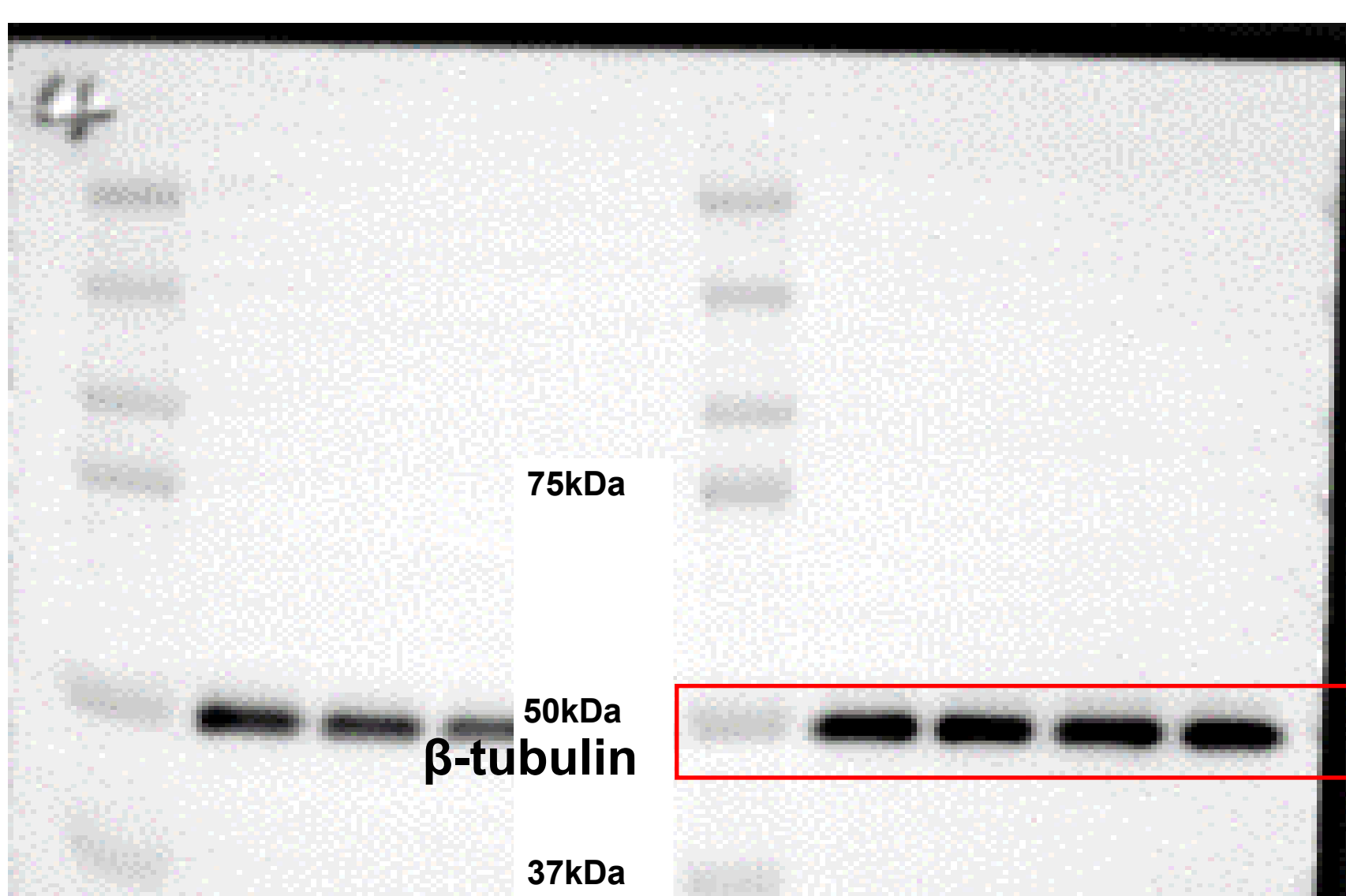

Figure 4

C

HCT15

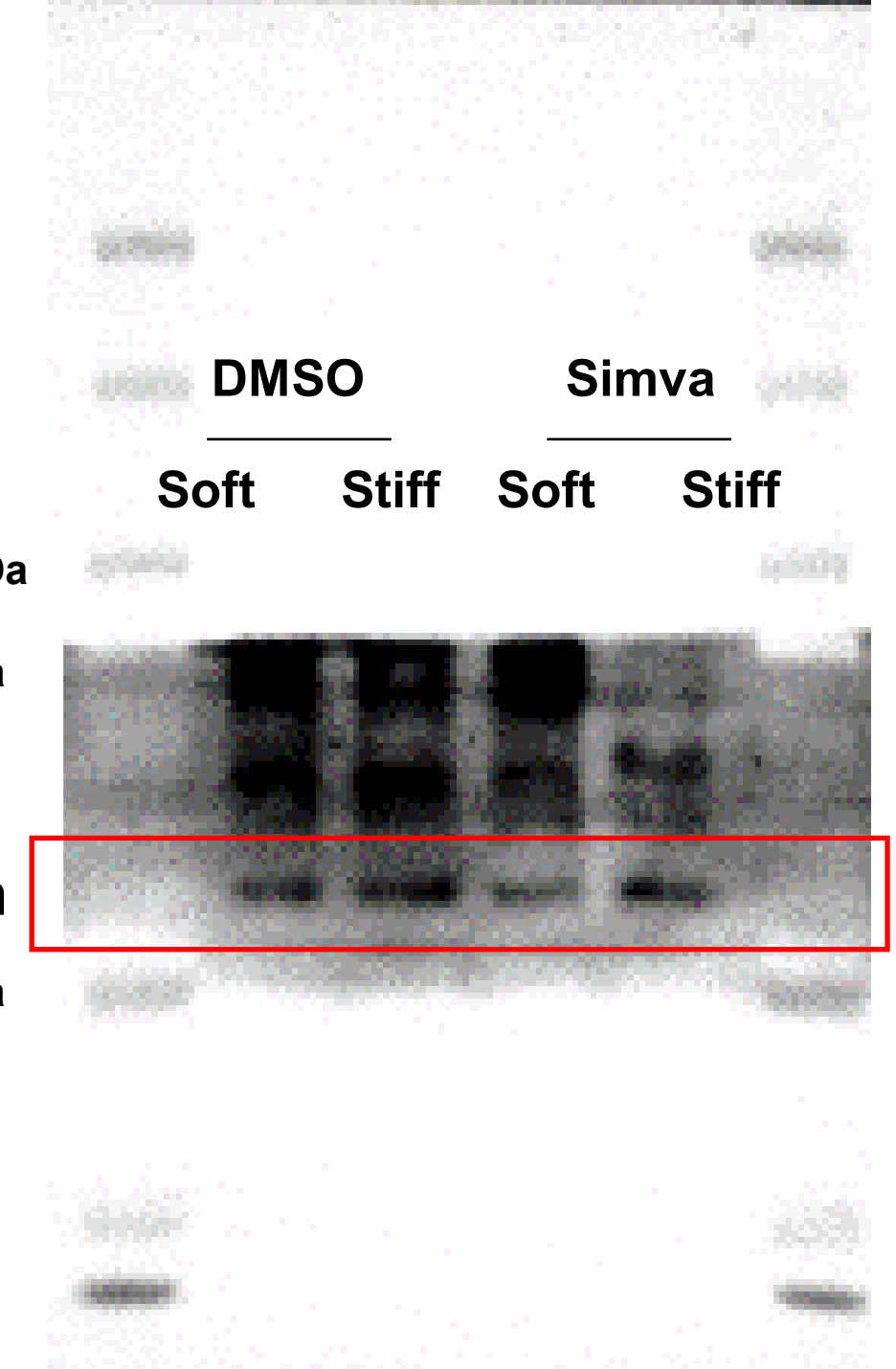

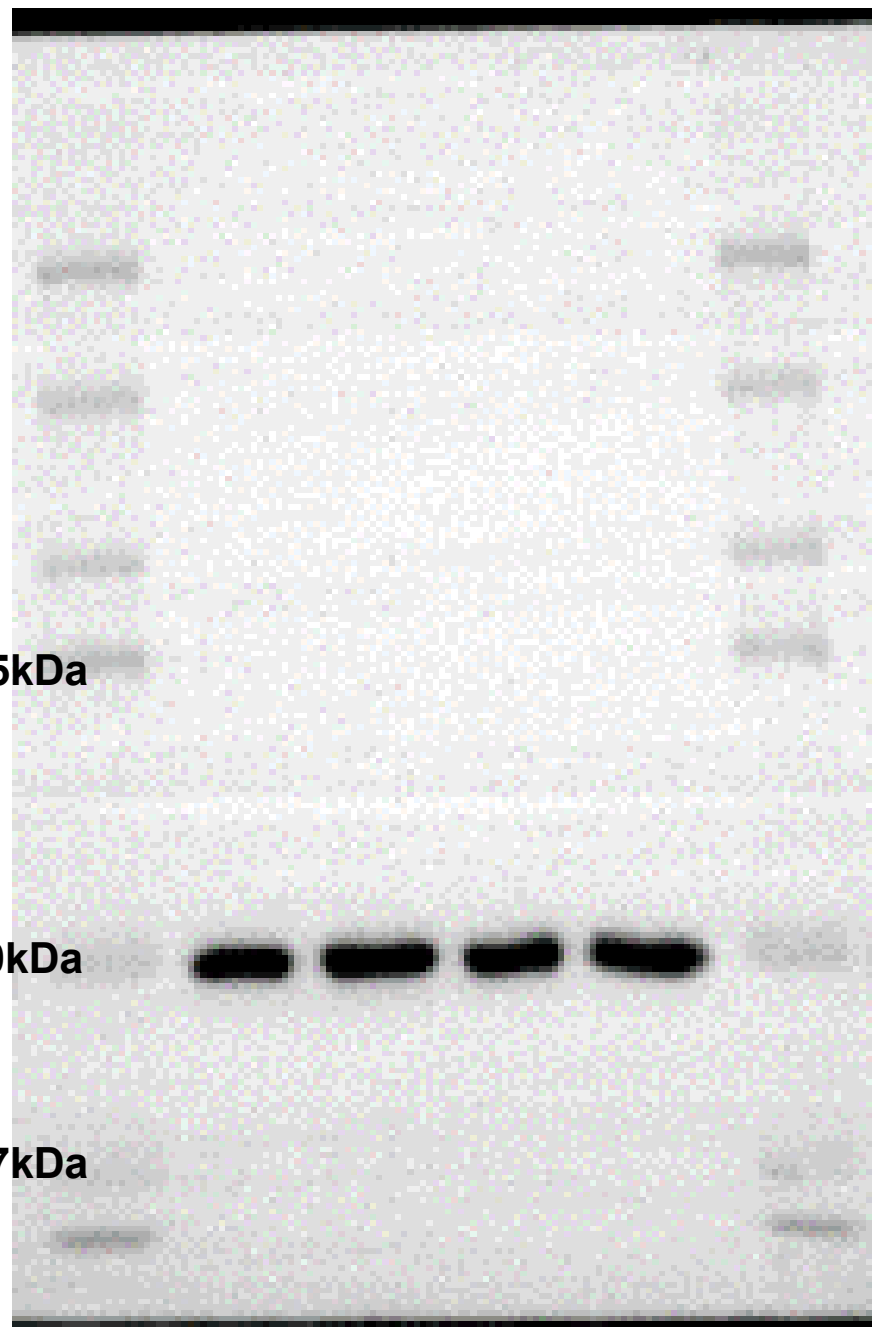

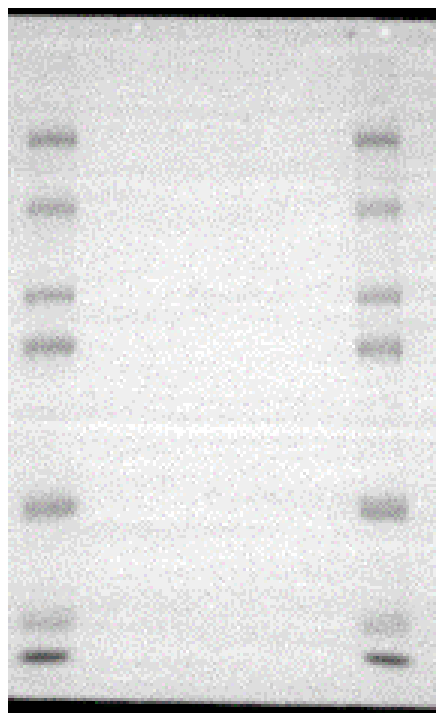

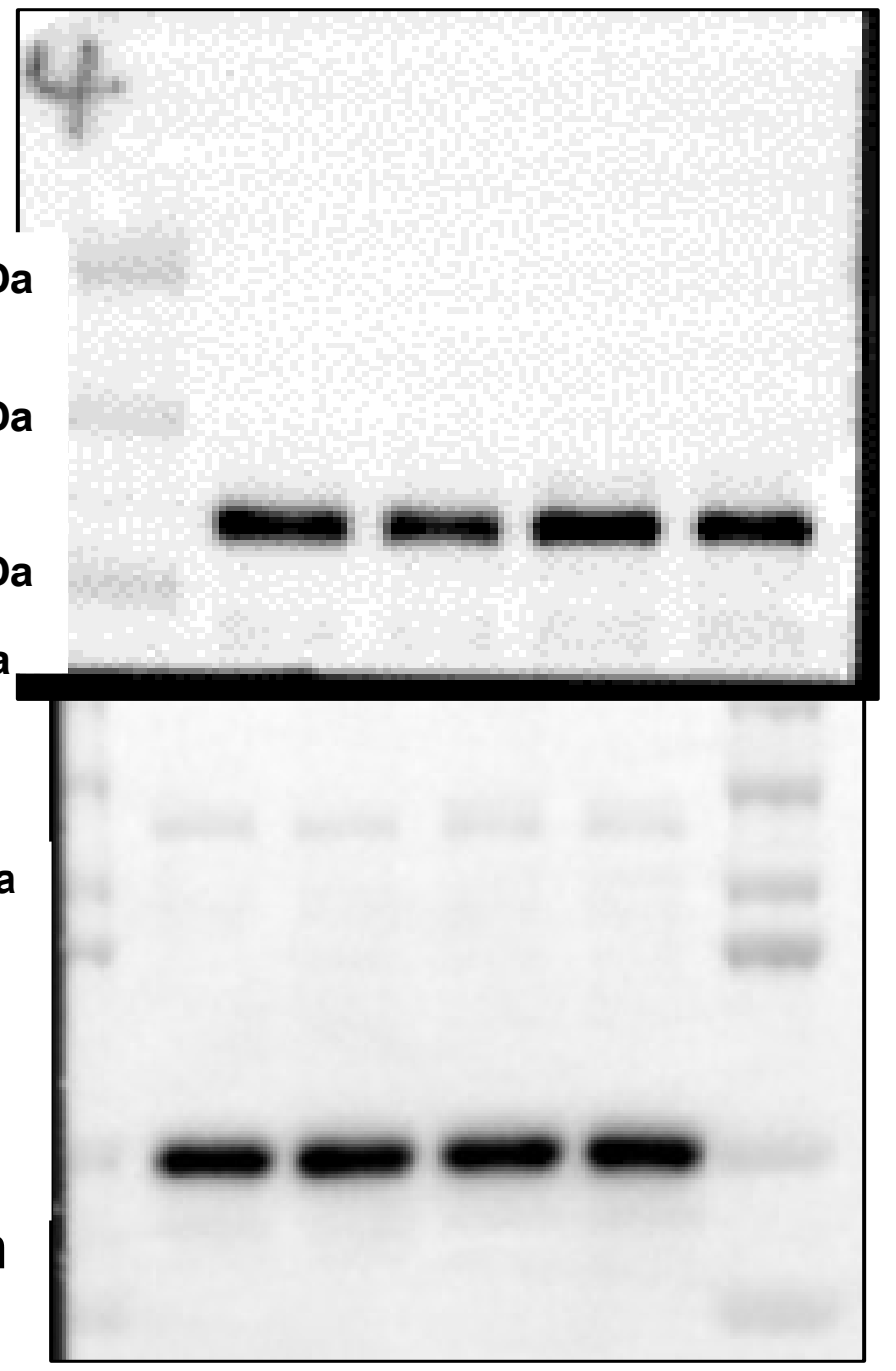

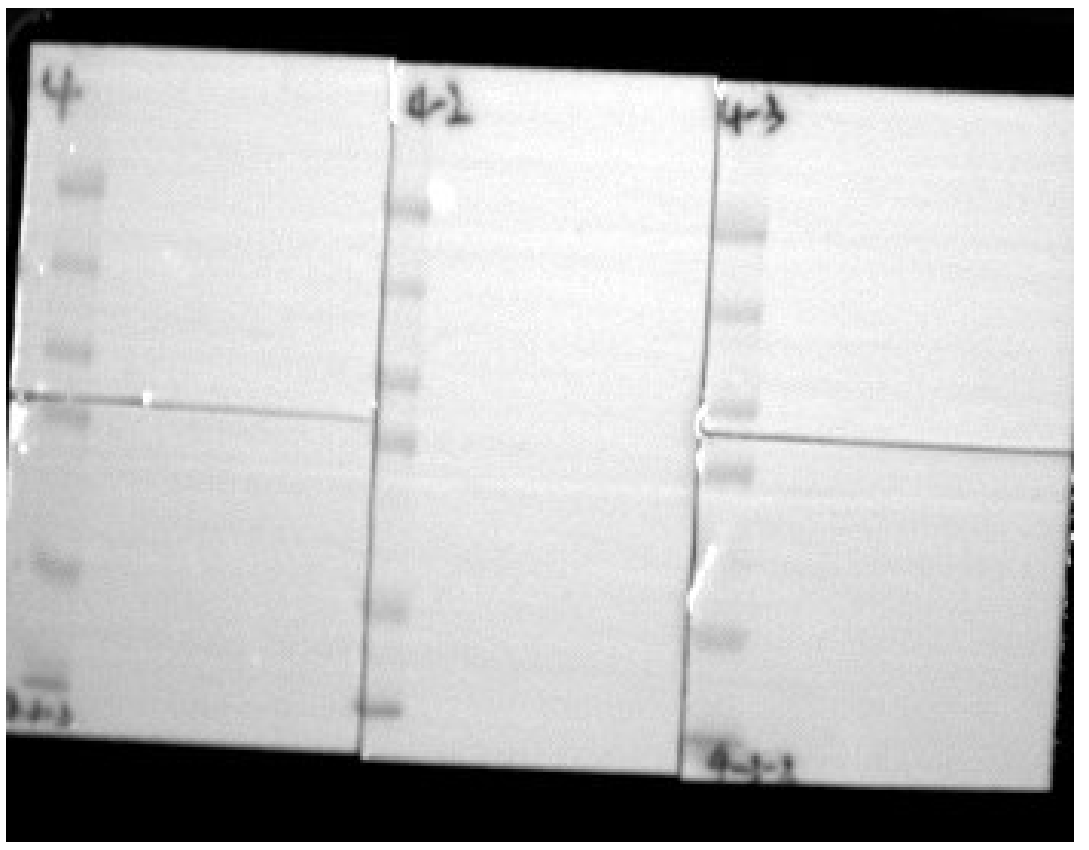

Figure 4

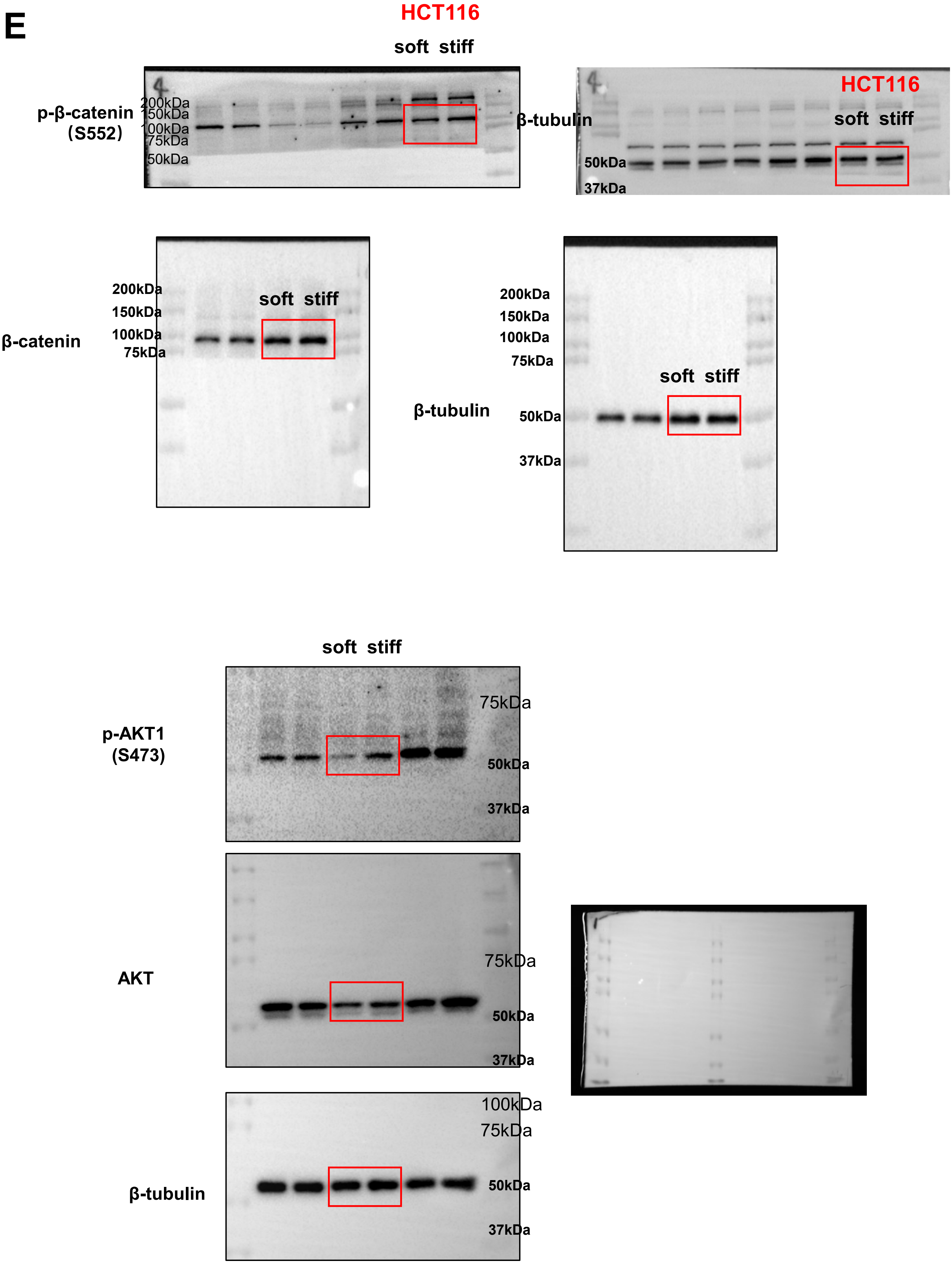
E
HCT116
soft stiff
p-β-catenin (S552)
200kDa
150kDa
100kDa
75kDa
50kDa
β-tubulin
HCT116
soft stiff
50kDa
37kDa
β-catenin
200kDa
150kDa
100kDa
75kDa
soft stiff
β-tubulin
200kDa
150kDa
100kDa
75kDa
soft stiff
50kDa
37kDa
soft stiff
p-AKT1 (S473)
75kDa
50kDa
37kDa
AKT
75kDa
50kDa
37kDa
β-tubulin
100kDa
75kDa
50kDa
37kDa

Figure 4

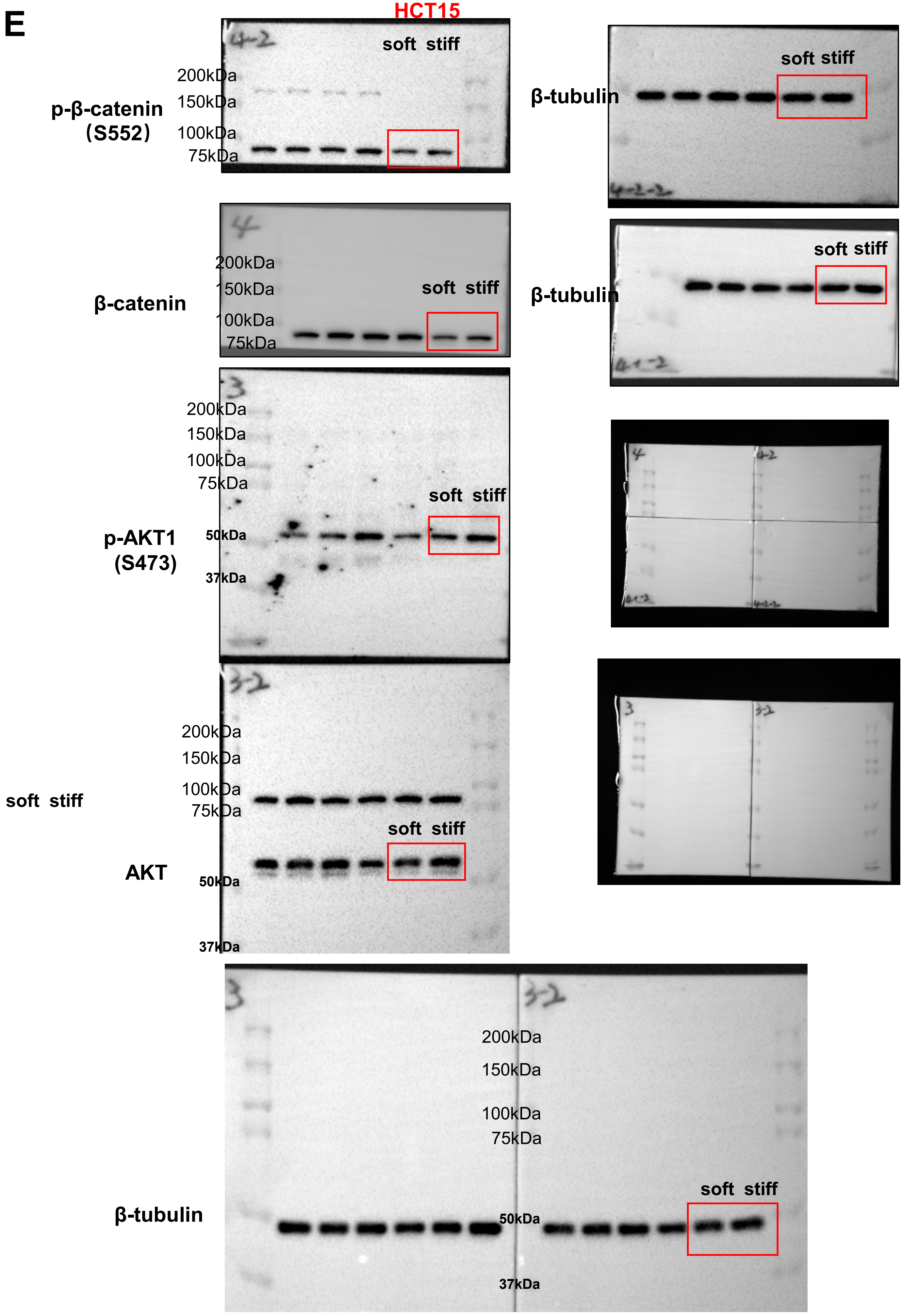

E
HCT15
soft stiff
200kDa
150kDa
100kDa
75kDa
p-β-catenin (S552)
β-tubulin
soft stiff
β-catenin
soft stiff
200kDa
150kDa
100kDa
75kDa
β-tubulin
soft stiff
p-AKT1 (S473)
200kDa
150kDa
100kDa
75kDa
50kDa
37kDa
soft stiff
soft stiff
AKT
200kDa
150kDa
100kDa
75kDa
50kDa
37kDa
soft stiff
β-tubulin
200kDa
150kDa
100kDa
75kDa
50kDa
37kDa
soft stiff

Figure 4

F

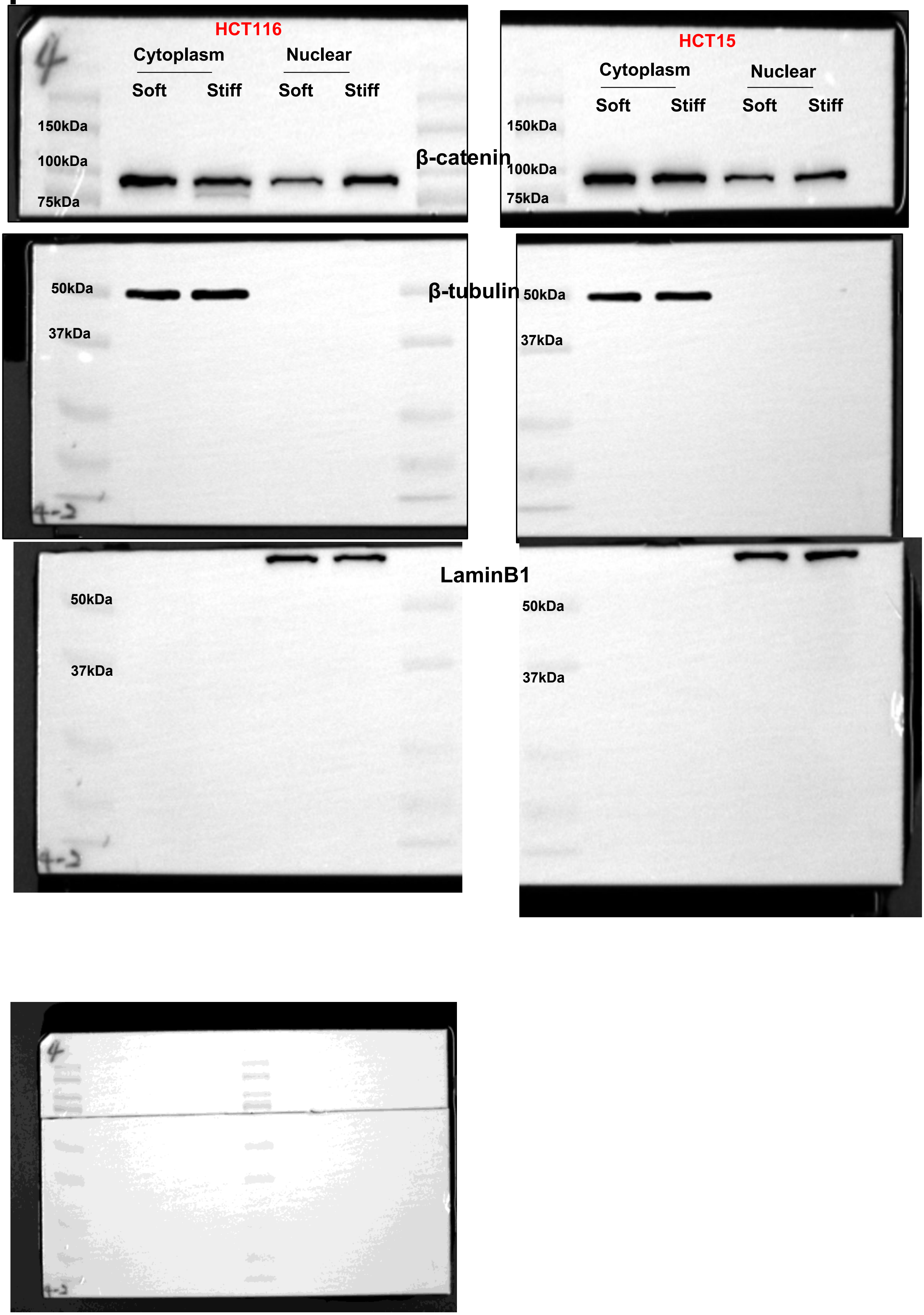
HCT116
Cytoplasm
Nuclear
Soft
Stiff
Soft
Stiff
150kDa
100kDa
75kDa
β-catenin
HCT15
Cytoplasm
Nuclear
Soft
Stiff
Soft
Stiff
150kDa
100kDa
75kDa
50kDa
37kDa
β-tubulin
50kDa
37kDa
LaminB1
50kDa
37kDa
50kDa
37kDa

Figure 4

G

HCT116

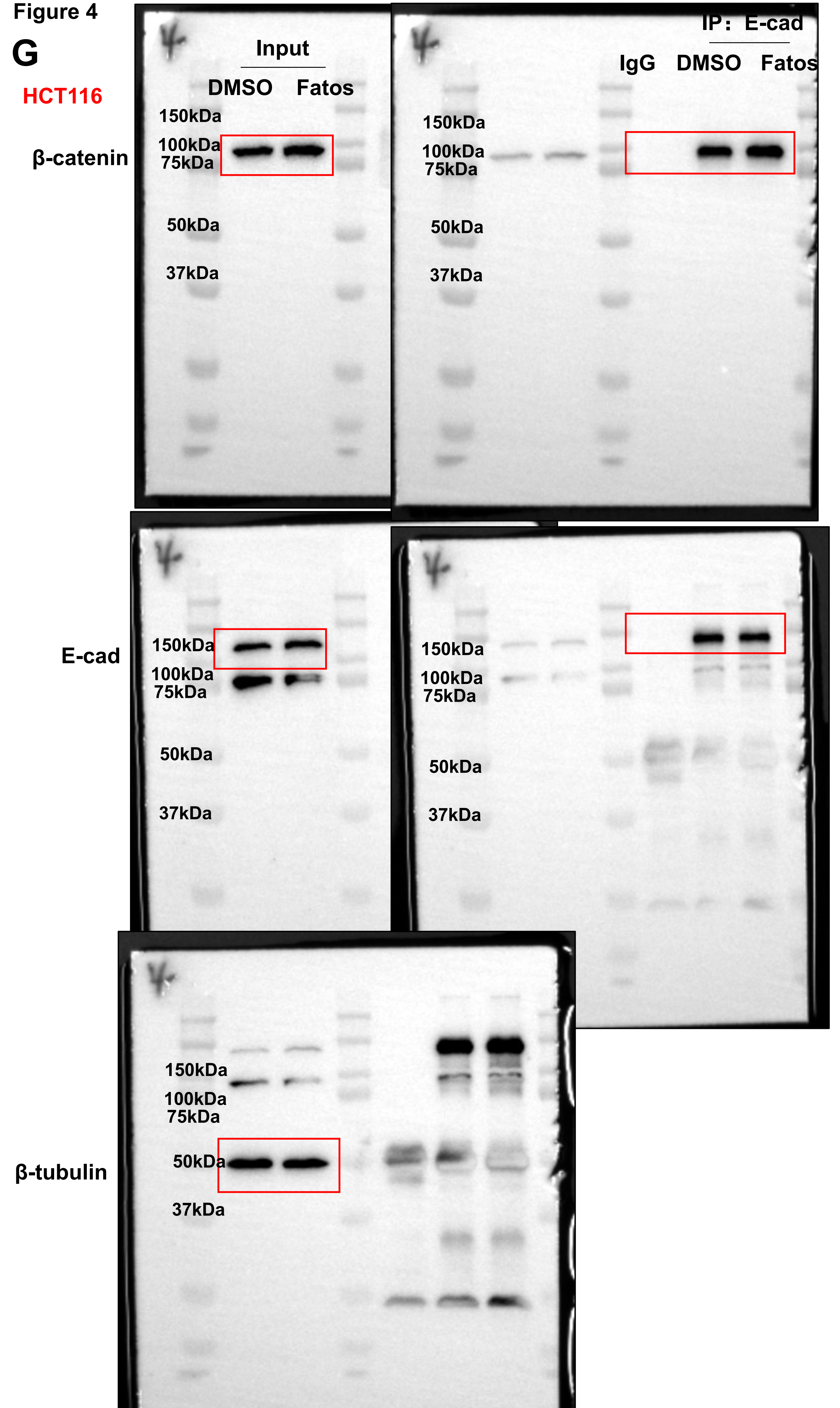

Figure 4

G

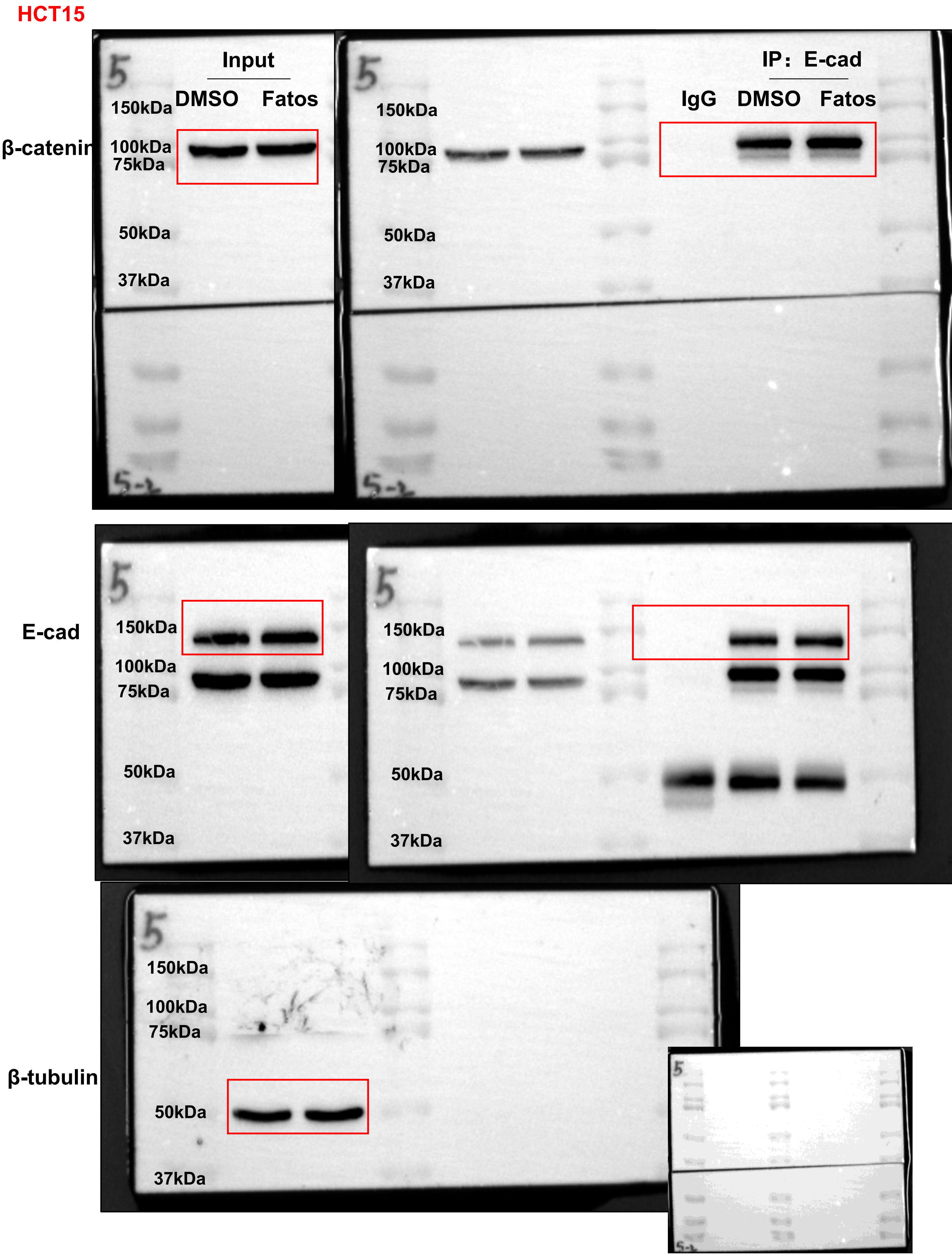

HCT15
β-catenin
Input
DMSO
Fatos
150kDa
100kDa
75kDa
50kDa
37kDa
IP：E-cad
IgG
DMSO
Fatos
E-cad
β-tubulin

Figure 4

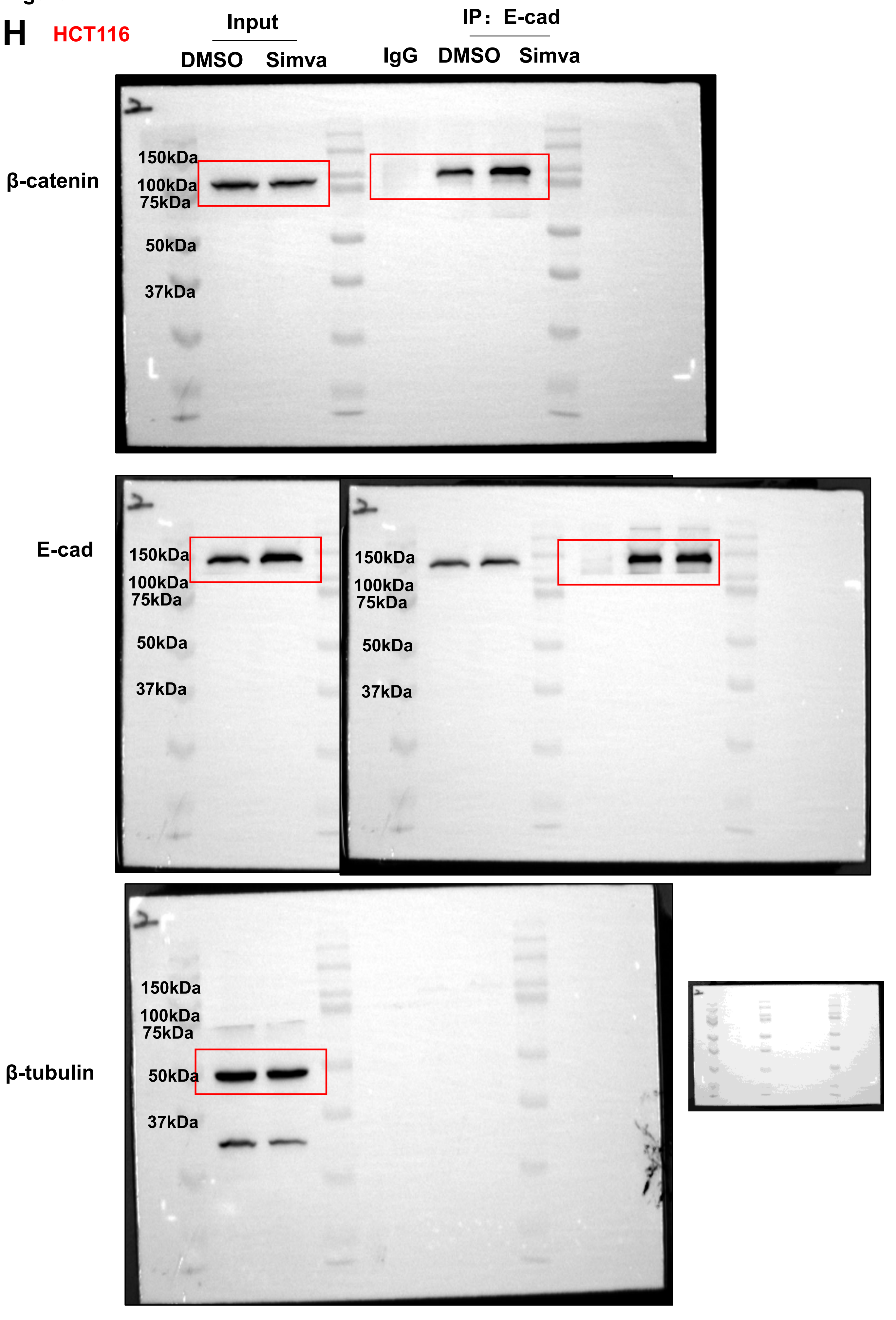
H
HCT116
Input
IP: E-cad
DMSO
Simva
IgG
DMSO
Simva
β-catenin
E-cad
β-tubulin
150kDa
100kDa
75kDa
50kDa
37kDa

Figure 4

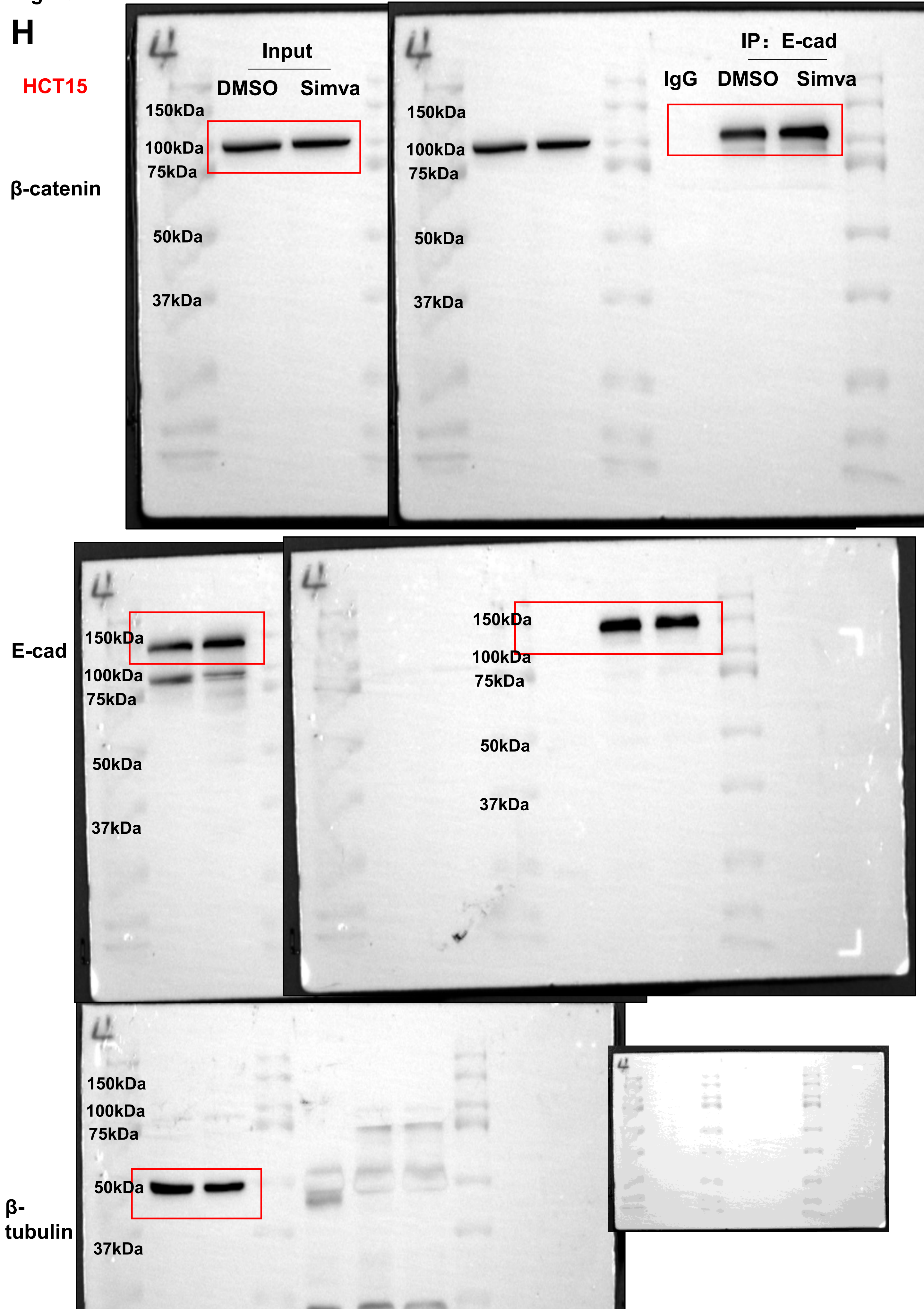
H
HCT15
Input
DMSO
Simva
IP：E-cad
IgG
DMSO
Simva
150kDa
100kDa
75kDa
50kDa
37kDa
β-catenin
E-cad
β-
tubulin

Figure 4

I

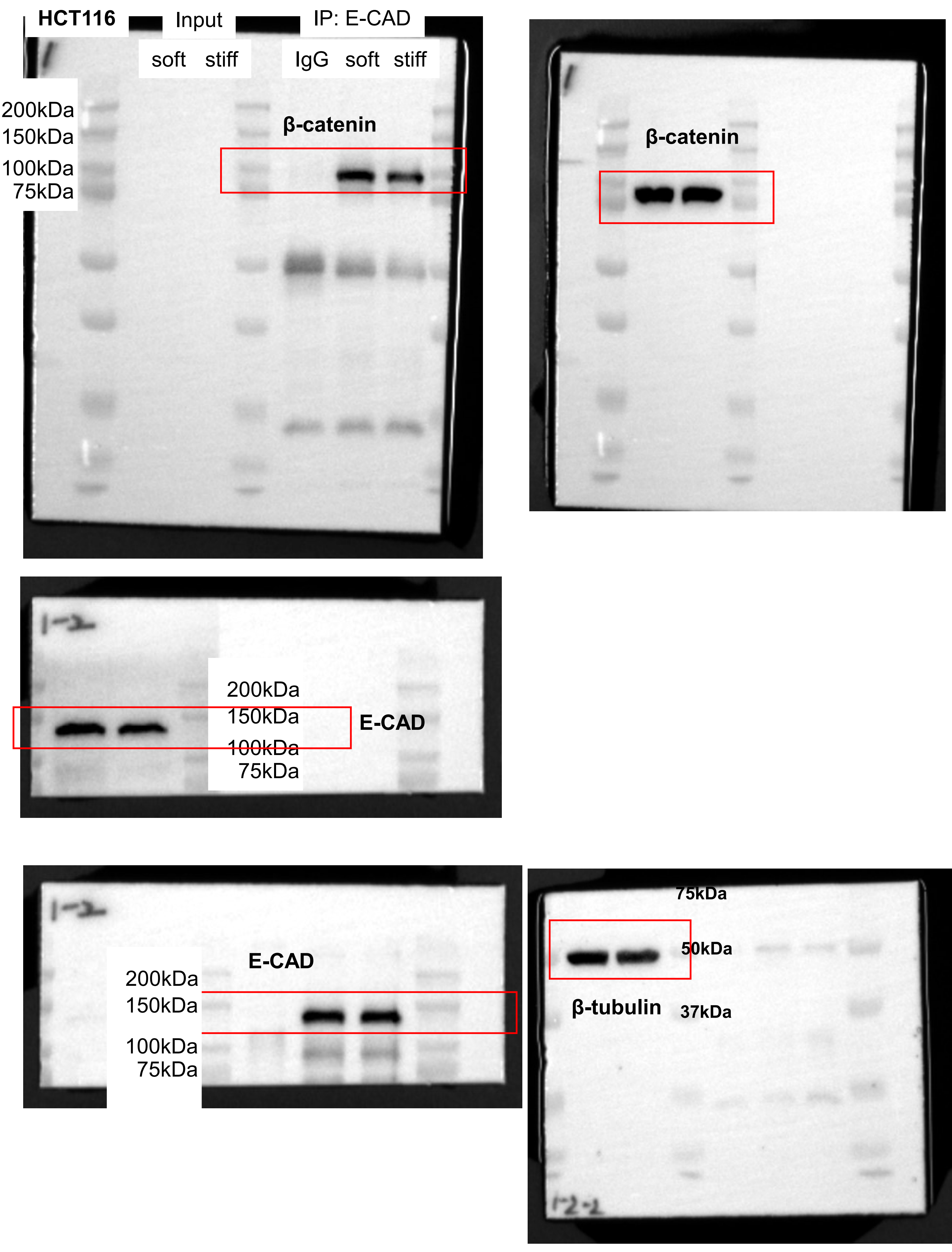
HCT116
Input
IP: E-CAD
soft
stiff
IgG
soft
stiff
200kDa
150kDa
100kDa
75kDa
β-catenin
β-catenin
200kDa
150kDa
100kDa
75kDa
E-CAD
200kDa
150kDa
100kDa
75kDa
E-CAD
75kDa
50kDa
β-tubulin
37kDa

Figure 4

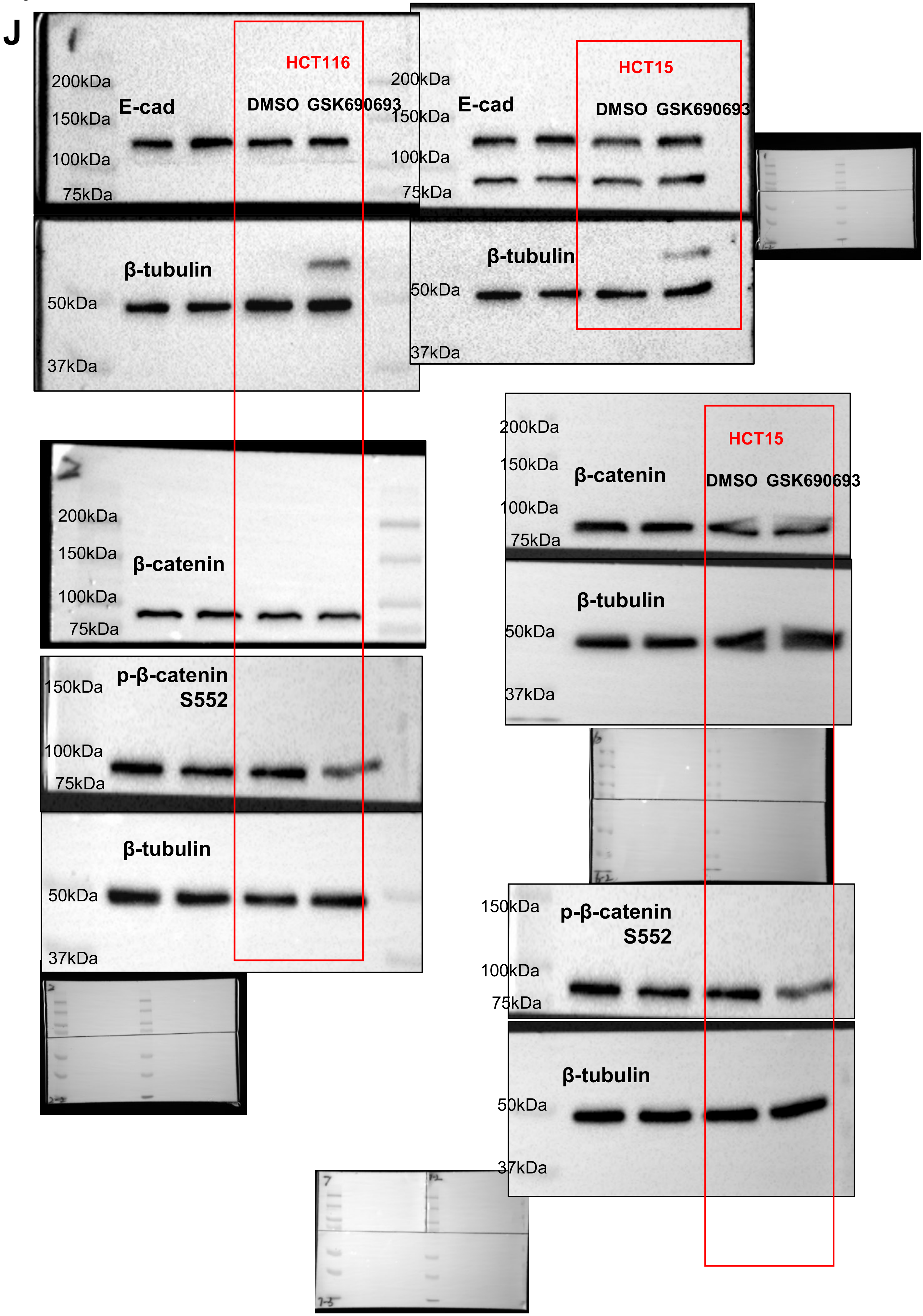
J
HCT116
200kDa
150kDa
100kDa
75kDa
E-cad
DMSO GSK690693
β-tubulin
50kDa
37kDa
HCT15
E-cad
DMSO GSK690693
β-tubulin
β-catenin
p-β-catenin S552

Figure 4

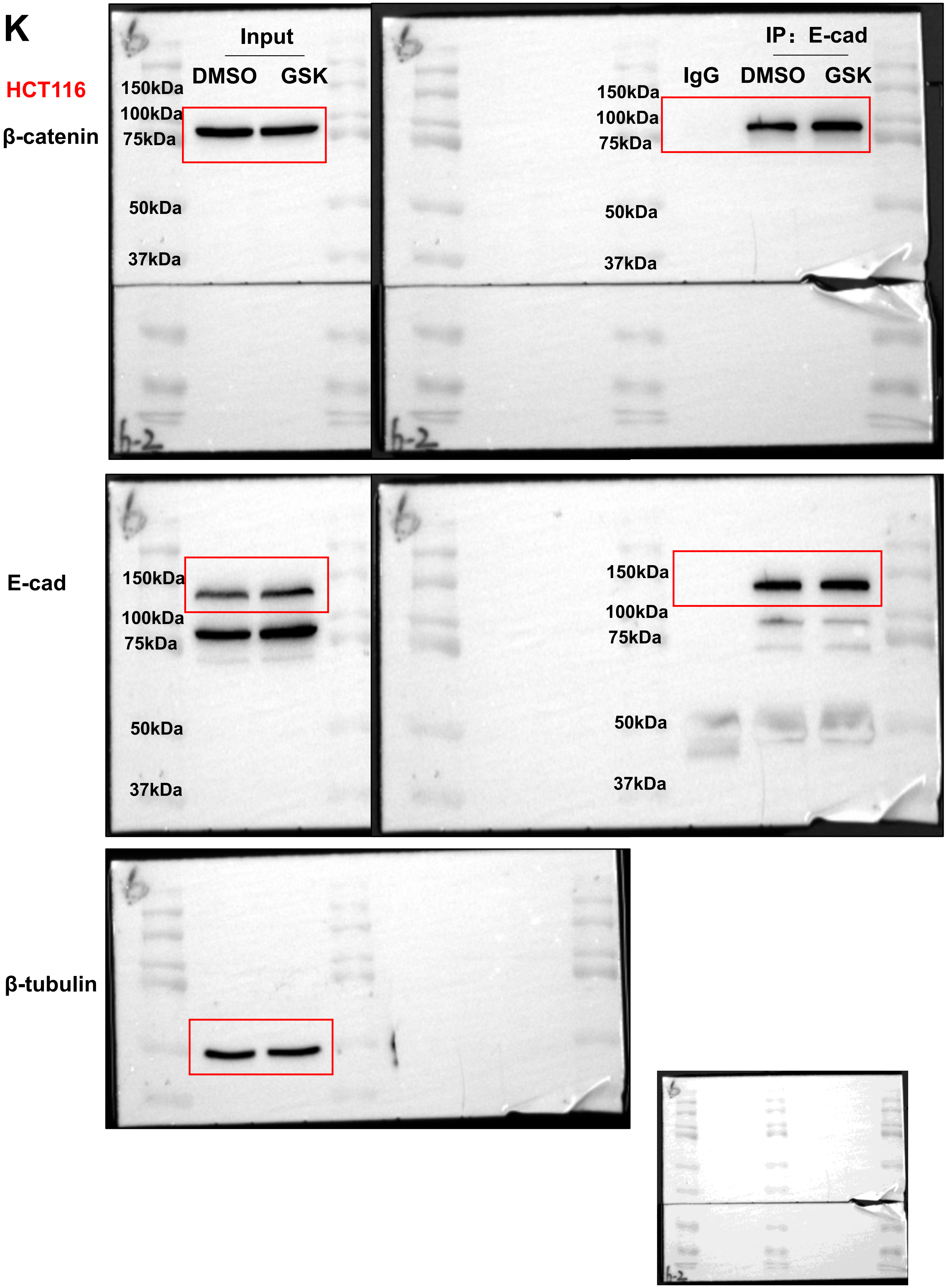
K
HCT116
β-catenin
Input
DMSO
GSK
IP：E-cad
IgG
DMSO
GSK
150kDa
100kDa
75kDa
50kDa
37kDa
E-cad
150kDa
100kDa
75kDa
50kDa
37kDa
β-tubulin

Figure 4 HCT15

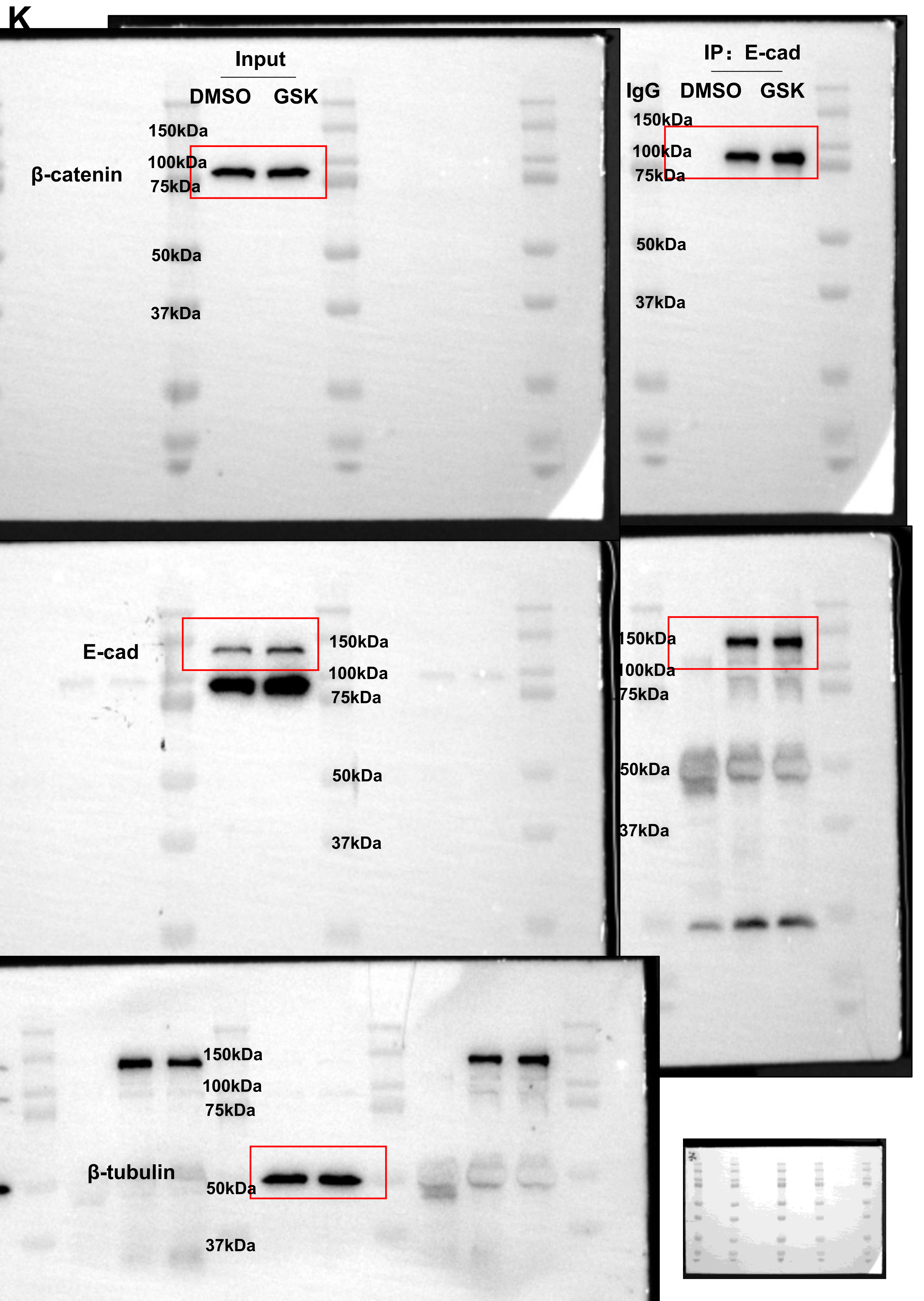

Figure 4

L

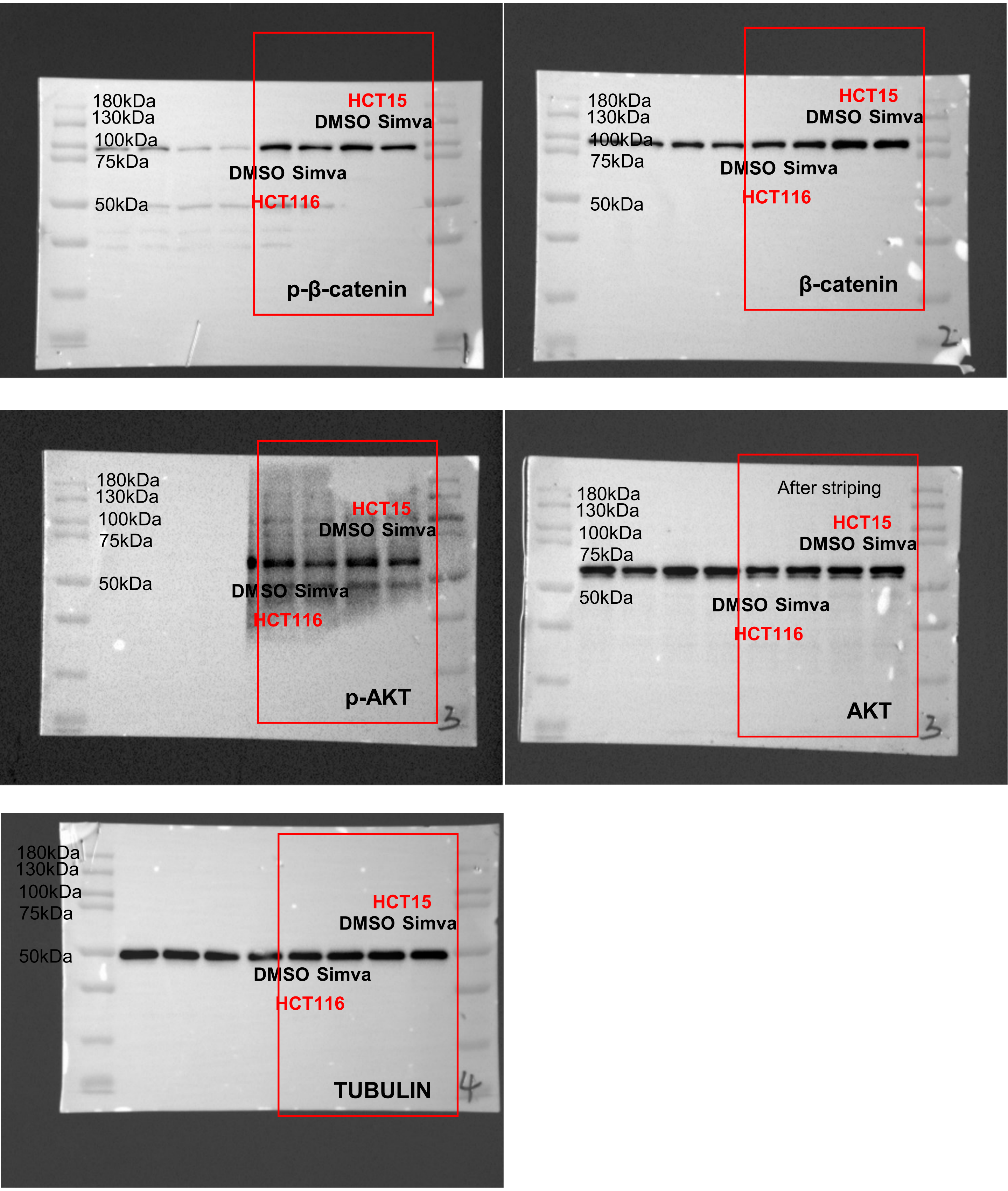
180kDa
130kDa
100kDa
75kDa
50kDa
HCT15
DMSO Simva
DMSO Simva
HCT116
p-β-catenin
180kDa
130kDa
100kDa
75kDa
50kDa
HCT15
DMSO Simva
DMSO Simva
HCT116
β-catenin
180kDa
130kDa
100kDa
75kDa
50kDa
HCT15
DMSO Simva
DMSO Simva
HCT116
p-AKT
After striping
180kDa
130kDa
100kDa
75kDa
50kDa
HCT15
DMSO Simva
DMSO Simva
HCT116
AKT
180kDa
130kDa
100kDa
75kDa
50kDa
HCT15
DMSO Simva
DMSO Simva
HCT116
TUBULIN

Figure 4

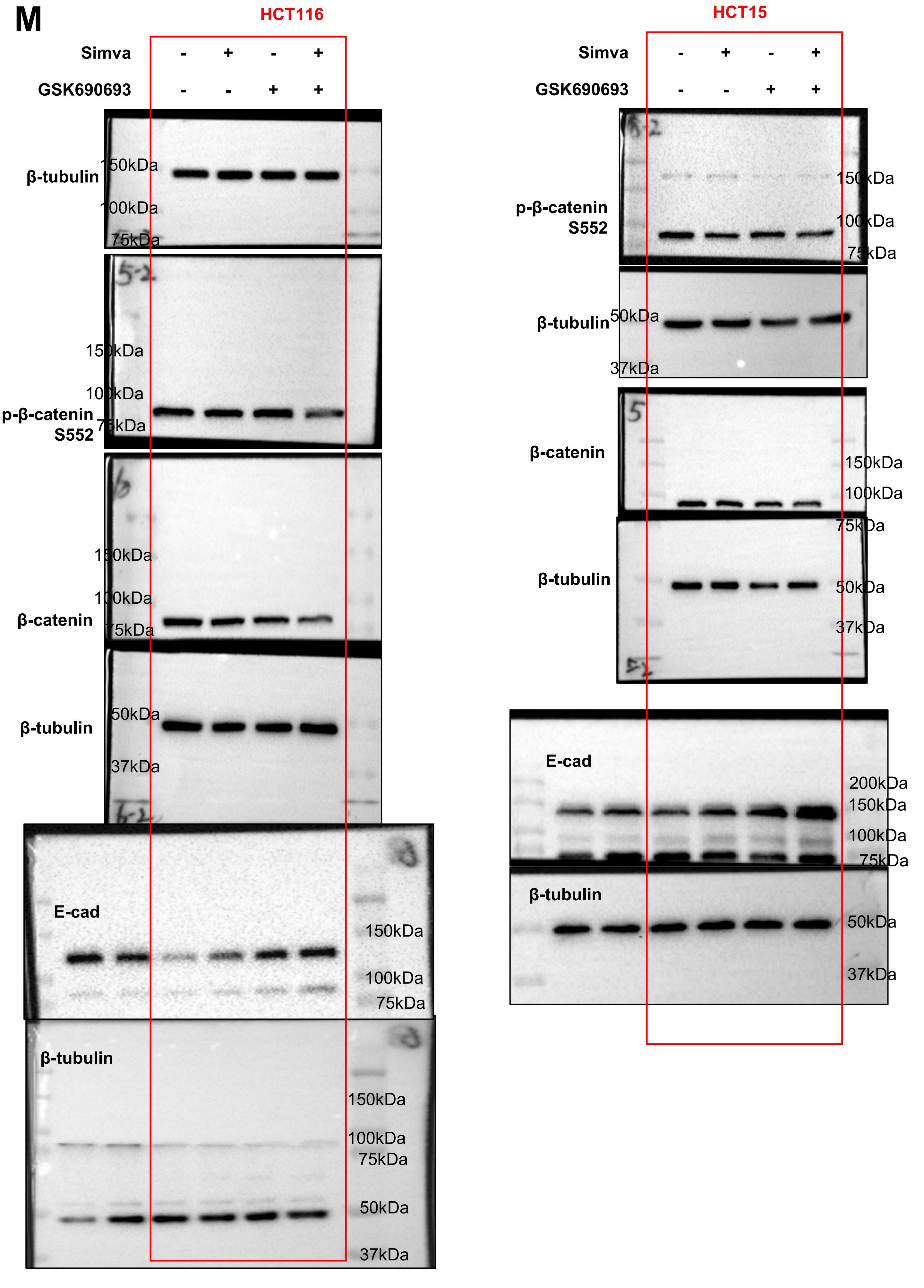
M
HCT116
Simva - + - +
GSK690693 - - + +
β-tubulin
150kDa
100kDa
75kDa
p-β-catenin S552
150kDa
100kDa
75kDa
β-catenin
150kDa
100kDa
75kDa
β-tubulin
50kDa
37kDa
E-cad
150kDa
100kDa
75kDa
β-tubulin
150kDa
100kDa
75kDa
50kDa
37kDa
HCT15
Simva - + - +
GSK690693 - - + +
p-β-catenin S552
150kDa
100kDa
75kDa
β-tubulin
50kDa
37kDa
β-catenin
150kDa
100kDa
75kDa
β-tubulin
50kDa
37kDa
E-cad
200kDa
150kDa
100kDa
75kDa
β-tubulin
50kDa
37kDa

Figure 5

C

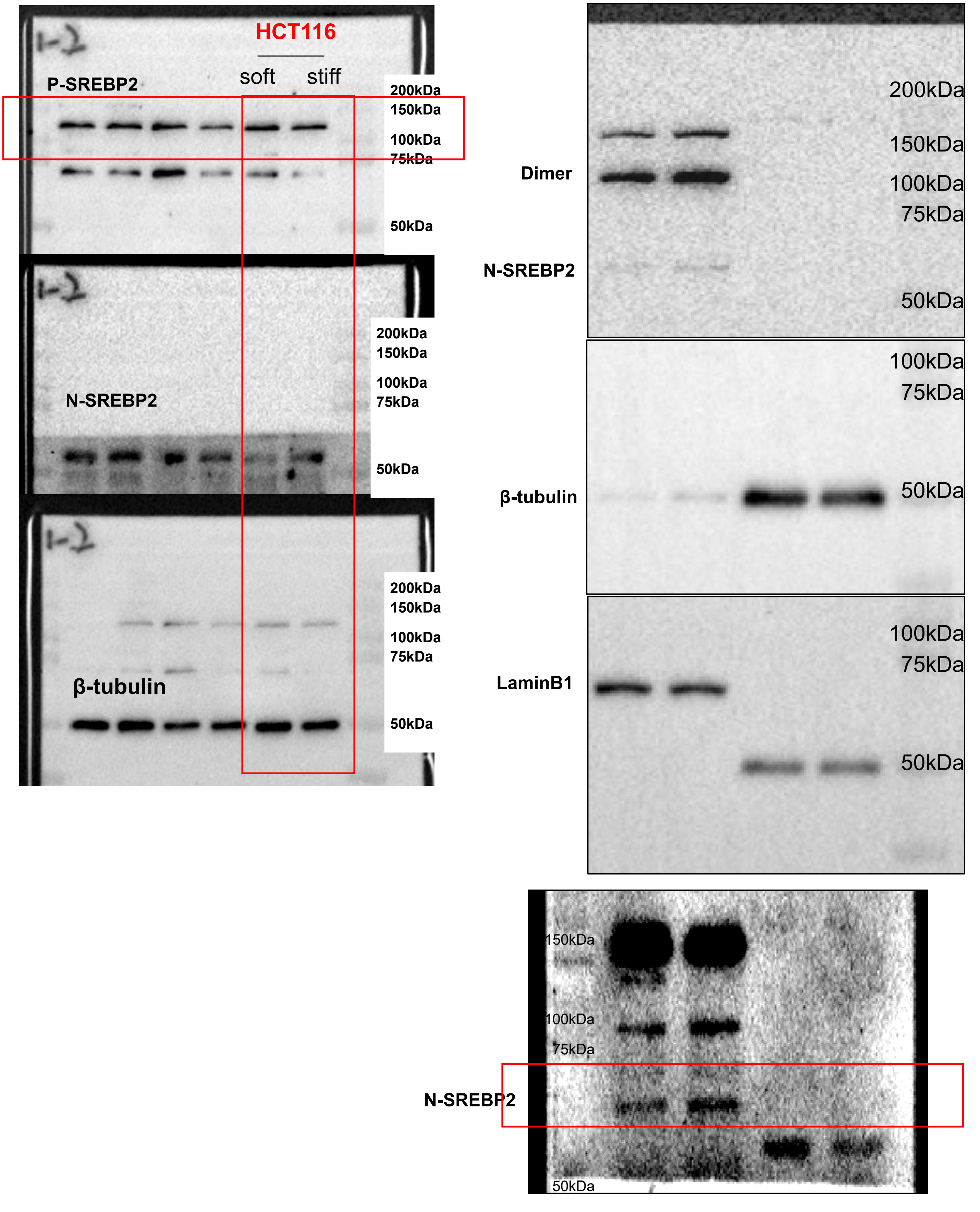
HCT116
soft
stiff
P-SREBP2
200kDa
150kDa
100kDa
75kDa
50kDa
N-SREBP2
β-tubulin
Nucle
Cyto
Soft
Stiff
Soft
Stiff
Dimer
N-SREBP2
β-tubulin
LaminB1
N-SREBP2

Figure 5

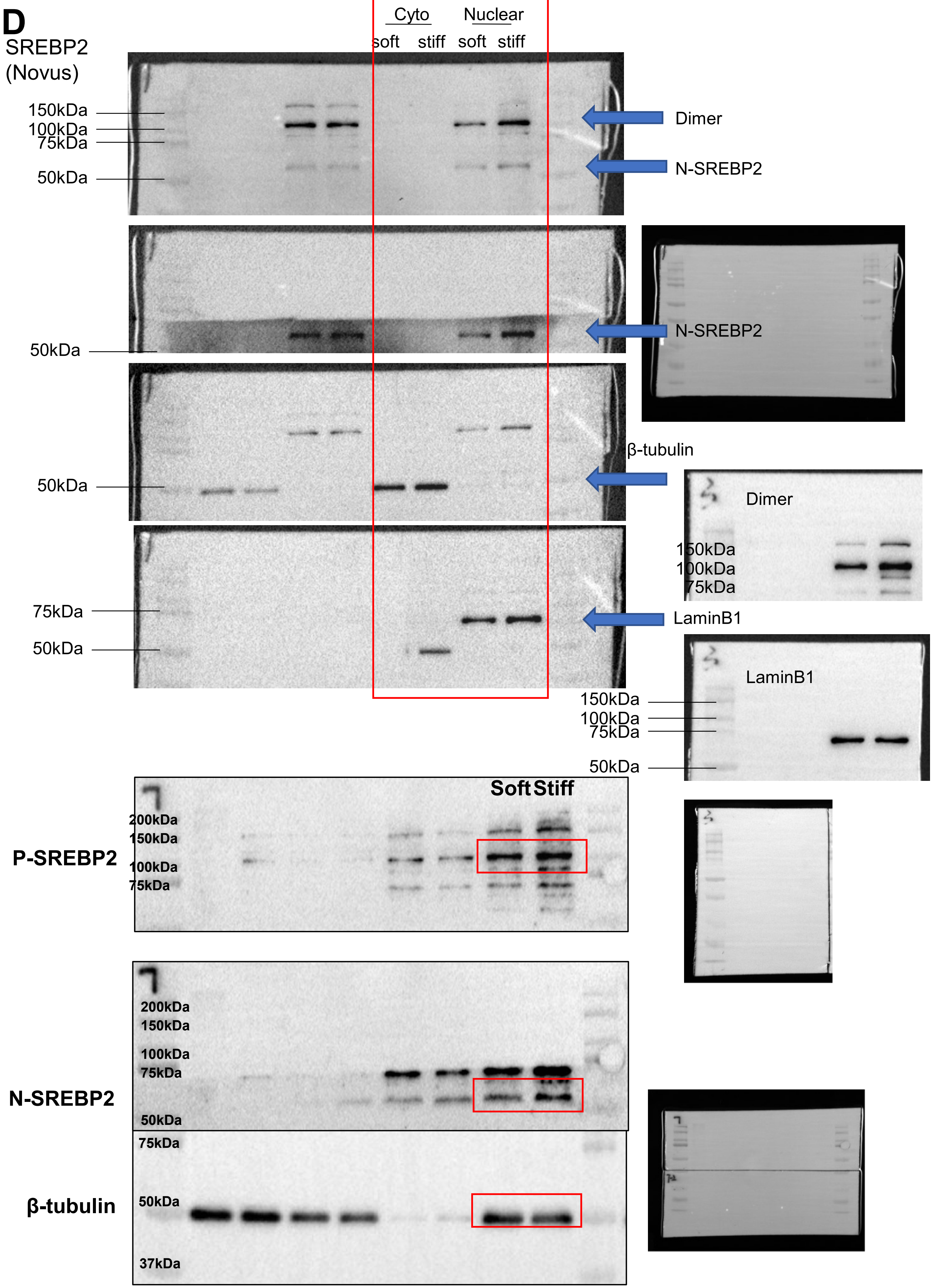
D
SREBP2
(Novus)
HCT15
Cyto
Nuclear
soft
stiff
soft
stiff
150kDa
100kDa
75kDa
50kDa
Dimer
N-SREBP2
50kDa
N-SREBP2
50kDa
β-tubulin
Dimer
150kDa
100kDa
75kDa
75kDa
50kDa
LaminB1
LaminB1
150kDa
100kDa
75kDa
50kDa
P-SREBP2
Soft
Stiff
200kDa
150kDa
100kDa
75kDa
N-SREBP2
200kDa
150kDa
100kDa
75kDa
50kDa
β-tubulin
75kDa
50kDa
37kDa

Figure 5

F

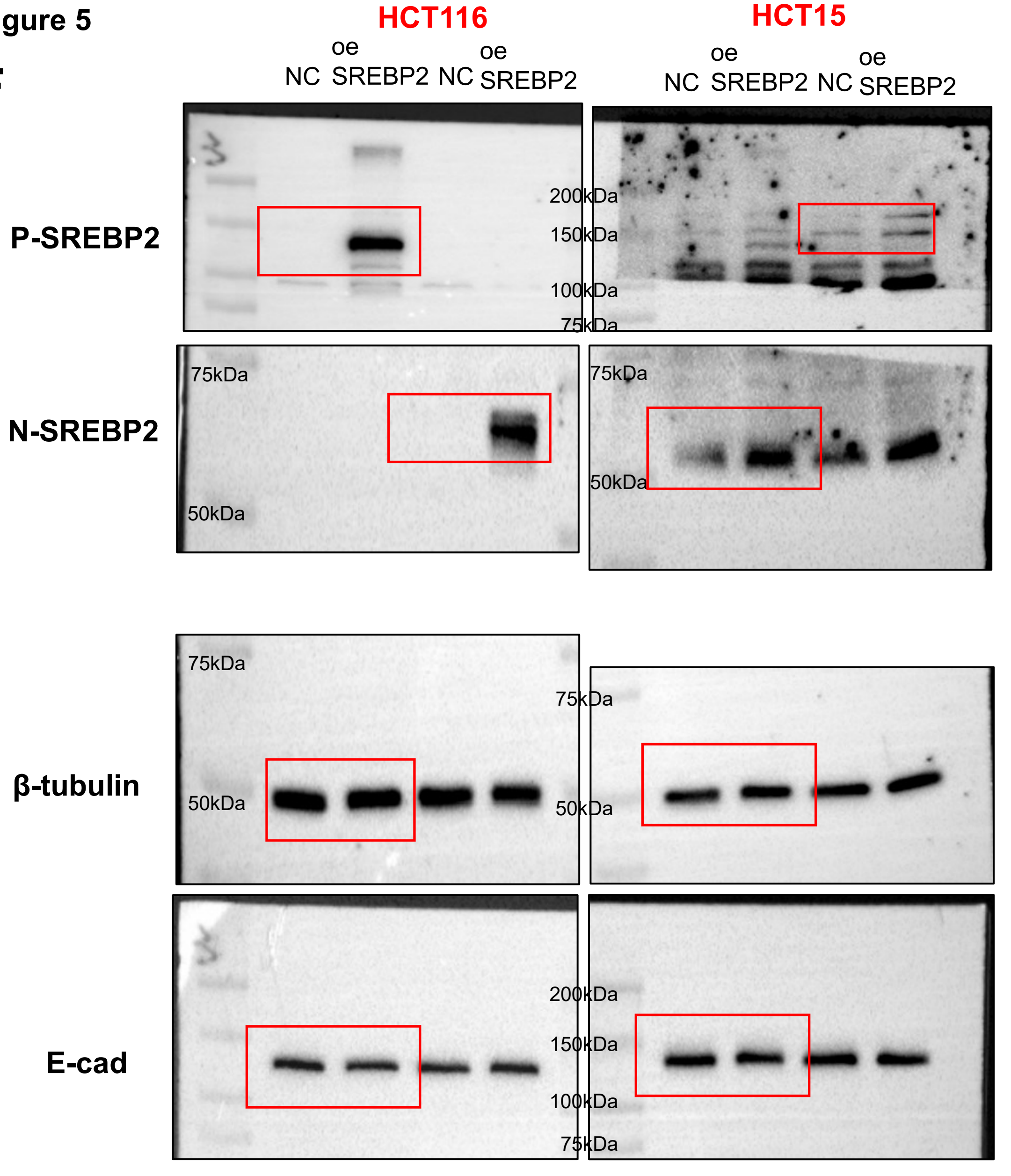

HCT116
HCT15
NC
oe SREBP2
NC
oe SREBP2
NC
oe SREBP2
NC
oe SREBP2
P-SREBP2
N-SREBP2
β-tubulin
E-cad
200kDa
150kDa
100kDa
75kDa
50kDa

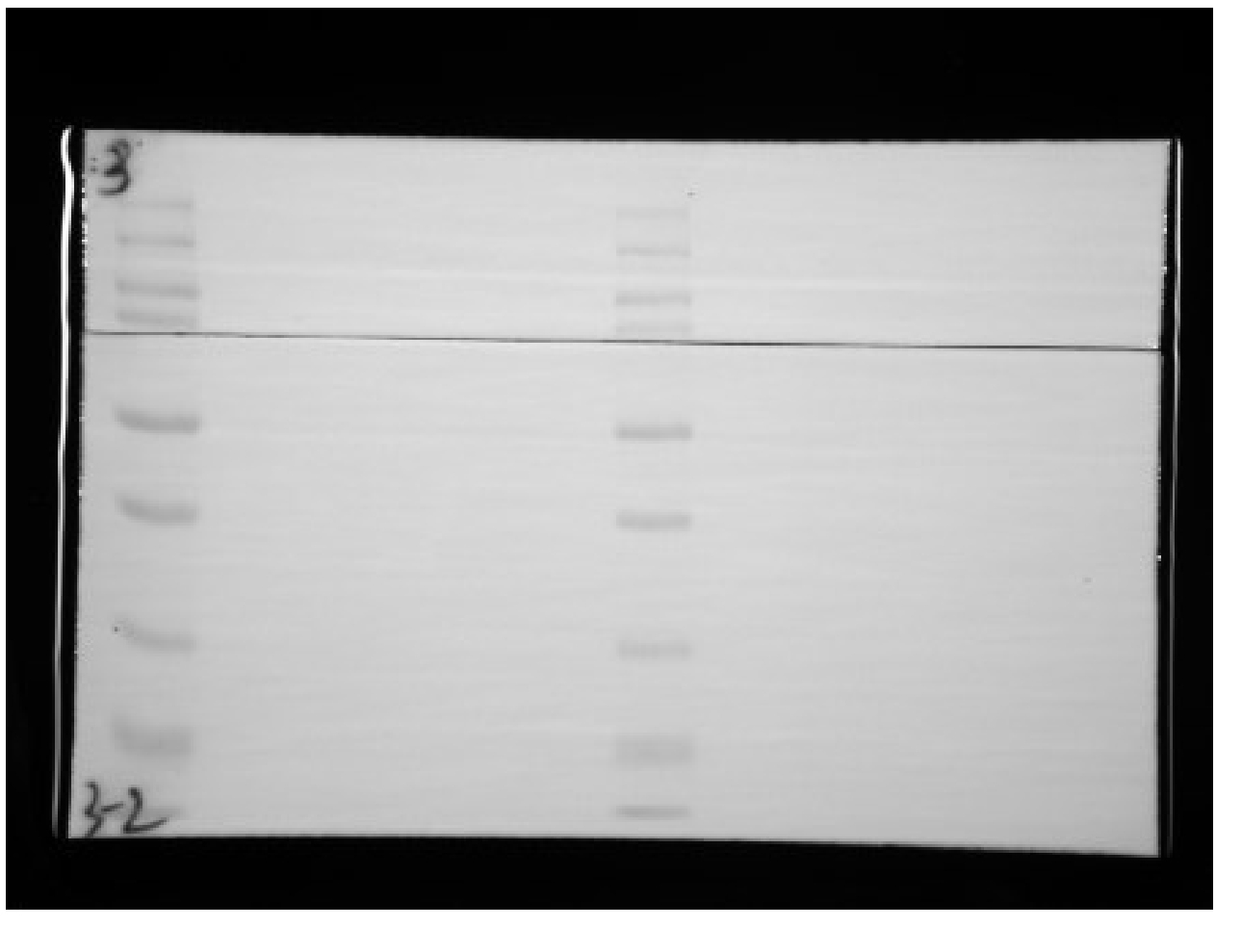

3
3-2

Figure 5

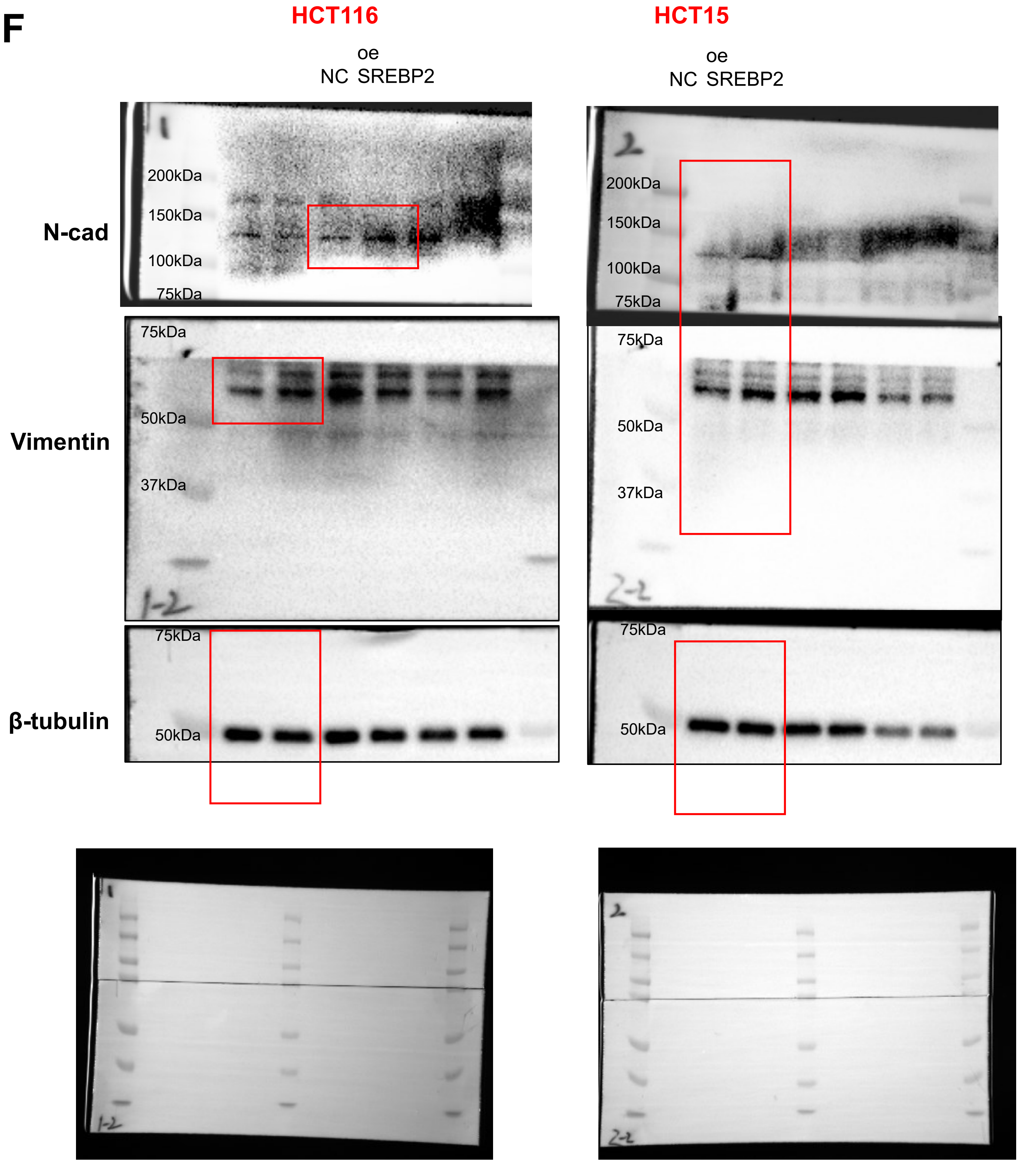
F
HCT116
HCT15
oe
NC SREBP2
oe
NC SREBP2
N-cad
Vimentin
β-tubulin
200kDa
150kDa
100kDa
75kDa
75kDa
50kDa
37kDa
75kDa
50kDa
200kDa
150kDa
100kDa
75kDa
75kDa
50kDa
37kDa
75kDa
50kDa

Figure 5

F

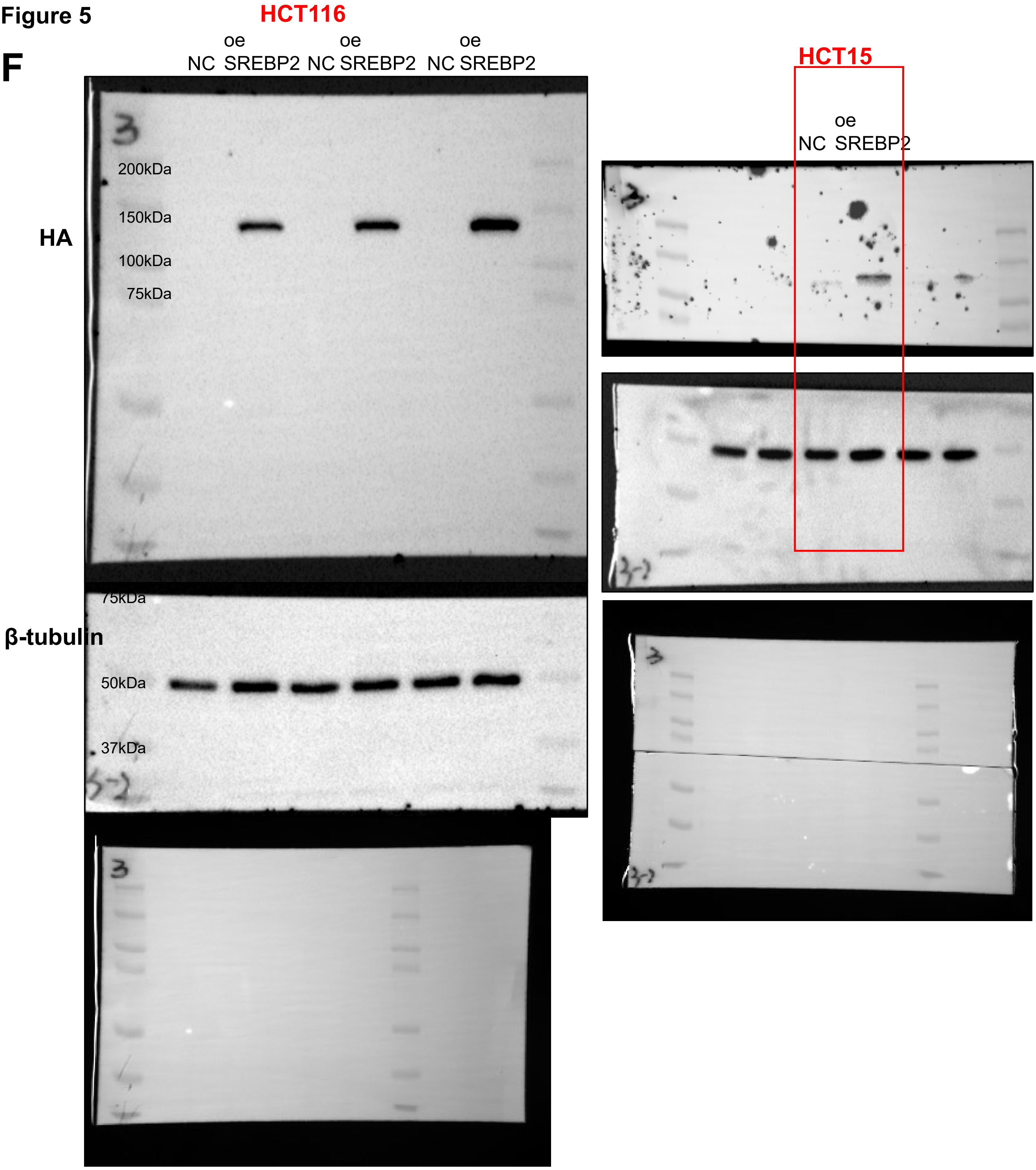

HCT116
NC
oe SREBP2
NC
oe SREBP2
NC
oe SREBP2
HA
200kDa
150kDa
100kDa
75kDa
β-tubulin
75kDa
50kDa
37kDa
HCT15
NC
oe SREBP2

Figure 5

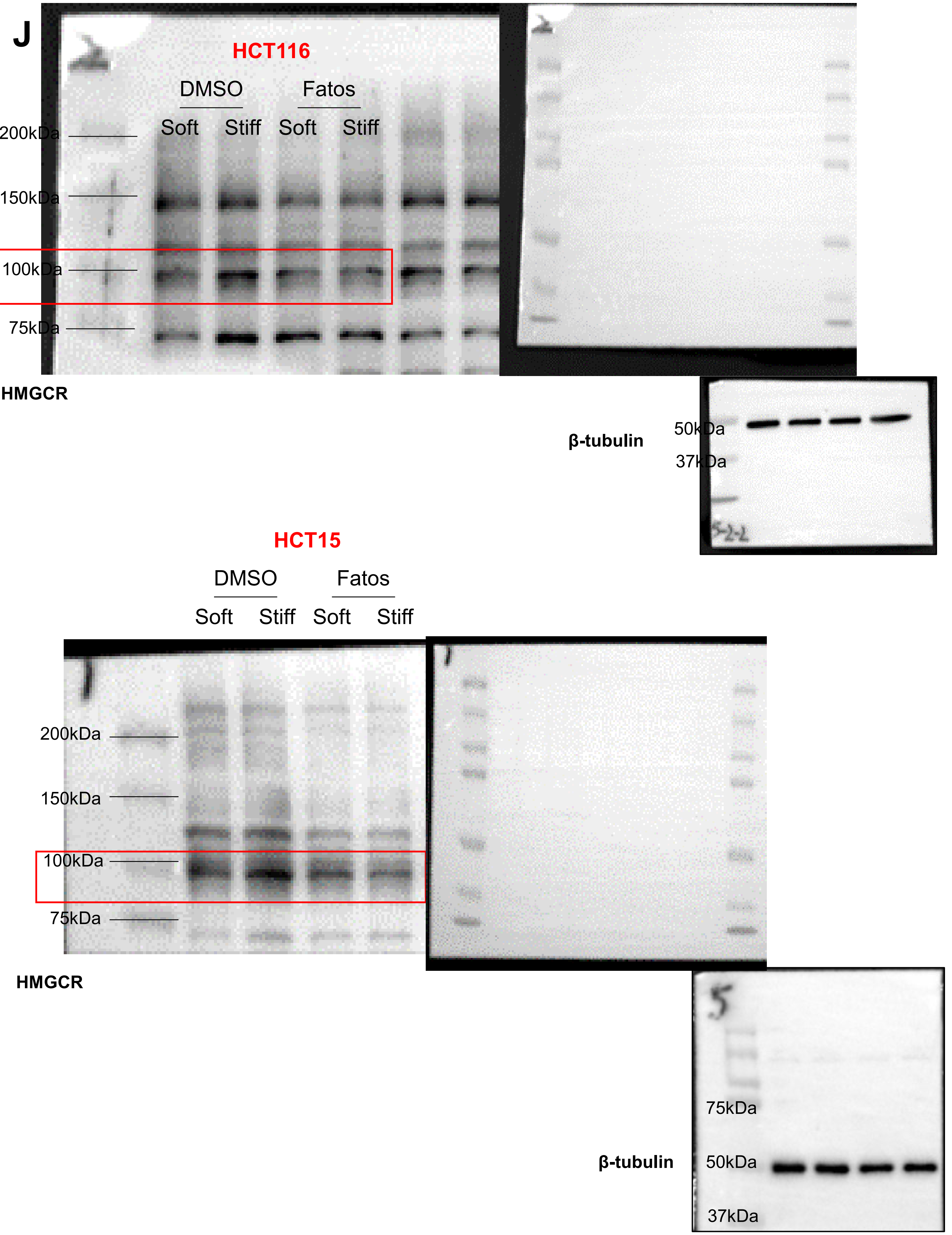
J
HCT116
DMSO
Fatos
Soft
Stiff
Soft
Stiff
200kDa
150kDa
100kDa
75kDa
HMGCR
β-tubulin
50kDa
37kDa
HCT15
DMSO
Fatos
Soft
Stiff
Soft
Stiff
200kDa
150kDa
100kDa
75kDa
HMGCR
75kDa
β-tubulin
50kDa
37kDa

Figure 6

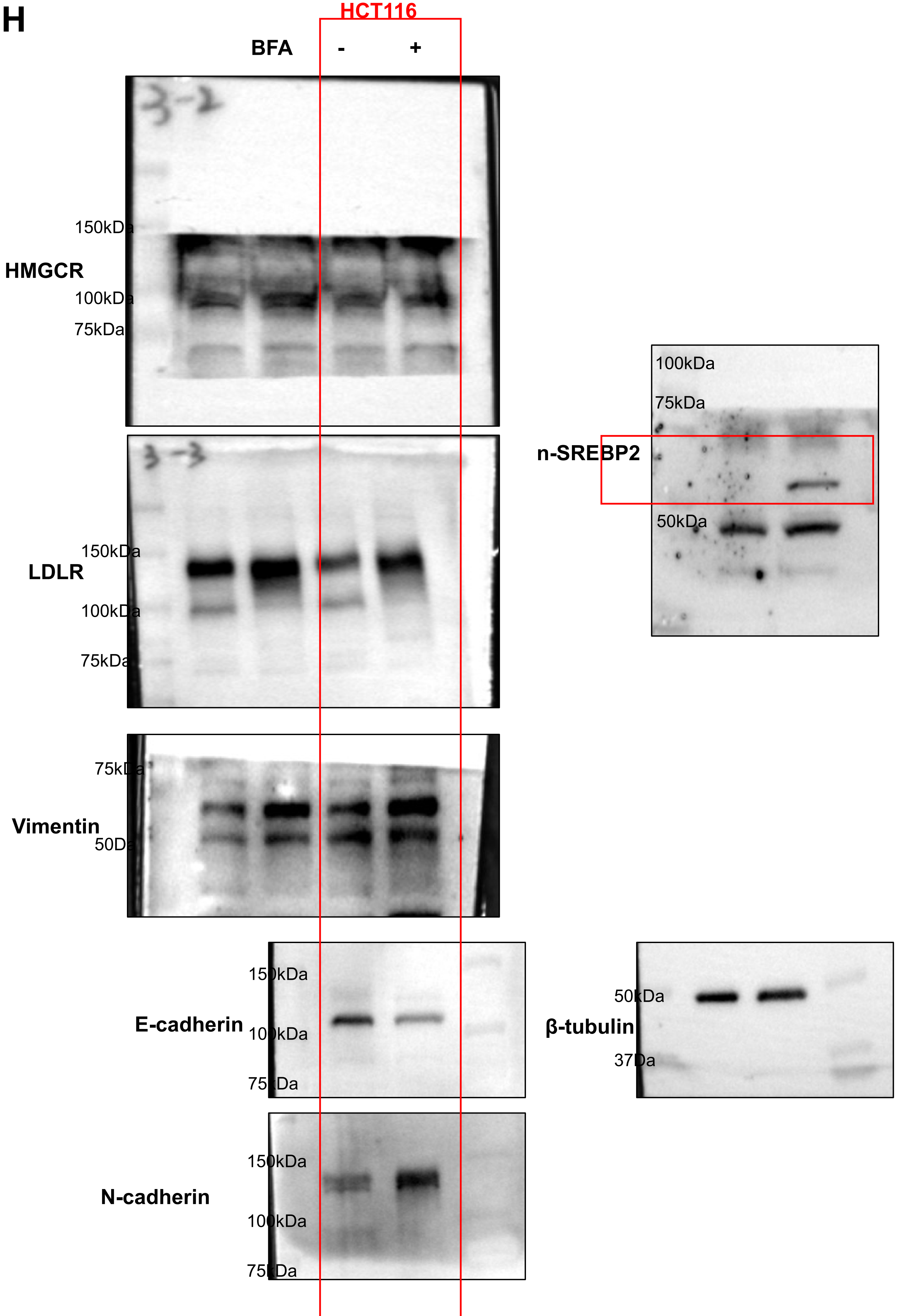
H
HCT116
BFA
-
+
3-2
150kDa
HMGCR
100kDa
75kDa
3-3
150kDa
LDLR
100kDa
75kDa
75kDa
Vimentin
50Da
150kDa
E-cadherin
100kDa
75kDa
150kDa
N-cadherin
100kDa
75kDa
100kDa
75kDa
n-SREBP2
50kDa
50kDa
β-tubulin
37Da

Figure 6

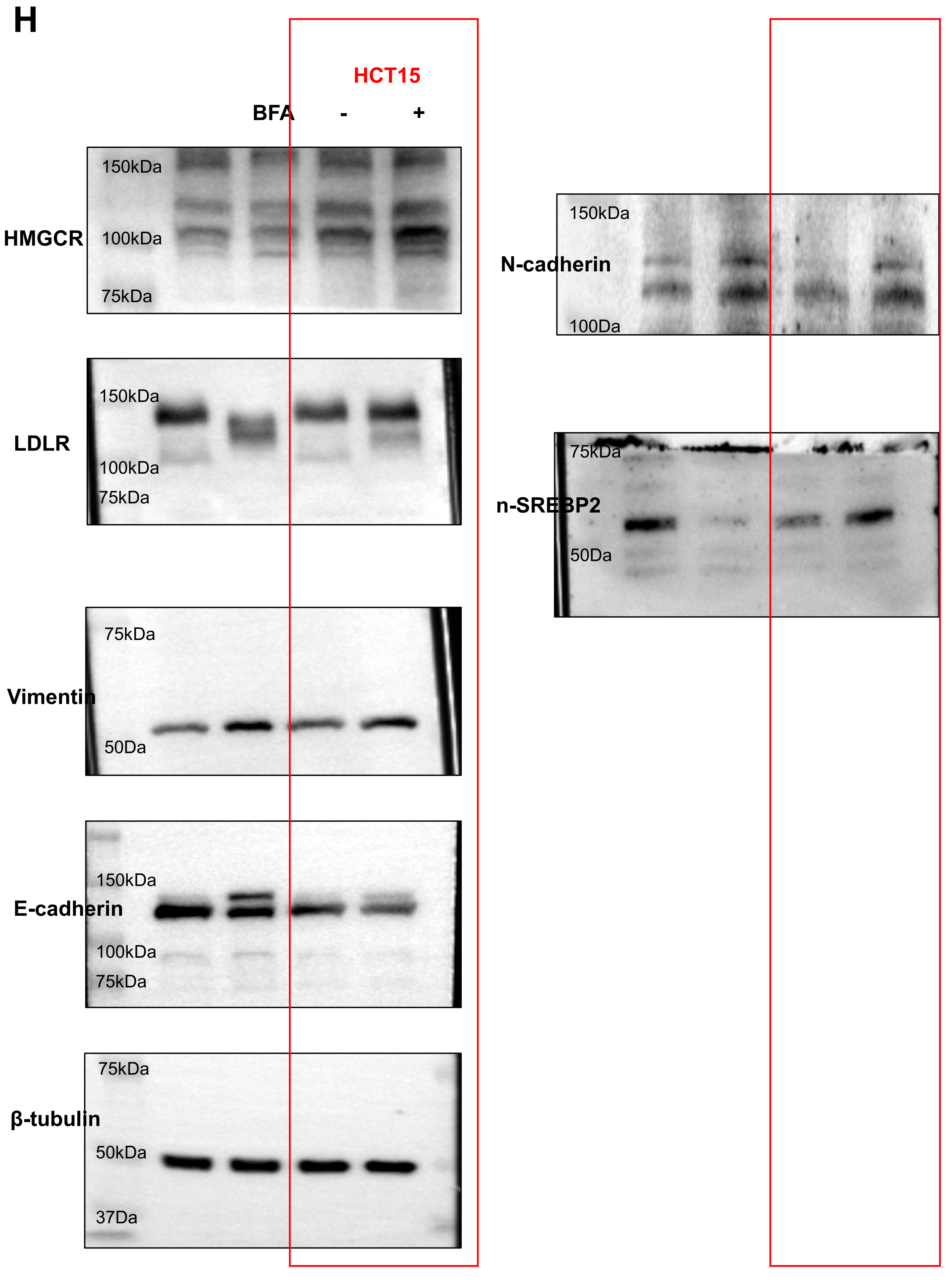
H
HCT15
BFA
-
+
HMGCR
150kDa
100kDa
75kDa
LDLR
150kDa
100kDa
75kDa
Vimentin
75kDa
50Da
E-cadherin
150kDa
100kDa
75kDa
β-tubulin
75kDa
50kDa
37Da
N-cadherin
150kDa
100Da
n-SREBP2
75kDa
50Da

Figure 6

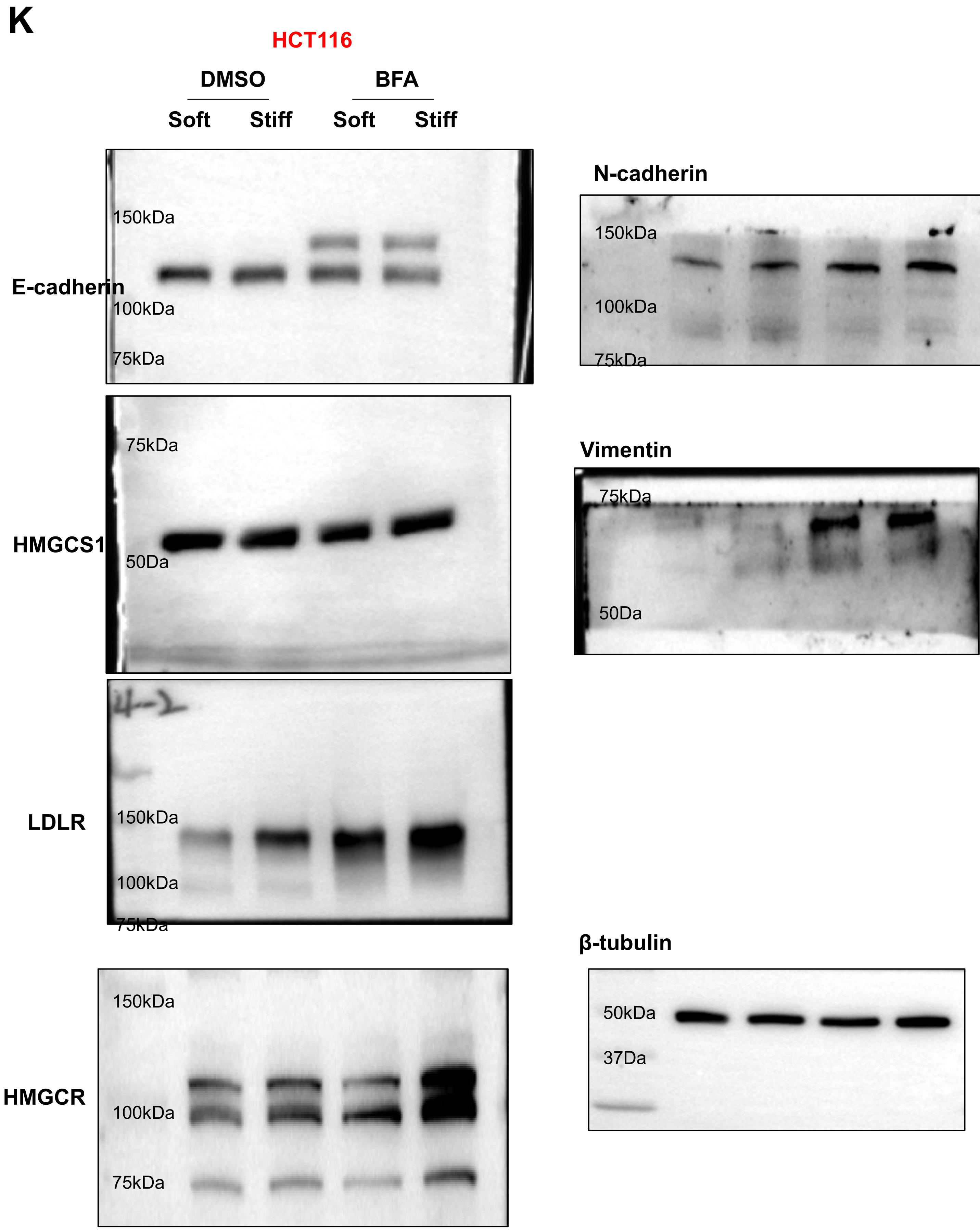
K
HCT116
DMSO
BFA
Soft
Stiff
Soft
Stiff
E-cadherin
150kDa
100kDa
75kDa
HMGCS1
75kDa
50Da
LDLR
150kDa
100kDa
75kDa
HMGCR
150kDa
100kDa
75kDa
N-cadherin
150kDa
100kDa
75kDa
Vimentin
75kDa
50Da
β-tubulin
50kDa
37Da

Figure 6

K

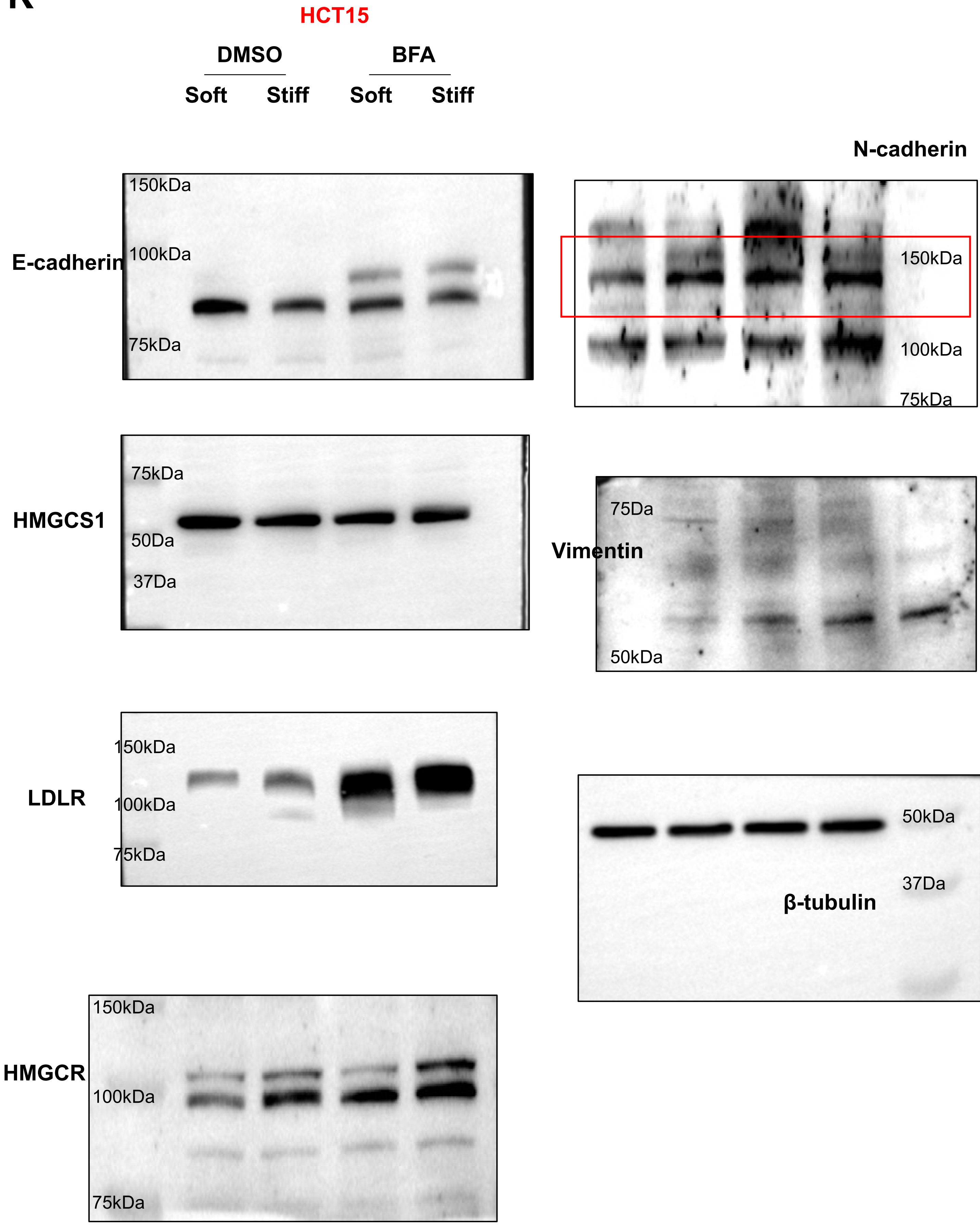
HCT15
DMSO
BFA
Soft
Stiff
Soft
Stiff
E-cadherin
150kDa
100kDa
75kDa
N-cadherin
150kDa
100kDa
75kDa
HMGCS1
75kDa
50Da
37Da
Vimentin
75Da
50kDa
LDLR
150kDa
100kDa
75kDa
β-tubulin
50kDa
37Da
HMGCR
150kDa
100kDa
75kDa

Figure 6

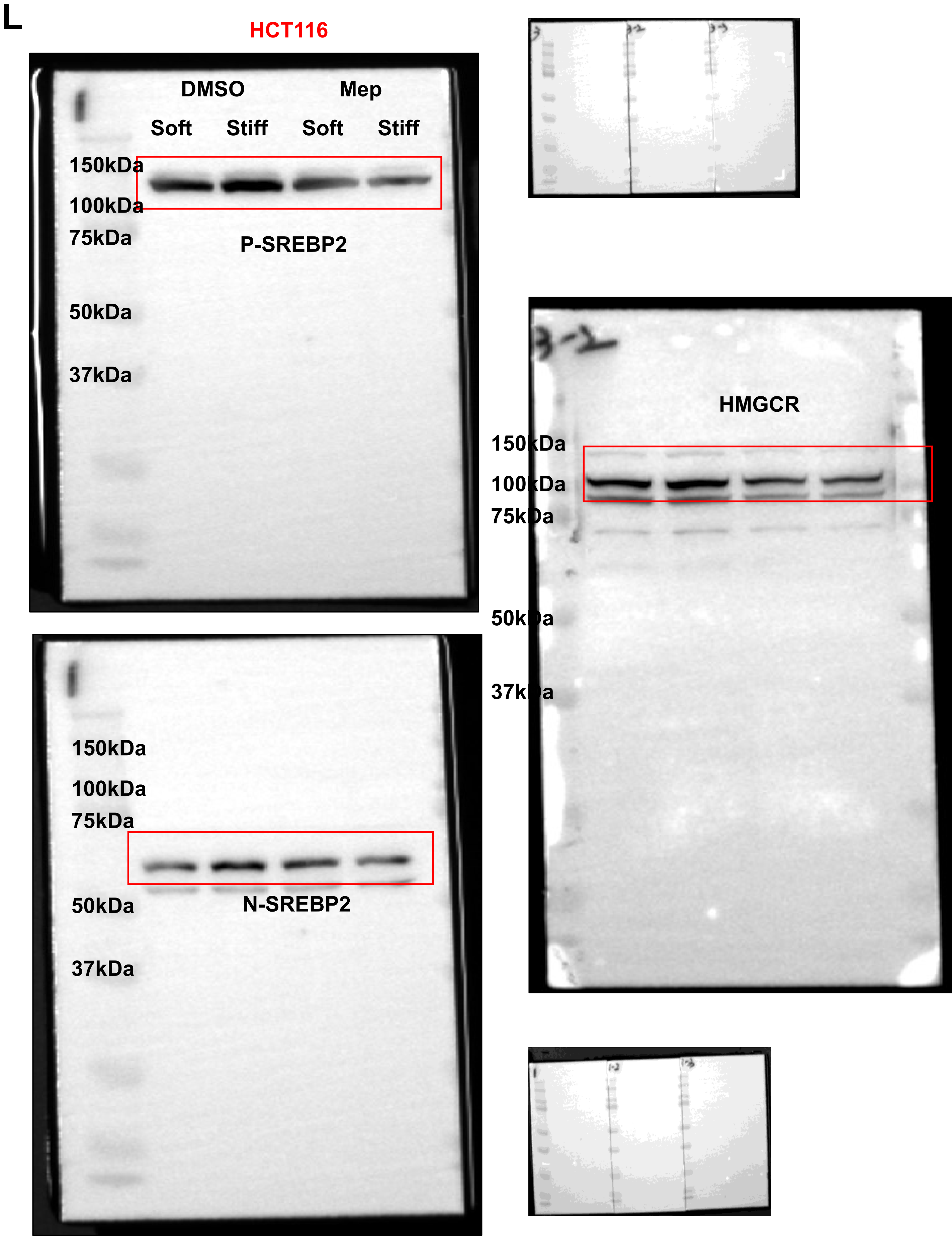
L
HCT116
DMSO
Mep
Soft
Stiff
Soft
Stiff
150kDa
100kDa
75kDa
P-SREBP2
50kDa
37kDa
150kDa
100kDa
75kDa
50kDa
N-SREBP2
37kDa
HMGCR
150kDa
100kDa
75kDa
50kDa
37kDa

Figure 6

L

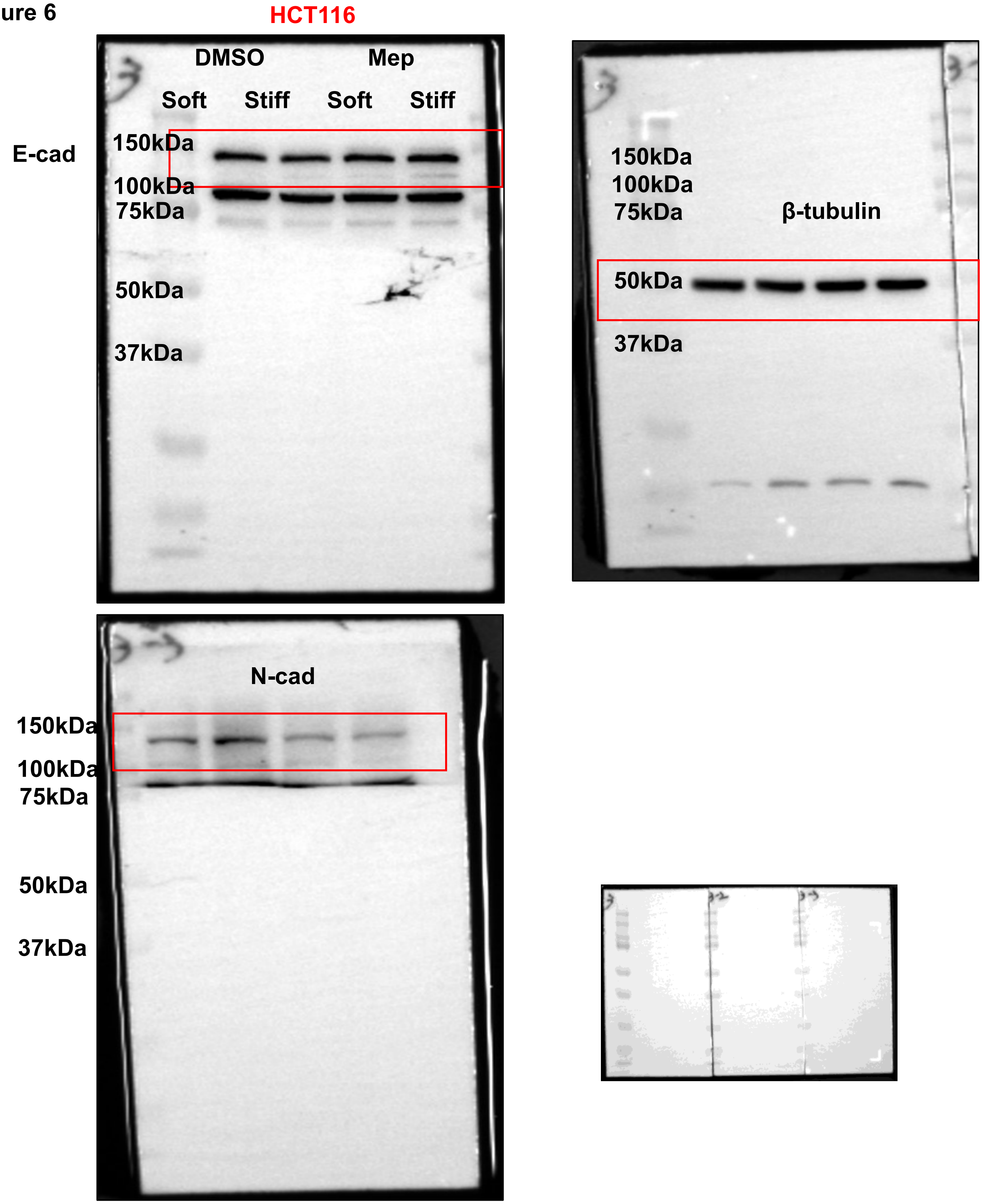
HCT116
DMSO
Mep
Soft
Stiff
Soft
Stiff
E-cad
150kDa
100kDa
75kDa
50kDa
37kDa
150kDa
100kDa
75kDa
β-tubulin
50kDa
37kDa
N-cad
150kDa
100kDa
75kDa
50kDa
37kDa

Figure 6

L

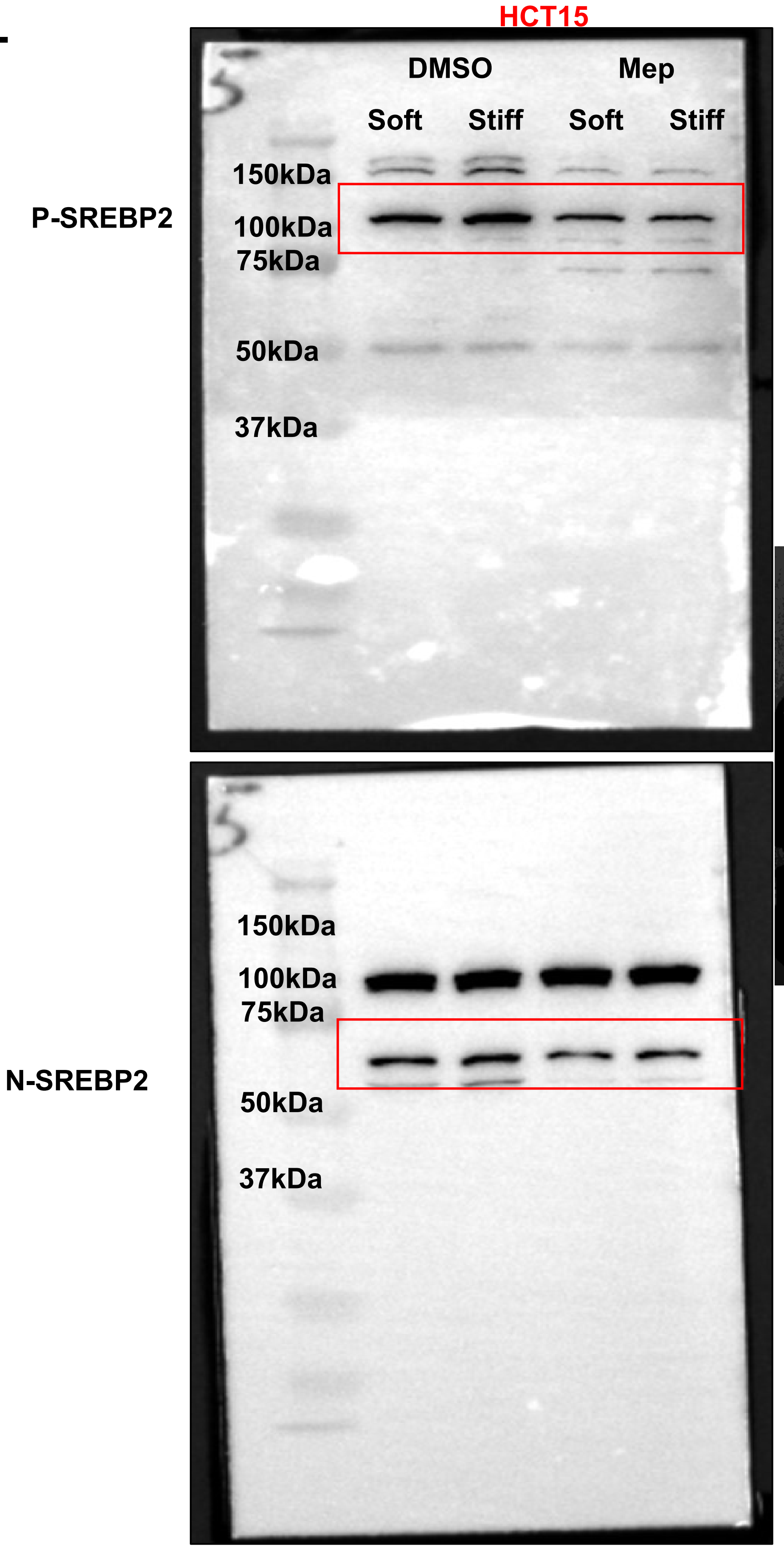

HCT15
DMSO
Mep
Soft
Stiff
Soft
Stiff
P-SREBP2
150kDa
100kDa
75kDa
50kDa
37kDa
N-SREBP2
150kDa
100kDa
75kDa
50kDa
37kDa

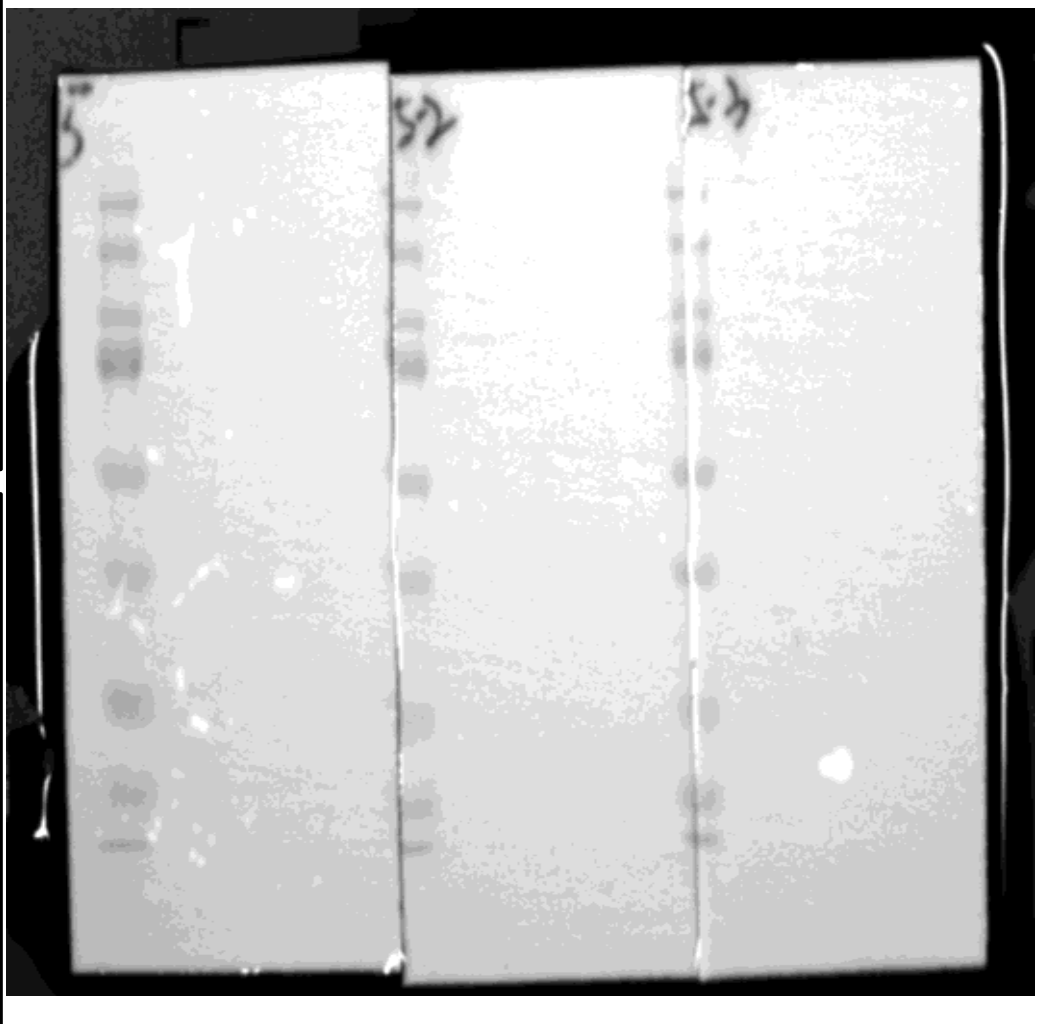

Figure 6

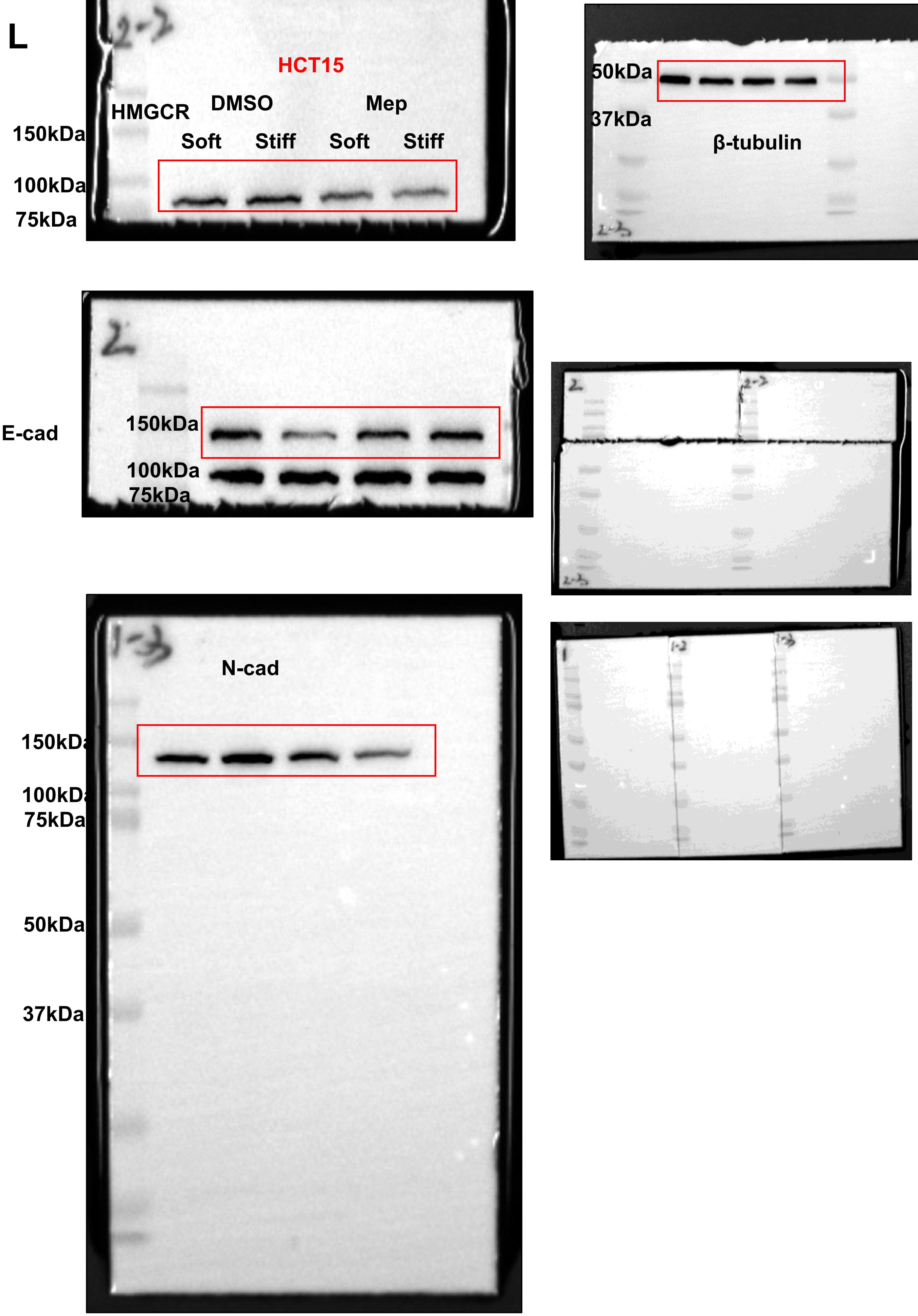
L
HCT15
HMGCR
DMSO
Mep
Soft
Stiff
Soft
Stiff
150kDa
100kDa
75kDa
50kDa
37kDa
β-tubulin
E-cad
150kDa
100kDa
75kDa
N-cad
150kDa
100kDa
75kDa
50kDa
37kDa

Figure 7

B-C

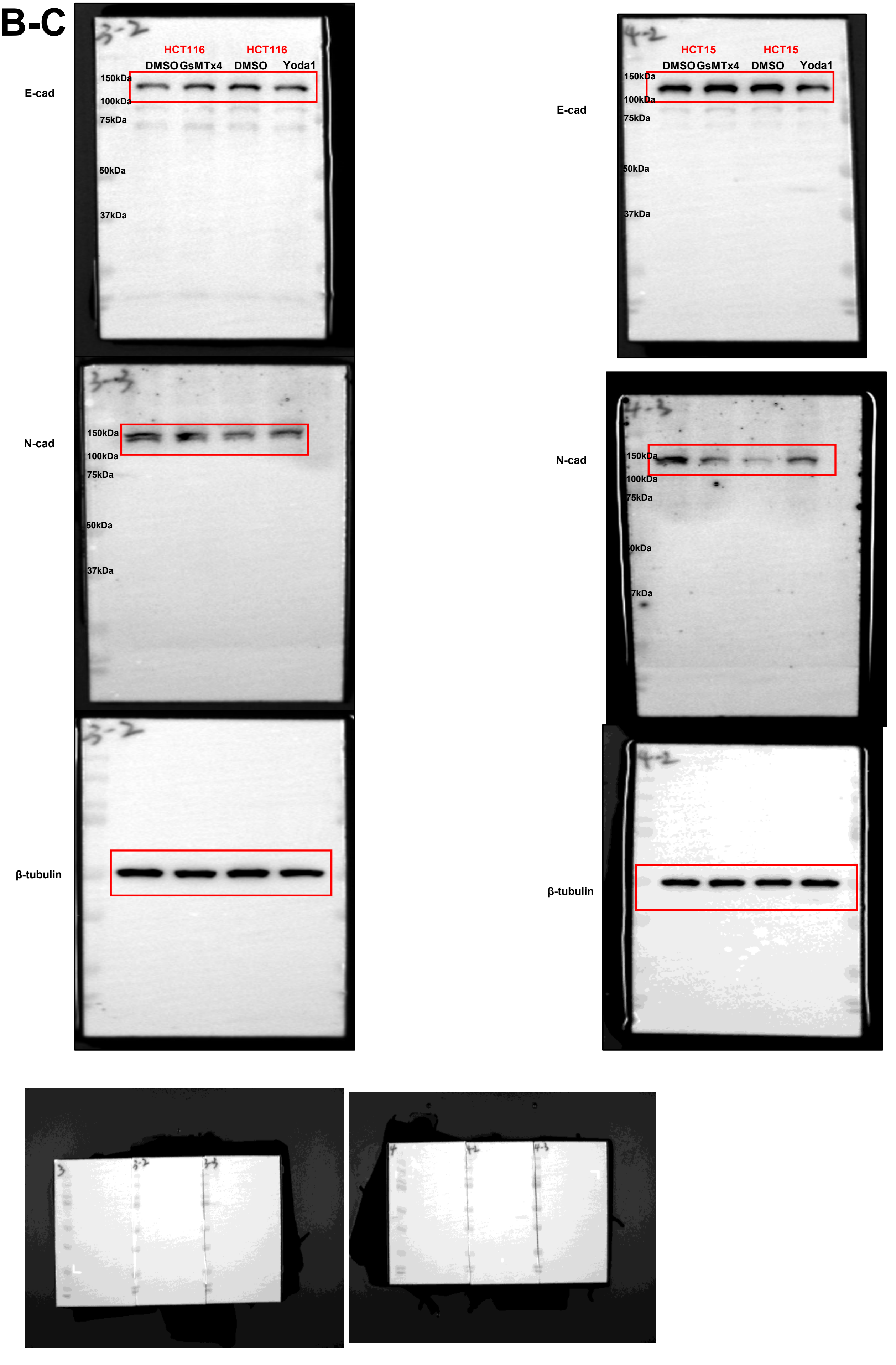
E-cad
N-cad
β-tubulin
HCT116
DMSO
GsMTx4
HCT116
DMSO
Yoda1
HCT15
DMSO
GsMTx4
HCT15
DMSO
Yoda1
150kDa
100kDa
75kDa
50kDa
37kDa

Figure 7

D/F

P-SREBP2

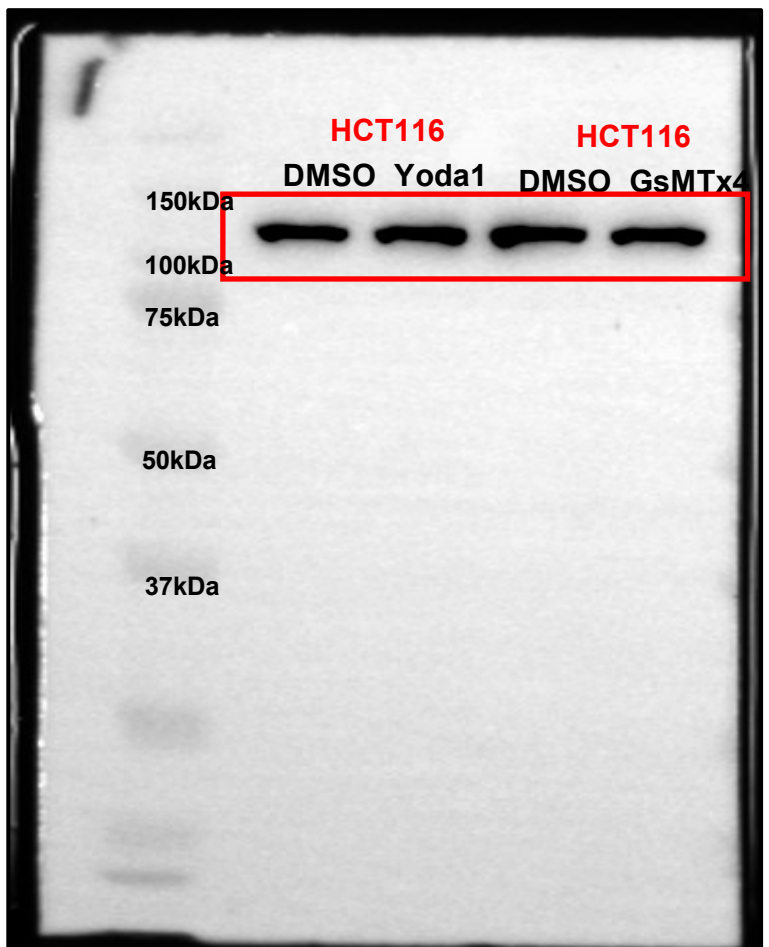

β-tubulin

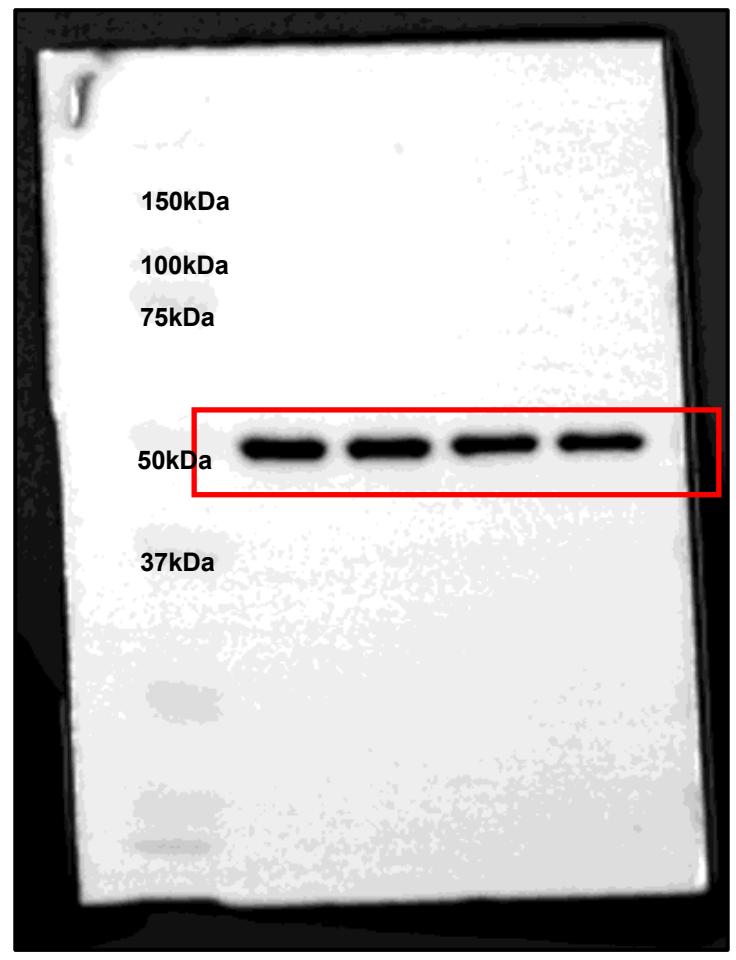

N-SREBP2

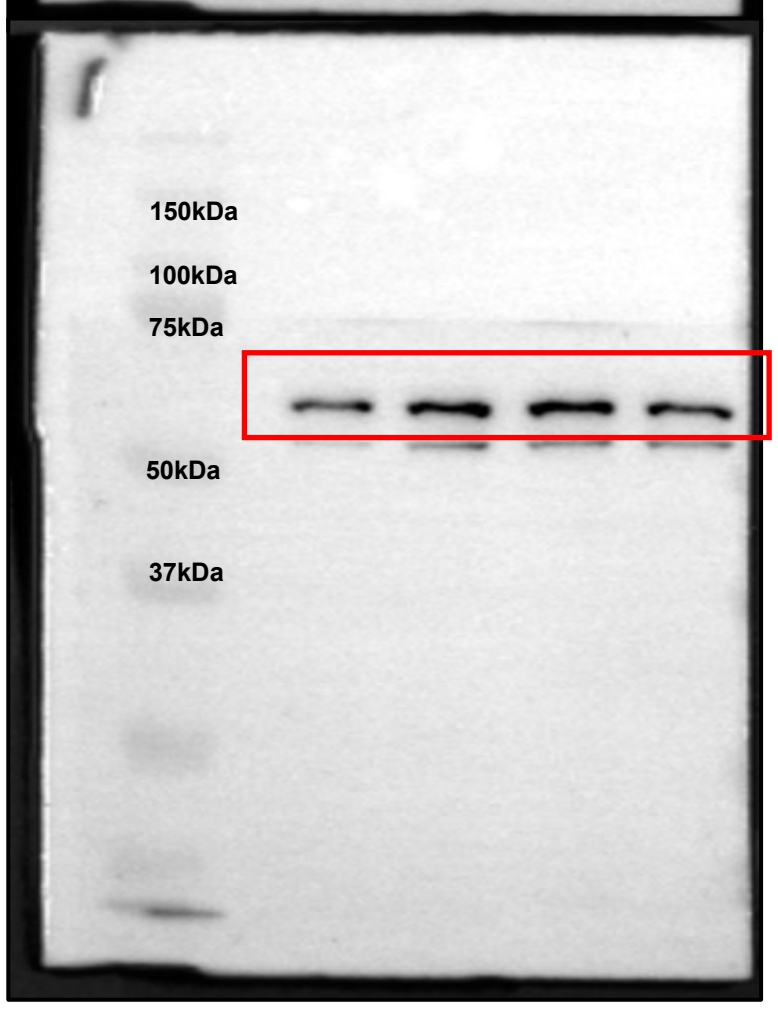

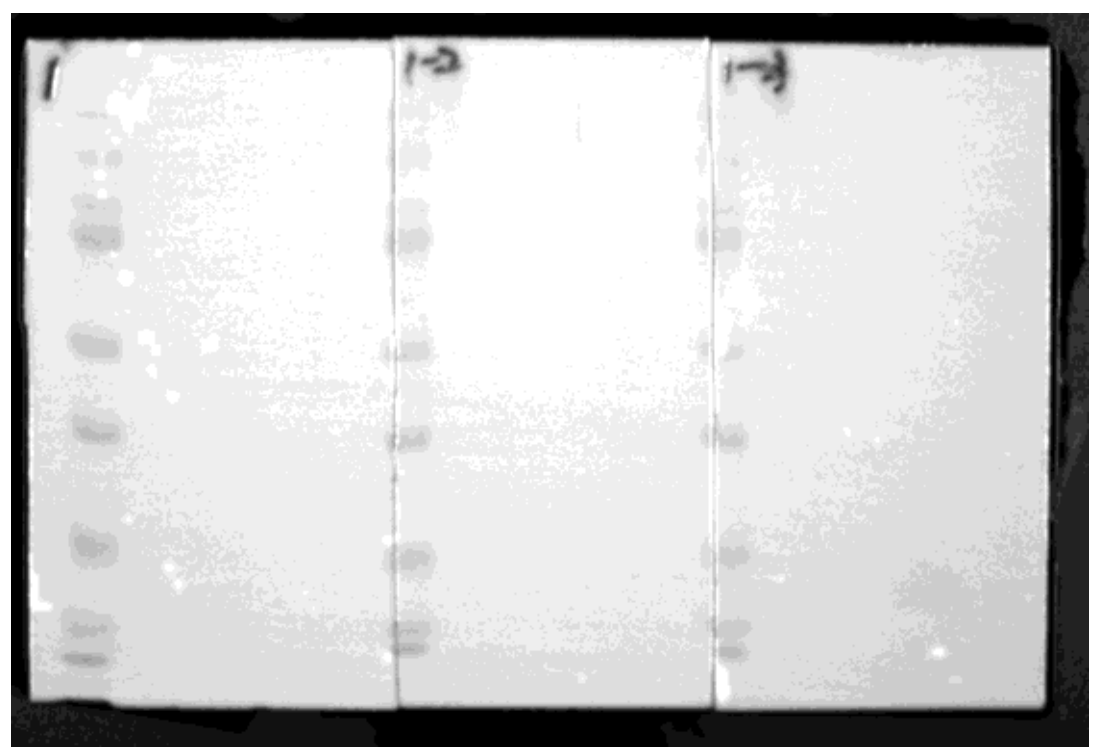

HMGCR

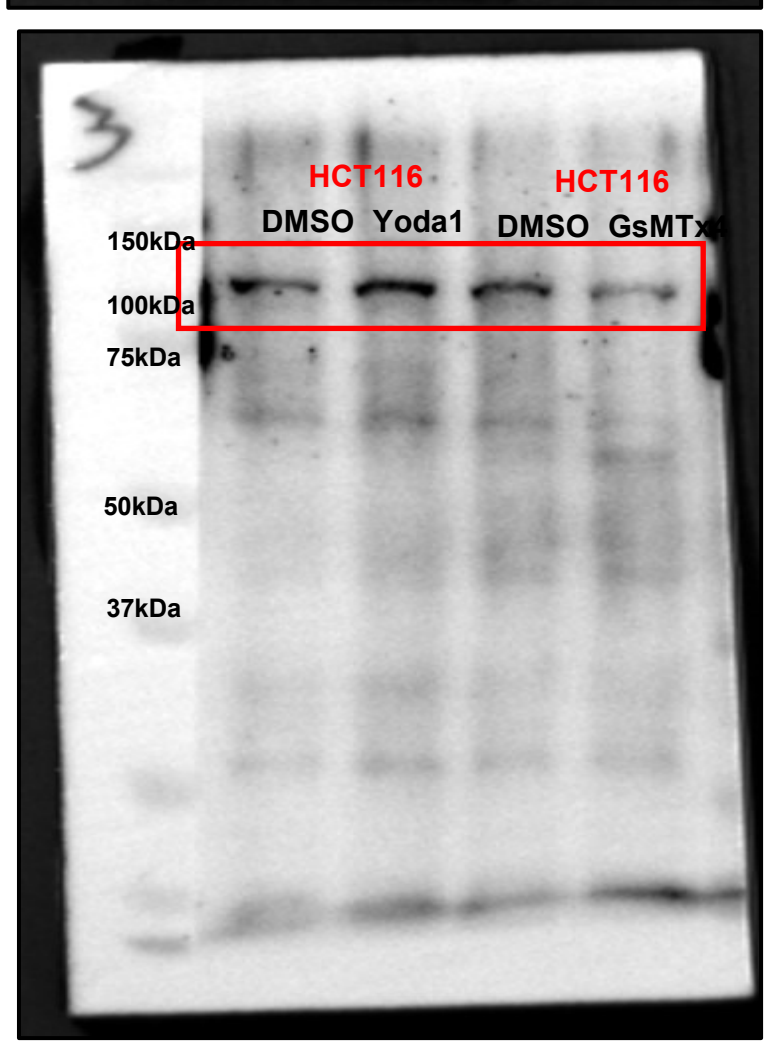

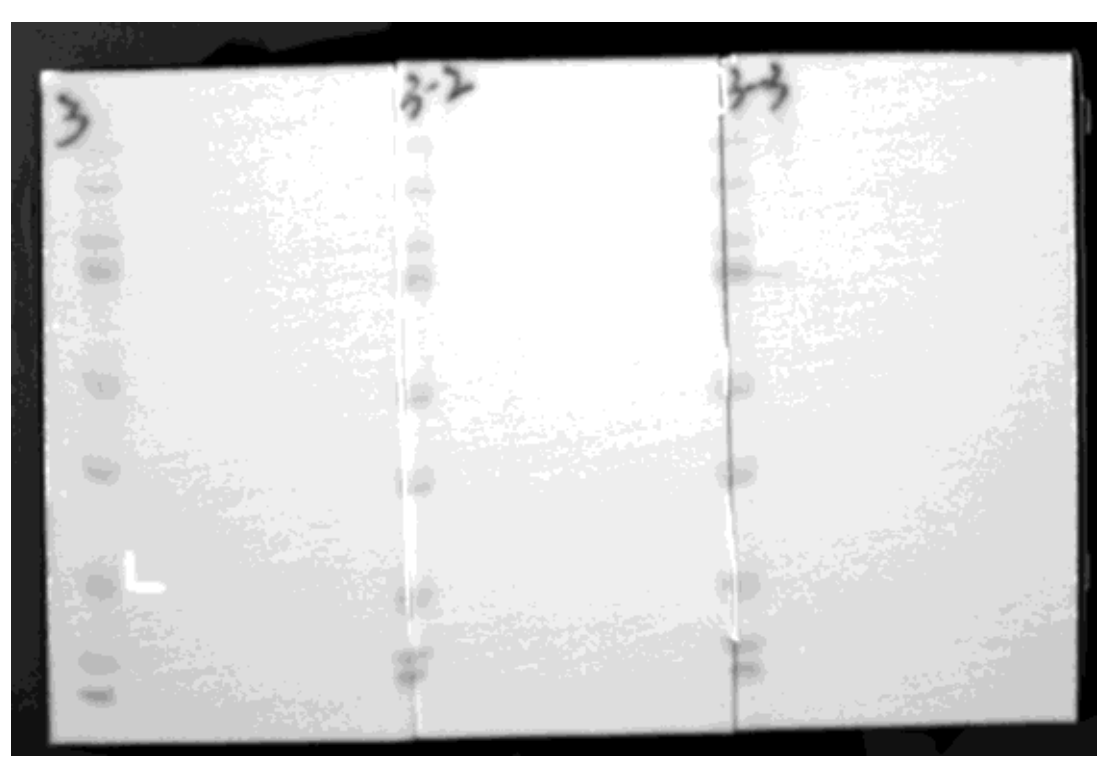

Figure 7

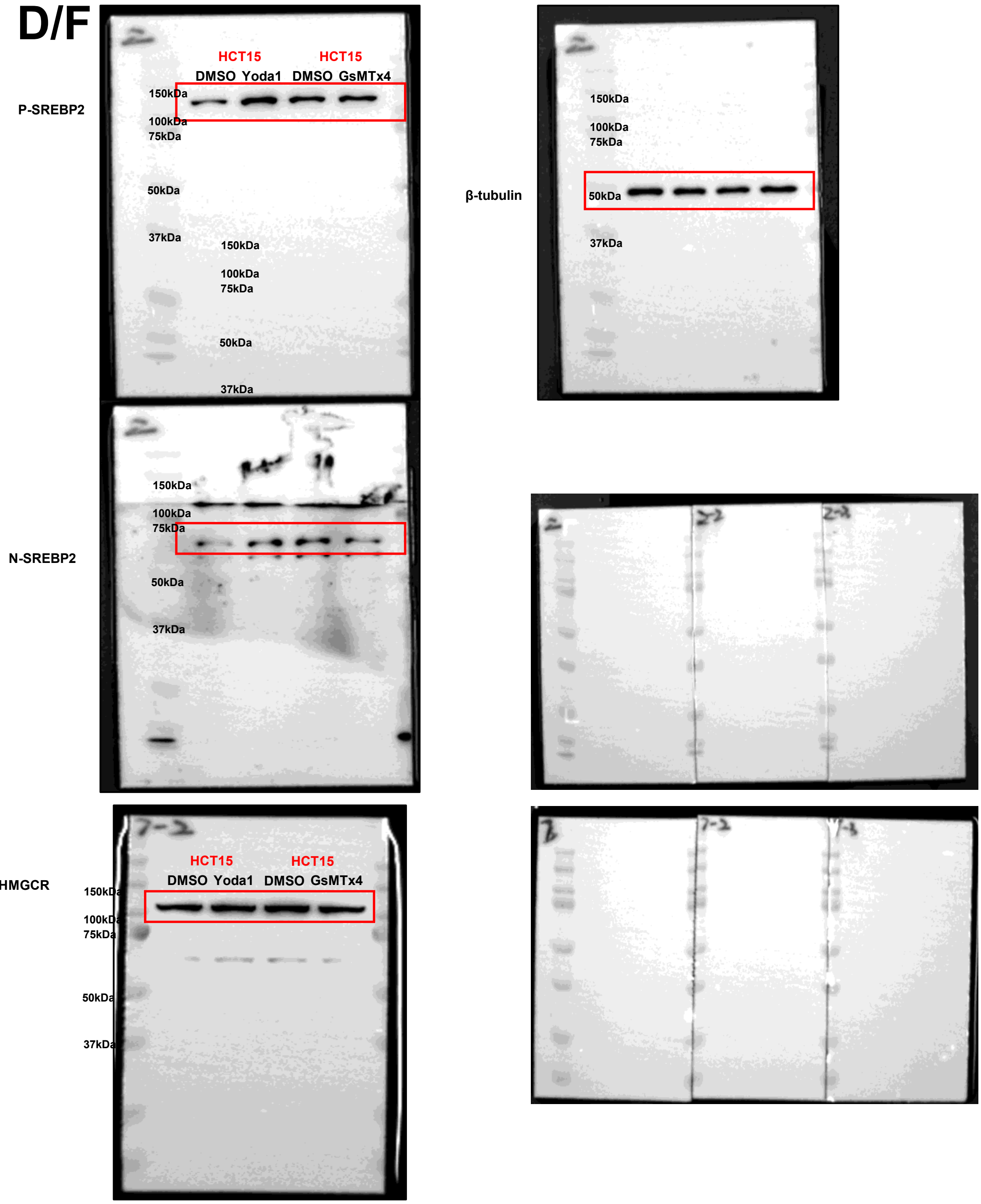
D/F
P-SREBP2
HCT15
HCT15
DMSO Yoda1 DMSO GsMTx4
150kDa
100kDa
75kDa
50kDa
37kDa
150kDa
100kDa
75kDa
50kDa
37kDa
β-tubulin
150kDa
100kDa
75kDa
50kDa
37kDa
N-SREBP2
150kDa
100kDa
75kDa
50kDa
37kDa
HMGCR
HCT15
HCT15
DMSO Yoda1 DMSO GsMTx4
150kDa
100kDa
75kDa
50kDa
37kDa

Figure 7

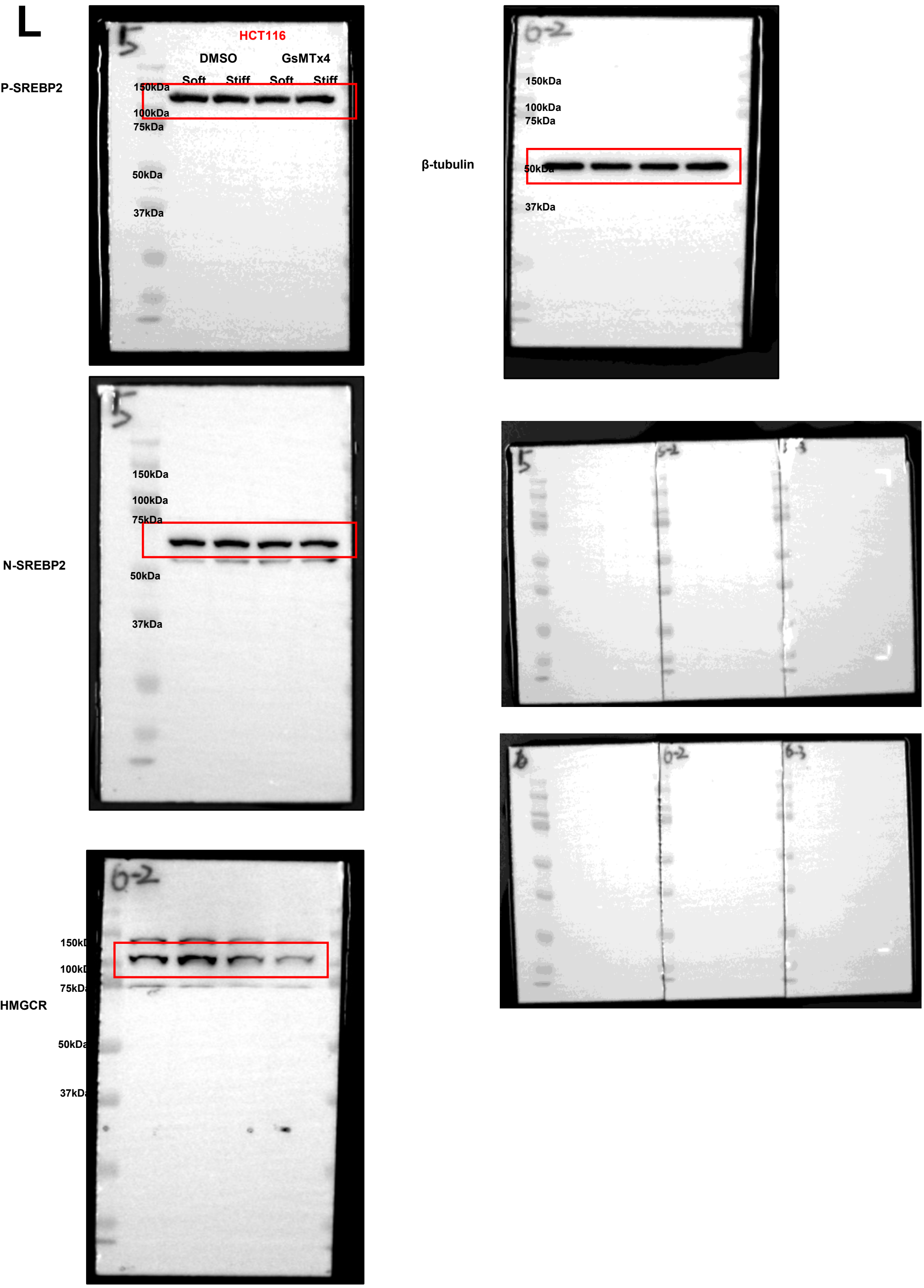
L
HCT116
DMSO
GsMTx4
Soft
Stiff
Soft
Stiff
P-SREBP2
150kDa
100kDa
75kDa
50kDa
37kDa
β-tubulin
150kDa
100kDa
75kDa
50kDa
37kDa
N-SREBP2
150kDa
100kDa
75kDa
50kDa
37kDa
HMGCR

Figure 7

L

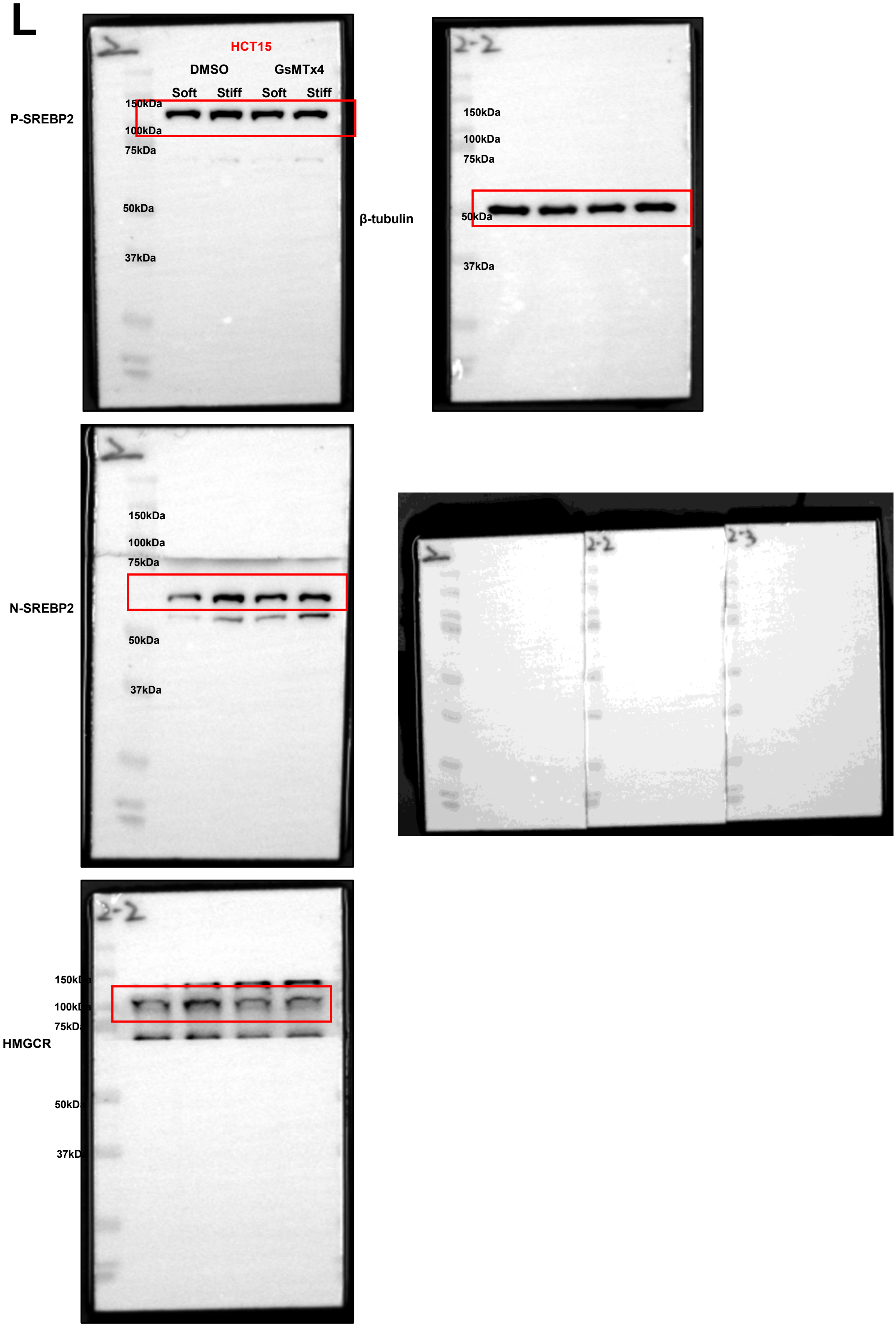
HCT15
DMSO
GsMTx4
Soft
Stiff
Soft
Stiff
P-SREBP2
150kDa
100kDa
75kDa
50kDa
37kDa
β-tubulin
150kDa
100kDa
75kDa
50kDa
37kDa
N-SREBP2
150kDa
100kDa
75kDa
50kDa
37kDa
HMGCR
150kDa
100kDa
75kDa
50kDa
37kDa

Figure S7

A

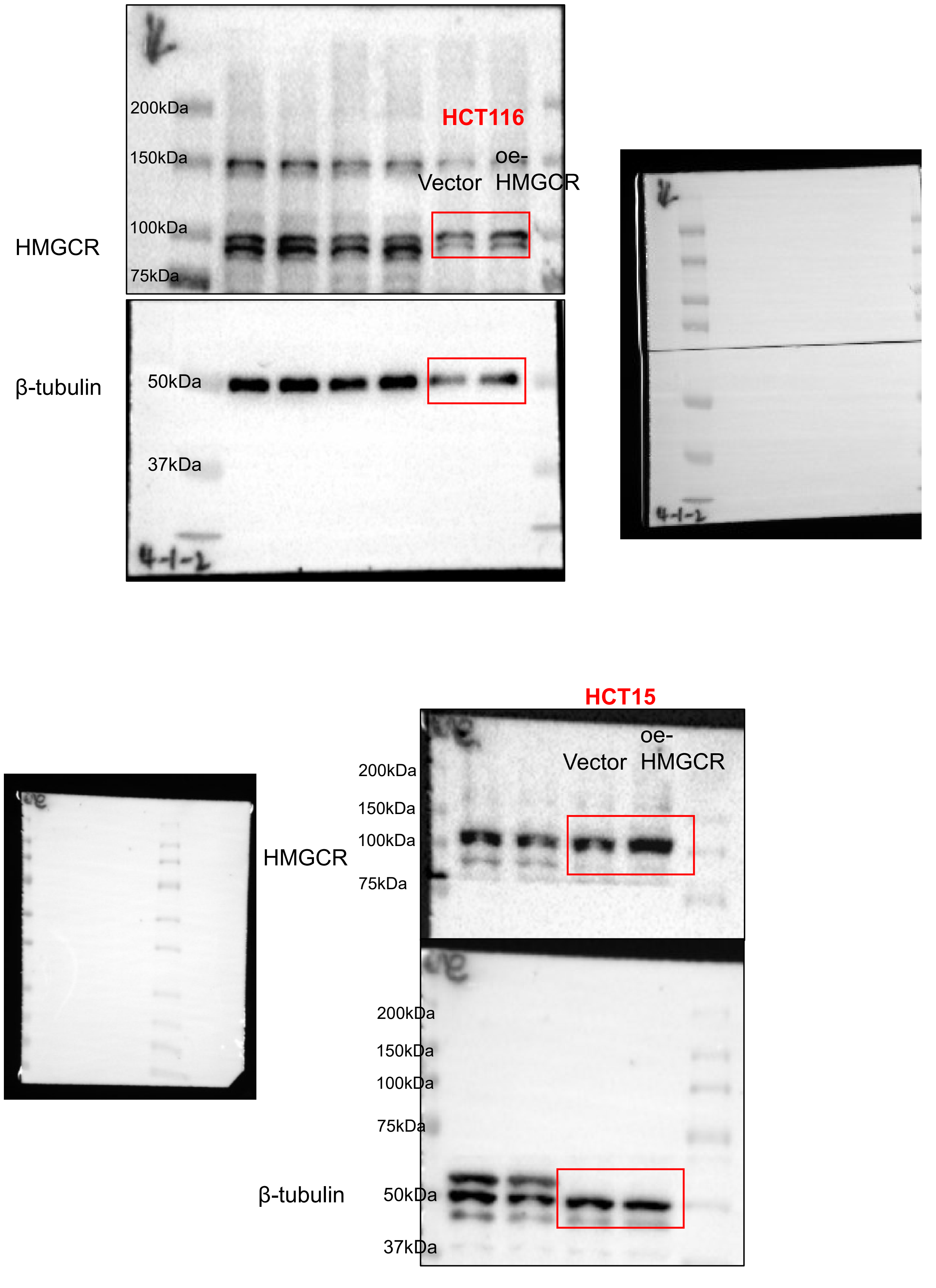
HCT116
HMGCR
β-tubulin
200kDa
150kDa
100kDa
75kDa
50kDa
37kDa
Vector
oe-HMGCR
4-1-2
HCT15
HMGCR
β-tubulin
200kDa
150kDa
100kDa
75kDa
50kDa
37kDa
Vector
oe-HMGCR

Figure S7

C

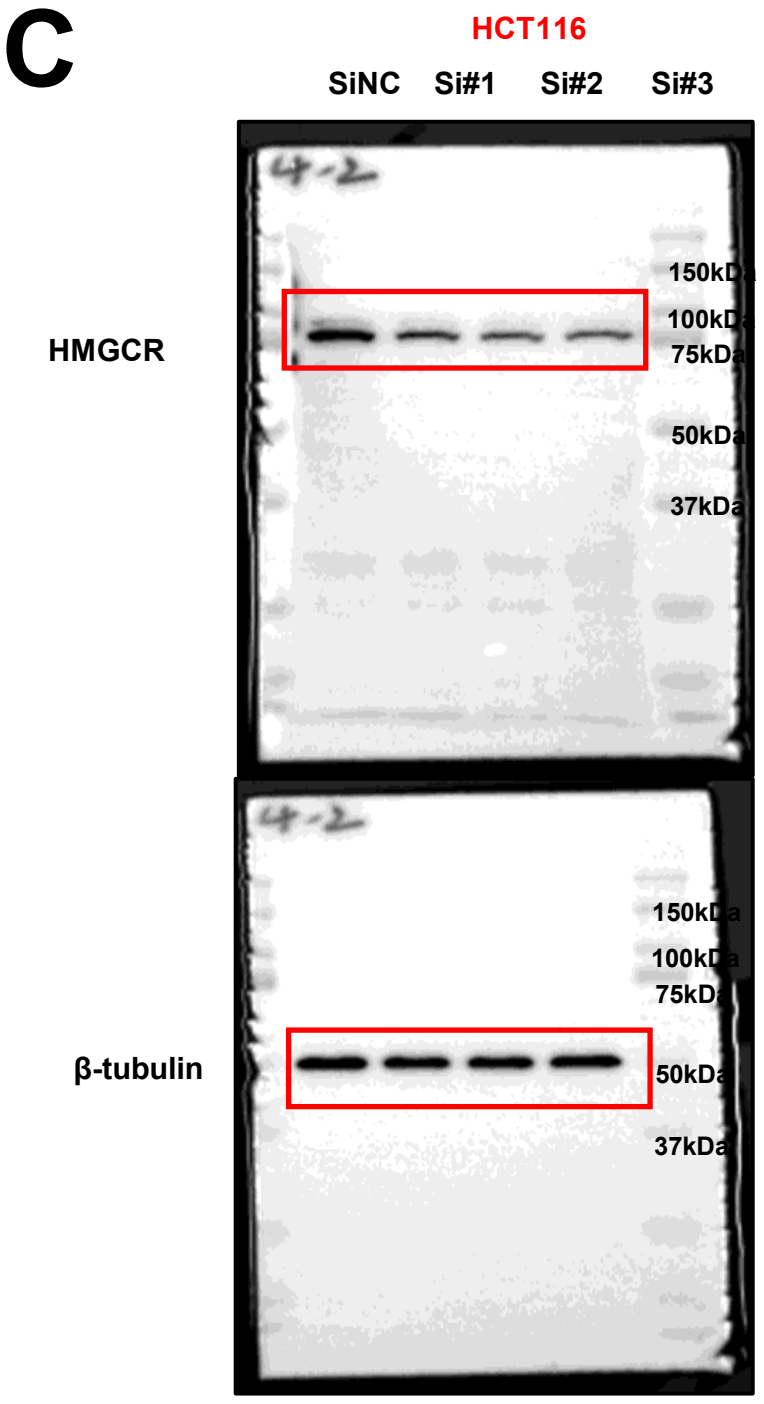
HCT116
SiNC
Si#1
Si#2
Si#3
HMGCR
150kDa
100kDa
75kDa
50kDa
37kDa
β-tubulin
150kDa
100kDa
75kDa
50kDa
37kDa

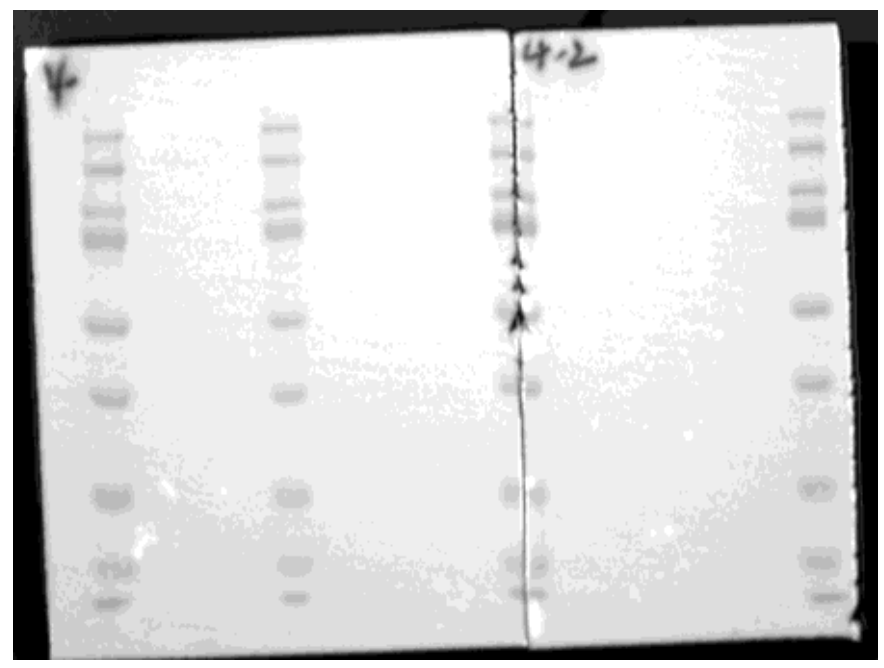

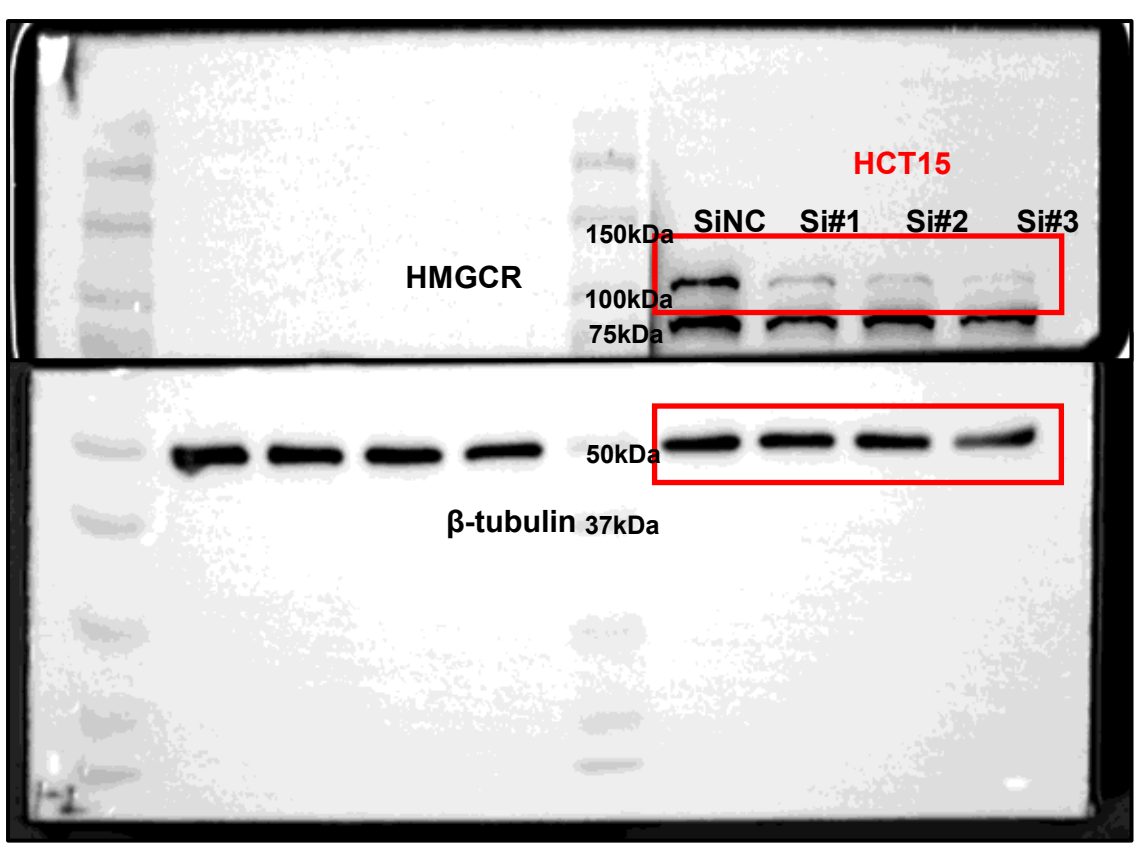
HCT15
SiNC
Si#1
Si#2
Si#3
HMGCR
150kDa
100kDa
75kDa
50kDa
β-tubulin
37kDa

Figure S10

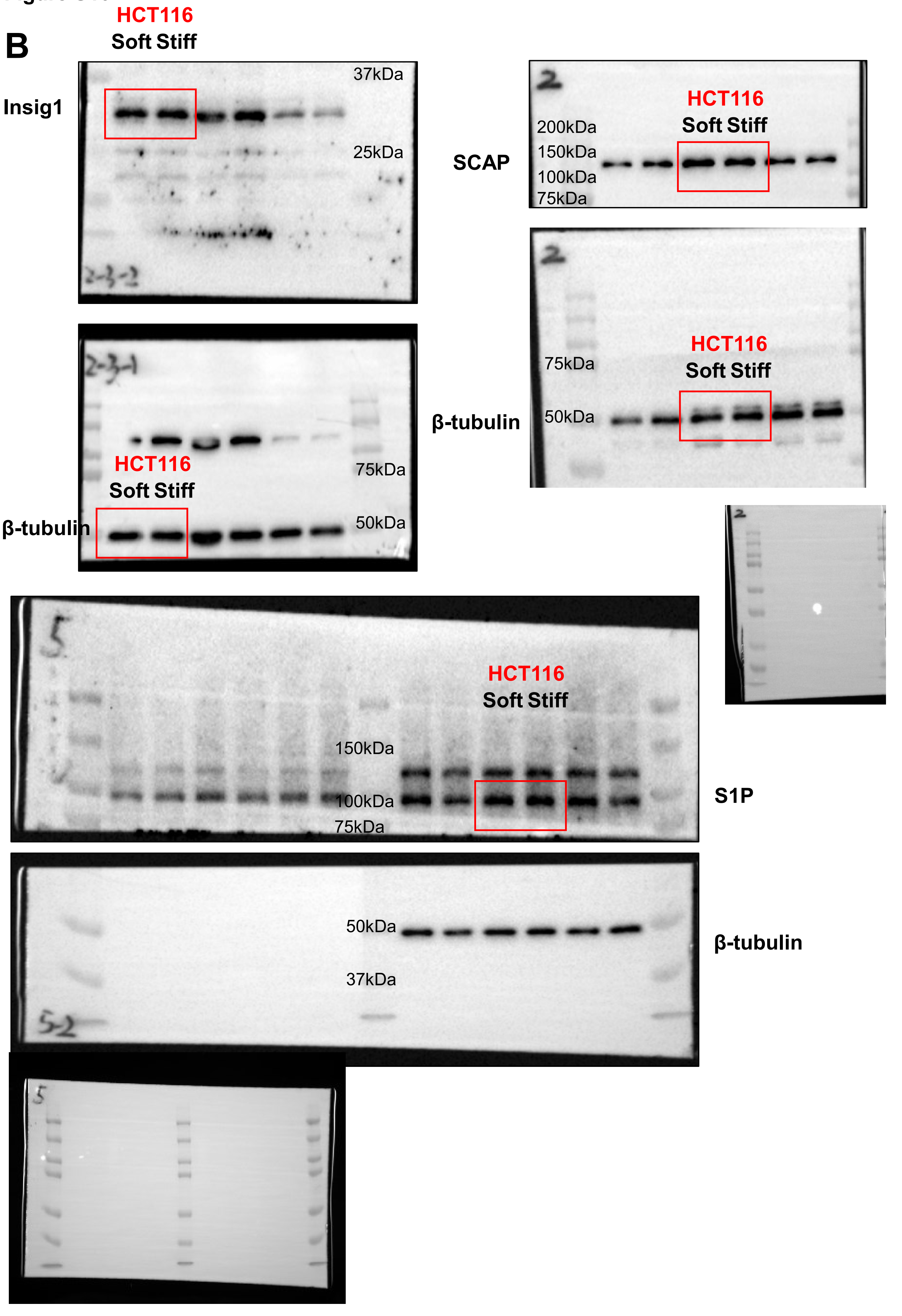
B
HCT116
Soft Stiff
Insig1
37kDa
25kDa
SCAP
HCT116
Soft Stiff
200kDa
150kDa
100kDa
75kDa
β-tubulin
HCT116
Soft Stiff
75kDa
50kDa
HCT116
Soft Stiff
β-tubulin
75kDa
50kDa
HCT116
Soft Stiff
150kDa
100kDa
75kDa
S1P
50kDa
37kDa
β-tubulin

Figure S10

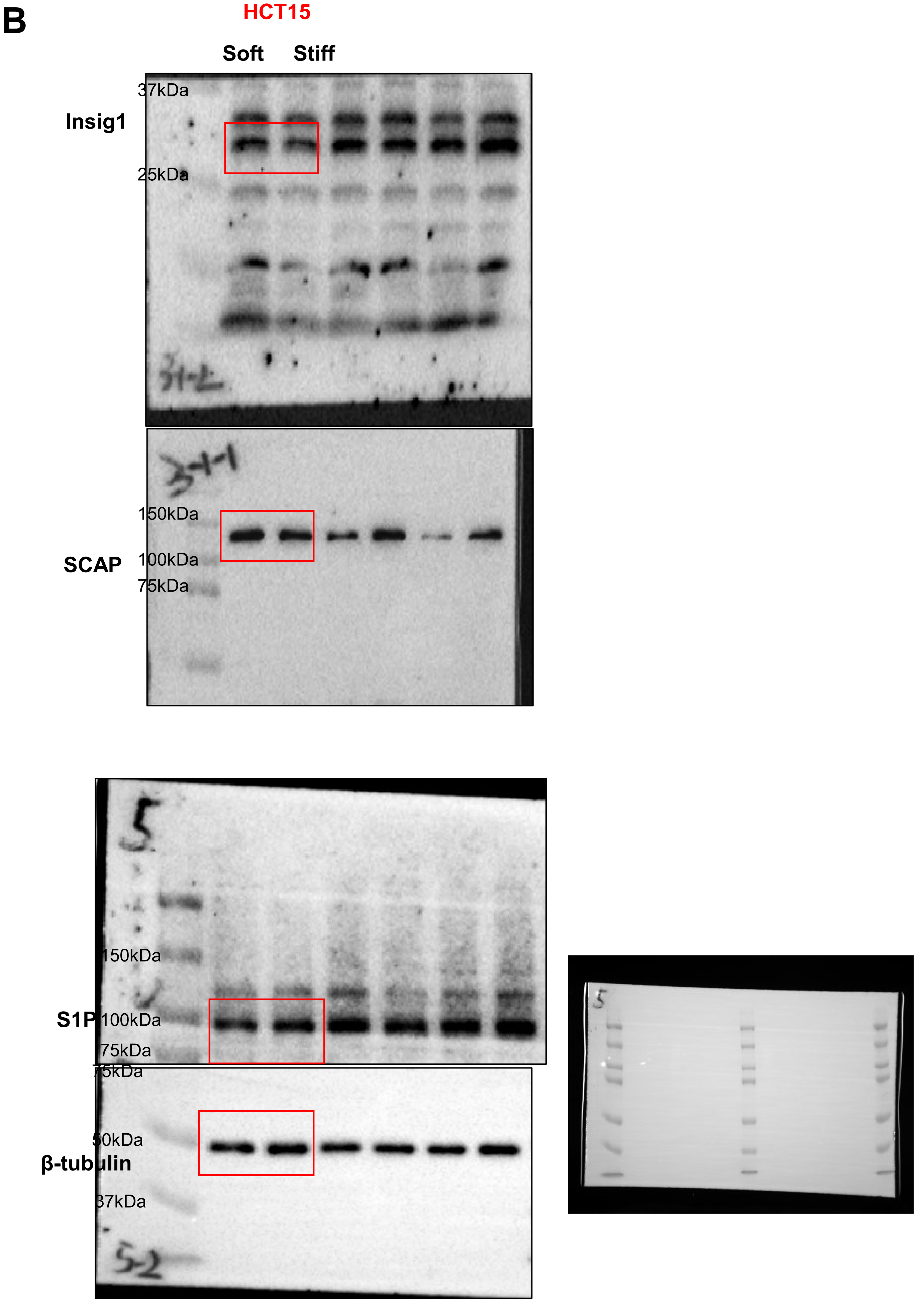
B
HCT15
Soft
Stiff
Insig1
37kDa
25kDa
SCAP
150kDa
100kDa
75kDa
S1P
150kDa
100kDa
75kDa
75kDa
β-tubulin
50kDa
37kDa
