# Supplementary material for: Matrix stiffness promotes colorectal cancer progression via Golgi-dependent cholesterol synthesis
Source: Cell Death Dis. 2026 Jul 24;17(1):894. doi: 10.1038/s41419-026-09101-5 (PMC13680387; doi:10.1038/s41419-026-09101-5)
Supplement: Supplementary file 3 [file 41419_2026_9101_MOESM1_ESM.docx]

**Table S1 The key resources of primer sequences**

| **Gene** | **Sequence-F** | **Sequence-R** |
| --- | --- | --- |
| HMGCR | ACAGATACTTGGGAATGCAGAG | CTGTCGGCGAATAGATACACC |
| LDLR | TTCACTCCATCTCAAGCATCG | ACTGAAAATGGCTTCGTTGATG |
| HMGCS1 | TTCCCTTGCATCTGTTCTAGC | TTTTATCAAGAGCAGACCCCG |
| E2F2 | CGTCCCTGAGTTCCCAACC | GCGAAGTGTCATACCGAGTCTT |
| E2F7 | TAGCTCGCTATCCAAGTTATCCC | CAATGTCATAGATGCGTCTCCTT |
| BRCA1 | TCACCCTCTGCTCTGGTTCA | AAAGGACACTGTGAAGGCCC |
| XRCC4 | ATGTTGGTGAACTGAGAAAAGCA | GCAATGGTGTCCAAGCAATAAC |
| SCAP | TATCTCGGGCCTTCTACAACC | GGGGCGAGTAATCCTTCACA |
| INSIG1 | CCTGGCATCATCGCCTGTT | AGAGTGACATTCCTCTGGATCTG |
| S1P | CCAGGGAAGGTGCTTATGGG | AGTGACAGACTTCTGCAGGC |
| SREBF2 | AACGGTCATTCACCCAGGTC | GGCTGAAGAATAGGAGTTGCC |
| S2P | TGGACTGTCGTCTACCTGACC | AGCAGTTTGCCATCTTATGTGG |
| GOLPH3 | CAAGGACCGCGAGGGTTAC | TTACGTCTCATTCCACAAGCC |
| STX3 | TCGGCAGACCTTCGGATTC | TCCTCATCGGTTGTCTTTTTGC |
| GM130 | CCCGCGATGTCGGAAGAAA | GCATTGTCCTTGGGTGTATCCT |
| WIPI1 | AACAGGTCTATGTGCTCTCTCT | CTCATGGGCAGCAATAGTGC |
| RAB20 | CTATGATGTGAATCACCGGCA | GGTCCCCAGCGTCCATATTG |
| Giantin | GTGCTCAGGTCGTTGACTTG | TGTTTCCACAGTGTTCCTCAAA |
| HMGCR-promoter | CGTCAGAAATGTGTGGTGGG | ACAGTGCAATAGCCTCTCCT |
|  | GGCTGCTAAGAGAACACTGC | CTGTGGTTGGCATGGGAGAT |

**Table S2 The key resources of antibodies**

| **Antibody** | **Brand** | **Code** |
| --- | --- | --- |
| E-cadherin | Proteintech | 20874-1-AP |
| Vimentin | Proteintech | 60330-1-Ig |
| SREBP2 | Novus | NBP1-54446 |
| SREBP2 | Novus | NB100-74543 |
| SREBP2 | Novus | AF7119 |
| N-cadherin | Abmart | T55015 |
| YAP1 | Proteintech | 13584-1-AP |
| YAP1 | Santa | sc-101199 |
| β-catenin | Abclonal | A11932 |
| HMGCS1 | Abclonal | A3916 |
| LDLR | Abclonal | A22454 |
| HMGCR | Abmart | T56640 |
| HA | Abmart | M20003 |
| Insig1 | Proteintech | 55282-1-AP |
| SCAP | Abmart | MA9380 |
| SCAP | Santa | sc-13553 |
| S1P | Abclonal | A16243 |
| KDEL | Abcam | ab176333 |
| GM130 | Proteintech | 66662-1-Ig |
| Flag | Abcam | ab205606 |
| β-tubulin | Proteintech | 66240-1-Ig |
| LaminB1 | Cell Signaling Technology | #13435 |
| HRP-goat anti-rabbit IgG | EARTHOX | E030120-01 |
| HRP-goat anti-mouse IgG | EARTHOX | E030110-01 |
| Rabbit IgG | Proteintech | 30000-0-AP |
| Mouse IgG | Santa | sc-515946 |

**Table S3 Clinical information of human subjects with CRC**

| **No.** | **T stage** | **N stage** | **M stage** | **AJCC clinical stage (NO.7)** |
| --- | --- | --- | --- | --- |
| **1** | **T1** | **N0** | **M0** | **1期** |
| **2** | **T1** | **N0** | **M0** | **1期** |
| **3** | **T2** | **N0** | **M0** | **1期** |
| **4** | **T2** | **N0** | **M0** | **1期** |
| **5** | **T3** | **N0** | **M0** | **2期** |
| **6** | **T3** | **N0** | **M0** | **2期** |
| **7** | **T4** | **N0** | **M0** | **2期** |
| **8** | **T2** | **N1** | **M0** | **3期** |
| **9** | **T2** | **N1** | **M0** | **3期** |
| **10** | **T2** | **N1** | **M0** | **3期** |
| **11** | **T3** | **N1** | **M0** | **3期** |
| **12** | **T3** | **N1** | **M0** | **3期** |
| **13** | **T3** | **N1** | **M0** | **3期** |
| **14** | **T3** | **N1** | **M0** | **3期** |
| **15** | **T3** | **N1** | **M0** | **3期** |
| **16** | **T4** | **N1** | **M0** | **3期** |
| **17** | **T3** | **N2** | **M0** | **3期** |
| **18** | **T4** | **N2** | **M0** | **3期** |
| **19** | **T4** | **N1** | **M1** | **4期** |
| **20** | **T3** | **N2** | **M1** | **4期** |

**Table S4 The differentially expressed genes of RNA-seq data of HCT116**

| **gene_id** | **gene_symbol** | **diffexp_degseq_pvalue_SOFT-vs-STIFF** |
| --- | --- | --- |
| 100129583 | FAM47E | 0.04824 |
| 348980 | HCN1 | 0.033871 |
| 80760 | ITIH5 | 0.046143 |
| 554235 | ASPDH | 0.046436 |
| 162515 | SLC16A11 | 0.024281 |
| 10161 | LPAR6 | 0.002691 |
| 1950 | EGF | 0.008314 |
| 56924 | PAK6 | 3.69E-08 |
| 91624 | NEXN | 0.04845 |
| 343990 | CRACDL | 1.48E-05 |
| 26266 | SLC13A4 | 0.002911 |
| 7439 | BEST1 | 1.49E-11 |
| 165 | AEBP1 | 0.024643 |
| 10942 | PRSS21 | 0.046436 |
| 91543 | RSAD2 | 0.044948 |
| 219348 | PLAC9 | 0.048334 |
| 59335 | PRDM12 | 0.032569 |
| 124902551 | LOC124902551 | 0.044417 |
| 83729 | INHBE | 0.000139 |
| 10990 | LILRB5 | 0.014188 |
| 80114 | BICC1 | 0.002839 |
| 50615 | IL21R | 0.005254 |
| 27071 | DAPP1 | 0.000114 |
| 101929726 | MYMX | 0.039961 |
| 3231 | HOXD1 | 0.025795 |
| 285800 | PRR18 | 0.039961 |
| 124901652 | LOC124901652 | 0.008921 |
| 3593 | IL12B | 0.029296 |
| 102724474 | LOC102724474 | 0.044948 |
| 124902536 | LOC124902536 | 0.041301 |
| 642659 | HNRNPA1L3 | 0.000258 |
| 143379 | C10orf82 | 0.027676 |
| 256710 | GLIPR1L1 | 0.048334 |
| 3576 | CXCL8 | 9.05E-12 |
| 6323 | SCN1A | 0.018975 |
| 5133 | PDCD1 | 0.010472 |
| 27098 | CLUL1 | 0.004709 |
| 4909 | NTF4 | 0.006644 |
| 124904176 | LOC124904176 | 0.002839 |
| 55195 | CCDC198 | 0.007326 |
| 132228 | LSMEM2 | 0.041707 |
| 8013 | NR4A3 | 1.60E-12 |
| 874 | CBR3 | 7.15E-05 |
| 9963 | SLC23A1 | 0.029069 |
| 1959 | EGR2 | 2.06E-22 |
| 10803 | CCR9 | 0.021148 |
| 390748 | PABPN1L | 0.049237 |
| 51655 | RASD1 | 0.042666 |
| 259292 | TAS2R46 | 0.048334 |
| 3815 | KIT | 0.048334 |
| 29942 | PURG | 0.027676 |
| 377007 | KLHL30 | 0.017701 |
| 81030 | ZBP1 | 0.021148 |
| 79946 | C10orf95 | 0.011022 |
| 6781 | STC1 | 0.002691 |
| 948 | CD36 | 0.039081 |
| 56128 | PCDHB8 | 0.02849 |
| 100526772 | STIMATE-MUSTN1 | 0.003151 |
| 7306 | TYRP1 | 0.025795 |
| 22953 | P2RX2 | 0.013749 |
| 124602 | KIF19 | 0.029069 |
| 10930 | APOBEC2 | 0.041301 |
| 128553 | TSHZ2 | 0.038153 |
| 3040 | HBA2 | 0.000613 |
| 64072 | CDH23 | 0.030151 |
| 56142 | PCDHA6 | 0.01284 |
| 6870 | TACR3 | 0.013749 |
| 127833 | SYT2 | 0.012193 |
| 57158 | JPH2 | 0.004053 |
| 286343 | LURAP1L | 1.40E-10 |
| 84868 | HAVCR2 | 0.042309 |
| 9724 | UTP14C | 2.10E-05 |
| 197135 | PATL2 | 0.016793 |
| 56159 | TEX11 | 0.030257 |
| 400935 | IL17REL | 0.024643 |
| 200159 | C1orf100 | 0.025795 |
| 1135 | CHRNA2 | 0.041301 |
| 100134444 | KCNJ18 | 0.01664 |
| 875 | CBS | 0.015599 |
| 27288 | RBMXL2 | 0.006579 |
| 79788 | ZNF665 | 0.020147 |
| 100533467 | BIVM-ERCC5 | 6.07E-06 |
| 113730 | KLHDC7B | 1.93E-11 |
| 102724594 | LOC102724594 | 2.09E-103 |
| 102723382 | LOC102723382 | 0.024281 |
| 126272 | EID2B | 0.017042 |
| 107986982 | LOC107986982 | 0.008164 |
| 100526740 | ATP5MF-PTCD1 | 7.10E-05 |
| 157777 | MCMDC2 | 0.0039 |
| 255101 | CFAP65 | 0.01106 |
| 414060 | TBC1D3C | 4.65E-12 |
| 220074 | LRTOMT | 0.009518 |
| 202658 | TRIM39-RPP21 | 0.005447 |
| 57336 | ZNF287 | 0.044944 |
| 124902704 | LOC124902704 | 0.044944 |
| 8637 | EIF4EBP3 | 0.01218 |
| 124904774 | LOC124904774 | 0.02618 |
| 387911 | C1QTNF9B | 0.026759 |
| 27121 | DKK4 | 0.017303 |
| 107987020 | LOC107987020 | 0.039475 |
| 282973 | JAKMIP3 | 0.033289 |
| 6328 | SCN3A | 0.029404 |
| 341 | APOC1 | 0.018394 |
| 752014 | CEMP1 | 0.000891 |
| 56103 | PCDHGB2 | 0.02778 |
| 8404 | SPARCL1 | 0.012437 |
| 117144 | CATSPER1 | 0.006071 |
| 129025 | ZNF280A | 0.001551 |
| 153643 | FAM81B | 0.0366 |
| 389421 | LIN28B | 0.02133 |
| 80763 | SPX | 0.000542 |
| 3604 | TNFRSF9 | 0.044032 |
| 107080638 | TBC1D7-LOC100130357 | 0.013691 |
| 56123 | PCDHB13 | 0.002582 |
| 644100 | ARL14EPL | 1.60E-17 |
| 101059918 | GOLGA8R | 8.52E-05 |
| 6286 | S100P | 0.001698 |
| 139212 | DNAAF6 | 0.027676 |
| 63876 | PKNOX2 | 0.00995 |
| 9256 | TSPOAP1 | 0.006736 |
| 144501 | KRT80 | 4.88E-09 |
| 57624 | NYAP2 | 0.024732 |
| 124901225 | LOC124901225 | 0.002188 |
| 83888 | FGFBP2 | 0.014764 |
| 128229 | TSACC | 0.023157 |
| 503582 | ARGFX | 0.039961 |
| 124907920 | LOC124907920 | 0.044948 |
| 222553 | SLC35F1 | 0.001188 |
| 124900997 | LOC124900997 | 1.28E-05 |
| 84808 | PERM1 | 0.047876 |
| 360205 | PRAC2 | 0.041301 |
| 85480 | TSLP | 0.035223 |
| 284417 | TMEM150B | 0.004053 |
| 151449 | GDF7 | 0.012591 |
| 80201 | HKDC1 | 2.91E-11 |
| 3357 | HTR2B | 0.02768 |
| 4112 | MAGEB1 | 0.013749 |
| 254778 | VXN | 0.044496 |
| 8741 | TNFSF13 | 3.72E-05 |
| 683 | BST1 | 0.016834 |
| 124903372 | LOC124903372 | 0.016001 |
| 3866 | KRT15 | 1.18E-07 |
| 3371 | TNC | 0.00995 |
| 11219 | TREX2 | 0.005416 |
| 3078 | CFHR1 | 0.03712 |
| 53833 | IL20RB | 6.33E-25 |
| 81033 | KCNH6 | 0.046436 |
| 344387 | CDKL4 | 0.039961 |
| 79983 | POF1B | 0.029404 |
| 100131378 | C11orf91 | 0.041856 |
| 102800317 | TPTEP2-CSNK1E | 0.008316 |
| 347736 | NME9 | 0.030476 |
| 92291 | CAPN13 | 0.029767 |
| 100532736 | MICOS10-NBL1 | 6.30E-12 |
| 127534 | GJB4 | 0.047039 |
| 152098 | ZCWPW2 | 0.000179 |
| 257177 | CFAP126 | 0.005132 |
| 27063 | ANKRD1 | 0.000113 |
| 225 | ABCD2 | 0.044183 |
| 8190 | MIA | 0.029925 |
| 111089941 | MMP24-AS1-EDEM2 | 0.000882 |
| 563 | AZGP1 | 0.043119 |
| 2911 | GRM1 | 0.014764 |
| 100631383 | FAM47E-STBD1 | 0.000112 |
| 2769 | GNA15 | 0.028674 |
| 25840 | TMT1A | 1.25E-08 |
| 124903452 | LOC124903452 | 0.005953 |
| 6374 | CXCL5 | 0.002691 |
| 115072896 | SLCO1B3-SLCO1B7 | 0.000533 |
| 2253 | FGF8 | 0.007714 |
| 92691 | TMEM169 | 0.014304 |
| 55080 | TAPBPL | 0.013749 |
| 3117 | HLA-DQA1 | 0.024643 |
| 728492 | SERF1B | 2.22E-09 |
| 3577 | CXCR1 | 0.027676 |
| 6579 | SLCO1A2 | 0.012586 |
| 56911 | MAP3K7CL | 0.042309 |
| 2838 | GPR15 | 3.11E-05 |
| 23569 | PADI4 | 0.01289 |
| 441521 | CT45A5 | 0.045542 |
| 3640 | INSL3 | 0.017343 |
| 650 | BMP2 | 0.005553 |
| 166336 | PRICKLE2 | 0.031456 |
| 79153 | GDPD3 | 0.028325 |
| 8363 | H4C11 | 0.002651 |
| 27145 | FILIP1 | 0.036695 |
| 100287513 | USP17L22 | 0.033053 |
| 3620 | IDO1 | 0.009254 |
| 4105 | MAGEA6 | 0.040811 |
| 140825 | NEURL2 | 0.020527 |
| 3488 | IGFBP5 | 0.000492 |
| 7173 | TPO | 0.010472 |
| 7425 | VGF | 7.82E-53 |
| 347475 | CCDC160 | 0.019229 |
| 6252 | RTN1 | 0.016278 |
| 54857 | GDPD2 | 0.048334 |
| 58511 | DNASE2B | 0.03348 |
| 285190 | RGPD4 | 0.004011 |
| 79444 | BIRC7 | 0.049242 |
| 27189 | IL17C | 0.025795 |
| 3892 | KRT86 | 0.047484 |
| 64168 | NECAB1 | 0.018609 |
| 57010 | CABP4 | 0.016586 |
| 93432 | MGAM2 | 0.0197 |
| 2047 | EPHB1 | 0.023791 |
| 124901030 | LOC124901030 | 0.036994 |
| 84913 | ATOH8 | 0.000439 |
| 64577 | ALDH8A1 | 0.001079 |
| 221421 | RSPH9 | 0.040227 |
| 6795 | AURKC | 0.02231 |
| 124907867 | LOC124907867 | 0.000777 |
| 51351 | ZNF117 | 3.56E-23 |
| 6818 | SULT1A3 | 7.15E-14 |
| 646658 | SYNDIG1L | 0.036578 |
| 27156 | RSPH14 | 0.011303 |
| 142685 | ASB15 | 0.020361 |
| 441327 | FAM90A9 | 0.009116 |
| 3039 | HBA1 | 3.61E-09 |
| 100528021 | ST20-MTHFS | 3.37E-05 |
| 340273 | ABCB5 | 0.013064 |
| 9718 | ECE2 | 0.044948 |
| 219537 | SMTNL1 | 0.042309 |
| 26025 | PCDHGA12 | 0.028573 |
| 4588 | MUC6 | 0.044813 |
| 364 | AQP7 | 0.000609 |
| 720 | C4A | 0.021711 |
| 101928788 | ARL4AP5 | 0.030117 |
| 338382 | RAB7B | 0.006253 |
| 401089 | FOXL2NB | 0.023787 |
| 3543 | IGLL1 | 0.0366 |
| 10738 | RFPL3 | 0.016022 |
| 3266 | ERAS | 0.020361 |
| 4049 | LTA | 0.018975 |
| 255809 | C19orf38 | 0.007117 |
| 4633 | MYL2 | 0.015851 |
| 254158 | CXorf58 | 0.031244 |
| 154313 | CFAP206 | 0.035683 |
| 11185 | INMT | 0.019815 |
| 4929 | NR4A2 | 8.08E-81 |
| 1117 | CHI3L2 | 0.039961 |
| 201456 | FBXO15 | 0.000504 |
| 3375 | IAPP | 0.041301 |
| 3221 | HOXC4 | 0.000483 |
| 100288814 | CLDN34 | 0.010928 |
| 56114 | PCDHGA1 | 0.01565 |
| 29108 | PYCARD | 0.027676 |
| 445372 | TRIM6-TRIM34 | 0.006357 |
| 151647 | TAFA4 | 0.027713 |
| 390063 | OR51I1 | 0.006867 |
| 401546 | C9orf152 | 0.027676 |
| 3111 | HLA-DOA | 0.001536 |
| 654483 | BOLA2B | 3.78E-10 |
| 23120 | ATP10B | 0.039961 |
| 6703 | SPRR2D | 2.02E-07 |
| 11141 | IL1RAPL1 | 0.025324 |
| 125467750 | EFNA4-EFNA3 | 3.83E-08 |
| 124901355 | LOC124901355 | 0.029296 |
| 112267968 | LOC112267968 | 0.041301 |
| 2006 | ELN | 0.016611 |
| 124904333 | LOC124904333 | 0.00195 |
| 653499 | LGALS7B | 0.042386 |
| 25924 | MYRIP | 0.000391 |
| 378925 | RNF148 | 0.024643 |
| 9048 | ARTN | 6.86E-09 |
| 80326 | WNT10A | 0.036992 |
| 3855 | KRT7 | 1.58E-24 |
| 124903211 | LOC124903211 | 0.003583 |
| 388931 | MFSD2B | 0.048334 |
| 213 | ALB | 0.020308 |
| 64005 | MYO1G | 0.005795 |
| 6833 | ABCC8 | 0.020361 |
| 2527 | FUT5 | 0.041301 |
| 126206 | NLRP5 | 0.000515 |
| 84217 | ZMYND12 | 0.017701 |
| 23475 | QPRT | 0.032134 |
| 256979 | SUN3 | 0.005471 |
| 100529257 | SYNJ2BP-COX16 | 0.037844 |
| 100287284 | MANSC4 | 0.046994 |
| 339977 | LRRC66 | 0.002602 |
| 7378 | UPP1 | 2.27E-30 |
| 3887 | KRT81 | 0.000823 |
| 121340 | SP7 | 0.048334 |
| 54538 | ROBO4 | 0.013749 |
| 197021 | LCTL | 0.032522 |
| 10616 | RBCK1 | 8.89E-110 |
| 56147 | PCDHA1 | 0.016741 |
| 124909474 | LOC124909474 | 0.041961 |
| 60676 | PAPPA2 | 0.028597 |
| 1520 | CTSS | 0.0336 |
| 401036 | ASB18 | 0.009352 |
| 828 | CAPS | 0.000489 |
| 727 | C5 | 0.01715 |
| 8288 | EPX | 0.009196 |
| 128966726 | LOC128966726 | 0.035996 |
| 54065 | SMIM11 | 2.73E-08 |
| 1114 | CHGB | 0.041301 |
| 353135 | LCE1E | 0.020361 |
| 94137 | RP1L1 | 0.029404 |
| 653075 | GOLGA8T | 0.002691 |
| 9201 | DCLK1 | 9.25E-08 |
| 347733 | TUBB2B | 0.021937 |
| 64805 | P2RY12 | 0.04868 |
| 285386 | TPRG1 | 0.002379 |
| 84171 | LOXL4 | 1.62E-05 |
| 340351 | AGBL3 | 0.002265 |
| 100533496 | TVP23C-CDRT4 | 1.57E-17 |
| 51561 | IL23A | 0.032091 |
| 388531 | RGS9BP | 0.047863 |
| 124900576 | LOC124900576 | 0.041301 |
| 124904958 | LOC124904958 | 0.028312 |
| 57761 | TRIB3 | 1.64E-118 |
| 2147 | F2 | 0.003672 |
| 4922 | NTS | 0.046436 |
| 3626 | INHBC | 0.015731 |
| 201798 | TIGD4 | 0.036986 |
| 148398 | SAMD11 | 1.72E-11 |
| 4986 | OPRK1 | 0.048334 |
| 375307 | CATIP | 0.046436 |
| 3293 | HSD17B3 | 0.035574 |
| 89876 | CFAP91 | 0.011546 |
| 8653 | DDX3Y | 0.020143 |
| 126014 | OSCAR | 0.043611 |
| 4583 | MUC2 | 0.024281 |
| 27178 | IL37 | 0.029296 |
| 100527943 | TGIF2-RAB5IF | 0.000805 |

**Supplementary figure legends**

**Figure S1 The matrix stiffness patterns in CRC based on RNA-seq and scRNA-seq data. A.** The heatmap of differentially expressed genes among the Soft and Stiff group based on matrix stiffness (MS) score in TCGA-COAD dataset. **B.** The cluster plot of distinguished cell types of CRC tissues from four GEO databases. **C.** The plot of specific gene markers of distinguished cell types of CRC tissues from four GEO databases. **D.** The cluster plot of distinguished malignant epithelial cells. **E.** The heatmap of the distinguished clusters of malignant epithelial cells.

**Figure S2 Matrix stiffening promotes epithelial-mesenchymal transition in CRC. A&B.** The proliferation area (A) and axial ratio (B) of HCT116 and HCT15 under different stiffness. **C.** The representative immunofluorescence images staining with Vimentin in HCT116 and HCT15 under different stiffness. **D.** The relative protein levels of E-cadherin in HCT116 and HCT15 under different stiffness. **E.** The H&E staining of the orthotopic CRC xenograft tissues.

**Figure S3 Bioinformatic analysis of RNA-seq from TCGA-COAD population. A.** The volcano plot of differentially expressed genes of two clusters. **B.** The correlation analysis of expression levels of EMT-pathway-related genes and cholesterol-homeostasis-pathway-related genes. **C.** The correlation analysis of enrichment scores of EMT and cholesterol homeostasis pathways.

**Figure S4**. **Matrix stiffness promotes cholesterol levels.** **A.** The total cholesterol levels of HCT116 and HCT15 under different stiffness. **B.** The serum cholesterol levels of CRC mice models. **C.** The total cholesterol levels of HCT116 and HCT15 treated with Fatostatin (10 μM). **D.** The total cholesterol levels of HCT116 and HCT15 under different stiffness treated with Fatostatin (10 μM).

**Figure S5 Matrix stiffness regulates cholesterol biosynthesis-related genes expression. A.** The mRNA levels of HMGCR, LDLR, HMGCS1 in HCT116, HCT15, SW480, HT29 cells under different stiffness. **B.** The relative protein level of HMGCR in the orthotopic CRC xenograft tissues and DSS/AOM-induced CRC tissues.

**Figure S6 The expression of HMGCR in colorectal cancer tissues and normal intestinal tissues from TCGA and GTEx.**

**Figure S7 The effect of HMGCR on CRC cells. A&B.** The Western blot analysis (A) and mRNA level (B) of HMGCR in HCT116 and HCT15 transfected with HMGCR plasmids. **C&D.** The Western blot analysis (C) and mRNA level (D) of HMGCR in HCT116 and HCT15 transfected with si-HMGCR. **E&F.** The cholesterol level of HCT116 and HCT15 with overexpression of HMGCR (E) and inhibition of HMGCR using Simvastatin (F). **G&H&I.** HCT116 and HCT15 cells were transfected with si-HMGCR. The cells were harvested for CCK8 (G), EdU (H), and Transwell (I).

**Figure S8 The total cholesterol levels of HCT116 and HCT15 under different stiffness treated with Simvastatin.**

**Figure S9 The relative mRNA level of SREBF2 in HCT116 and HCT15 cultured on different stiffness of PA hydrogels.**

**Figure S10 The mRNA levels (A) and protein levels (B) of SCAP, Insig1, S1P and S2P in HCT116 and HCT15 cultured on different stiffness of PA hydrogels.**

**Figure S11 The mRNA levels of Golgi structural and trafficking components in HCT116 and HCT15 cultured on different stiffness of PA hydrogels.**

**Figure S12 The representative confocal images of Golgi and ER in HCT116 and HCT15 treated with BFA.**

**Figure S13 The relative mRNA level of HMGCR, HMGCS1 and LDLR in HCT116 and HCT15** **treated with BFA.**

**Figure S14 The relative mRNA level of Golgi-related genes in HCT116 and HCT15** **treated with Yoda1.**
